# Lipid disturbances induced by psychotropic drugs: clinical and genetic predictors for early worsening of lipid levels and new-onset dyslipidaemia in Swiss psychiatric samples

**DOI:** 10.1192/bjo.2024.757

**Published:** 2024-12-05

**Authors:** Aurélie Delacrétaz, Marie Sadler, Franziska Gamma, Martin Preisig, Hélène Richard-Lepouriel, Armin von Gunten, Philippe Conus, Kerstin Jessica Plessen, Zoltan Kutalik, Chin B. Eap

**Affiliations:** Unit of Pharmacogenetics and Clinical Psychopharmacology, Centre for Psychiatric Neuroscience, Department of Psychiatry, Lausanne University Hospital, Lausanne, Switzerland; and Les Toises Psychiatry and Psychotherapy Center, Lausanne, Switzerland; Swiss Institute of Bioinformatics, Lausanne, Switzerland; University Center for Primary Care and Public Health, University of Lausanne, Lausanne, Switzerland; and Department of Computational Biology, University of Lausanne, Lausanne, Switzerland; Les Toises Psychiatry and Psychotherapy Center, Lausanne, Switzerland; Center for Research in Psychiatric Epidemiology and Psychopathology, Department of Psychiatry, Lausanne University Hospital and University of Lausanne, Lausanne, Switzerland; Unit of Mood Disorders, Department of Psychiatry, Geneva University Hospital, Geneva, Switzerland; Service of Old Age Psychiatry, Department of Psychiatry, Lausanne University Hospital, Prilly, Switzerland; Service of General Psychiatry, Department of Psychiatry, Lausanne University Hospital, Prilly, Switzerland; Division of Child and Adolescent Psychiatry, Department of Psychiatry, Lausanne University Hospital and University of Lausanne, Lausanne, Switzerland; Unit of Pharmacogenetics and Clinical Psychopharmacology, Centre for Psychiatric Neuroscience, Department of Psychiatry, Lausanne University Hospital, Lausanne, Switzerland; Center for Research and Innovation in Clinical Pharmaceutical Sciences, University of Lausanne, Lausanne, Switzerland; School of Pharmaceutical Sciences, University of Geneva, Geneva, Switzerland; and Institute of Pharmaceutical Sciences of Western Switzerland, University of Geneva, Geneva, Switzerland

**Keywords:** Antipsychotics, antidepressants, genetics, longitudinal data, risk assessment

## Abstract

**Background:**

Early worsening of plasma lipid levels (EWL; ≥5% change after 1 month) induced by at-risk psychotropic treatments predicts considerable exacerbation of plasma lipid levels and/or dyslipidaemia development in the longer term.

**Aims:**

We aimed to determine which clinical and genetic risk factors could predict EWL.

**Method:**

Predictive values of baseline clinical characteristics and dyslipidaemia-associated single nucleotide polymorphisms (SNPs) on EWL were evaluated in a discovery sample (*n* = 177) and replicated in two samples from the same cohort (PsyMetab; *n*_1_ = 176; *n*_2_ = 86).

**Results:**

Low baseline levels of total cholesterol, low-density lipoprotein cholesterol (LDL-C) and triglycerides, and high baseline levels of high-density lipoprotein cholesterol (HDL-C), were risk factors for early increase in total cholesterol (*P* = 0.002), LDL-C (*P* = 0.02) and triglycerides (*P* = 0.0006), and early decrease in HDL-C (*P* = 0.04). Adding genetic parameters (*n* = 17, 18, 19 and 16 SNPs for total cholesterol, LDL-C, HDL-C and triglycerides, respectively) improved areas under the curve for early worsening of total cholesterol (from 0.66 to 0.91), LDL-C (from 0.62 to 0.87), triglycerides (from 0.73 to 0.92) and HDL-C (from 0.69 to 0.89) (*P* ≤ 0.00003 in discovery sample). The additive value of genetics to predict early worsening of LDL-C levels was confirmed in two replication samples (*P* ≤ 0.004). In the combined sample (*n* ≥ 203), adding genetics improved the prediction of new-onset dyslipidaemia for total cholesterol, LDL-C and HDL-C (*P* ≤ 0.04).

**Conclusions:**

Clinical and genetic factors contributed to the prediction of EWL and new-onset dyslipidaemia in three samples of patients who started at-risk psychotropic treatments. Future larger studies should be conducted to refine SNP estimates to be integrated into clinically applicable predictive models.

Individuals suffering from mental disorders die at least 10 years earlier than the general population, mainly because of cardiovascular diseases resulting from metabolic syndrome.^1^ Multiple genetic and environmental risk factors may contribute to the observed excessive propensity for developing metabolic diseases in psychiatric patients. Other factors include psychiatric disease-related (e.g. unhealthy lifestyle, including low physical activity and/or poor diet combined with a restricted access to somatic care) and metabolic effects of treatment.^2^ For instance, the use of psychotropic medications, such as antipsychotics, mood stabilisers (e.g. lithium and valproate) and some antidepressants (e.g. mirtazapine), can significantly increase the risk of developing metabolic disorders, including obesity and dyslipidemia.^3^ As dyslipidaemia (defined as high total cholesterol and/or low-density lipoprotein cholesterol (LDL-C) and/or triglyceride and/or low high-density lipoprotein cholesterol (HDL-C) levels) constitutes a major risk factor for cardiovascular diseases,^4^ a close and prospective monitoring of metabolic parameters, including blood lipid levels, is required when introducing any psychotropic treatment associated with a metabolic risk.^5^ Psychotropic drug-induced dyslipidaemia has long been considered as resulting from psychotropic drug-induced weight gain. However, alterations of the lipid profile may occur very early during the psychotropic treatment (and may even precede weight gain), which can initiate a steady process leading to cardiometabolic diseases in the long term.^6^ In that context, 5% or more worsening of total cholesterol, LDL-C, HDL-C and/or triglyceride levels during the first month of psychotropic treatment at risk for inducing metabolic disturbances have been previously identified as best predictors for clinically relevant worsening of blood lipid levels after 3 months of treatment and for dyslipidaemia incidence in the longer term.^7^

Plasma lipid levels and/or dyslipidaemia are mostly determined by clinical and genetic factors.^8^ Although some forms of monogenic dyslipidaemia have been reported, most prevalent forms of dyslipidaemia are polygenic and result from the combination of multiple common genetic variants.^8^ A study conducted in psychiatric patients receiving psychotropic drugs inducing metabolic disturbances observed that polygenic risk scores (PRSs) combining lipid-associated single nucleotide polymorphisms (SNPs) from two population-based studies^9,10^ were significantly associated with plasma lipid levels, but could not predict the incidence of new-onset dyslipidaemia (NOD).^11^ With the rapid emergence of technical innovation in genotyping tools (e.g. genome-wide association studies (GWAS)) and exponential decrease in costs, many studies were conducted to identify new SNPs associated with lipid phenotypes. Since the previously mentioned study,^11^ six additional population-based GWAS meta-analyses of lipid levels have been reported, which further expanded the panel of available lipid-associated genetic variants.^12–17^ Since the past two decades, a growing interest in genetics in clinical settings has emerged. Recently, a randomised controlled trial on the clinical utility of a pre-emptive pharmacogenetic panel on the reduction of drug side-effects was conducted. This recent European trial showed that pharmacogenetic-guided prescribing resulted in a 30% reduction of clinically relevant adverse drug reactions and emphasised the feasibility and benefits of the consideration of genetic factors across diverse healthcare systems.^18^ As cardiovascular diseases are the main cause of premature mortality in the psychiatric population, it is of major clinical relevance for prescribers to identify predictive tools (e.g. an earliest detection, even before starting psychotropic treatment) that could help minimise and/or prevent early worsening of lipid levels (EWL) and/or NOD during psychotropic treatment. In that context, the present study aimed to determine whether the consideration of baseline clinical risk factors and lipid-associated genetic risk factors could predict EWL in patients who start a psychotropic medication associated with a risk of lipid worsening.

## Method

### Psychiatric samples

Patients were selected from a previously published prospective observational study conducted between 2007 and 2024 (PsyMetab), on the basis of clinical guidelines requiring metabolic follow-up after starting or switching to another psychotropic treatment (antipsychotics, mood stabilisers or certain antidepressants, i.e. mirtazapine or tricyclic antidepressants listed in Supplementary Table 1 available at https://doi.org/10.1192/bjo.2024.757), as described in the flowchart (Supplementary Fig. 1). Lipid variables (i.e. total cholesterol, LDL-C, HDL-C, LDL-C and triglycerides) were collected on a regular basis, at baseline (i.e. before psychotropic treatment introduction) and after 1, 3 and 12 months of treatment, and then yearly. Patients without available prospective lipid values were excluded from the analyses. They were included in the present study only when adherence was ascertained by therapeutic drug monitoring. Further details are described in the Supplementary Material.

A total of 439 patients who met the inclusion criteria were included in the present study (Supplementary Fig. 1). Patients genotyped using the Global Screening Array (GSA) version 2 were randomly allocated to the discovery (*n* = 177) or the replication sample (*n* = 176) (by using the rbinom function in R, statistical software for Windows, version 4.1.1 (R Foundation for Statistical Computing, Vienna, Austria; see https://www.r-project.org)), whereas patients genotyped using the third version of the GSA were included in replication sample 2 (*n* = 86). The splitting was particularly important to demonstrate the out-of-sample prediction performances.

The authors assert that all procedures contributing to this work comply with the ethical standards of the relevant national and institutional committees on human experimentation and with the Helsinki Declaration of 1975, as revised in 2013. All procedures involving human patients were approved by the Ethics Committee of Vaud (CER-VD, Switzerland). Written informed consent was obtained from all participants.

### SNP selection, genotyping and quality control

DNA was extracted from blood samples as described by the manufacturer protocol, using Flexigene DNA kit and QIAamp DNA Blood Mini QIAcube Kit (Qiagen AG, Switzerland). Genetic variants were determined by standard genotyping or imputation methods. Further details are available in the Supplementary Material. Only available SNPs of interest (i.e. previously associated with lipid levels in GWAS meta-analyses,^9,10,12–17^ not in linkage disequilibrium with each other, in Hardy–Weinberg equilibrium and available in GSA or imputed) were considered in the present study, corresponding to 124 SNPs for total cholesterol, 109 SNPs for LDL-C, 142 SNPs for HDL-C and 128 SNPs for triglycerides (more information in Supplementary Table 2). Information on SNPs that were not included in analyses is available in Supplementary Table 3.

### Statistical analyses

#### Selection of clinical and genetic variables of early lipid worsening by ≥5% in the discovery sample

In the present article, EWL was defined as early increase by ≥5% for total cholesterol, LDL-C or triglycerides, or early decrease by ≥5% for HDL-C, and the rest of the cohort was used as control.

In the discovery sample, clinical variables (i.e. age, gender, baseline body mass index (BMI), baseline lipid levels, diagnostic group, medication group, smoking status and psychiatric illness duration) were considered in logistic models on EWL through a stepwise model selection on the basis of Akaike information criterion (AIC), which minimises the distance between the fitted and the true model if such a model exists.^19^ Details on diagnostic and medication group categorisations are available in the Supplementary Material. It is noteworthy that even if some variables were not significantly influencing the distribution of the dependent variable (i.e. early lipid worsening), these were kept in the final model as advised by the AIC, to improve the general quality of the fitted model.

In the discovery sample, each SNP (i.e. 124 SNPs for total cholesterol, 109 SNPs for LDL-C, 142 SNPs for HDL-C and 128 SNPs for triglycerides), coded as having an additive effect, were considered as a predictor in logistic regressions on EWL using the least absolute shrinkage and selection operator (LASSO) method that shrinks regressions coefficients and automatically performs variable selection by setting some coefficients to zero, thus improving the predictive performance and introducing parsimony.^20^ PRS, which combined the contribution of above-mentioned SNPs and population-based estimates (Supplementary Table 2) into a single variable for each lipid phenotype (further described in a previous study^11^), were also included in above-mentioned logistic regressions.

### Predictive models of EWL

To assess the predictive value of previously selected clinical (using the AIC method) and genetic (using the LASSO method) factors on EWL, receiver operating characteristic (ROC) curve analyses were conducted to compare predictive powers of models including only clinical data with models containing both clinical and genetic data, using pROC and predictABEL packages in R.^21,22^ ROC analyses on EWL were first conducted in the discovery sample (*n* = 177), and then tested for replication in replication samples 1 (*n* = 176) and 2 (*n* = 86), considering regression models refitted in both above-mentioned samples. More details are available in the Supplementary Material.

### Predictive models of NOD in the combined psychiatric sample

NOD was defined by the new incidence of high total cholesterol (≥5 mmol/L), high LDL-C (≥3 mmol/L), high fasting triglycerides (≥2 mmol/L) and/or low HDL-C (≤1 mmol/L) levels,^23^ and/or the new prescription of a lipid-lowering agent during psychotropic treatment (median treatment duration of 100 days, interquartile range 40–365). Analyses of NOD were conducted in patients from the discovery and the replication samples who did not meet dyslipidaemia criterion at baseline (*n* = 203, 210, 290 and 225 for total cholesterol, LDL-C, HDL-C and triglycerides, respectively). Further information is detailed in the Supplementary Material. Genetic risk scores included SNPs selected in LASSO models fitted in the PsyMetab discovery sample, with corresponding estimates for each corresponding lipid phenotype (*n* = 17, 18, 19 and 16 SNPs for total cholesterol, LDL-C, HDL-C and triglycerides, respectively), to produce the PsyMetab genetic risk score (psyGRS). Survival analyses were used to compare NOD with psyGRS for the corresponding lipid phenotypes, using the survfit package of R.

### Analyses in the UK Biobank

Genetic risk scores considering SNPs selected in the discovery sample (i.e. psyGRS) were tested for association with lipid evolution in the UK Biobank, a large population-based cohort from the UK, with rich genotype and phenotype information.^24^ Two participant groups were defined. Participants who were taking at least one of the medications listed in Supplementary Table 1 were included in the ‘psychotropic treatment’ group, whereas participants not taking any medication listed in Supplementary Table 1 or any other medication defined as weight gain-inducing according to the SIDER database were considered as ‘controls’.^25^ It is noteworthy that the psyGRS included SNPs selected in LASSO models fitted in the PsyMetab discovery sample, with corresponding estimates for each corresponding lipid phenotype (*n* = 17, 18, 19 and 16 SNPs for total cholesterol, LDL-C, HDL-C and triglycerides, respectively; Supplementary Tables 4–7). Further details are available in the Supplementary Material.

All tests were two sided and *P*-values up to 0.05 were considered statistically significant. Statistical analyses were carried out using R, statistical software for Windows, version 4.1.1 (R Foundation for Statistical Computing, Vienna, Austria; see https://www.r-project.org).

## Results

### Demographic and clinical characteristics of the discovery sample

Supplementary Table 8 displays demographic and clinical characteristics of the discovery psychiatric sample. A total of 177 patients who did not receive any lipid-lowering agent during the first month of psychotropic treatment were included. The median age was 41 years (interquartile range 29–55), psychotic disorders were the most frequent diagnosis (25%) and quetiapine was the most frequently prescribed psychotropic drug (25%). During the first month of psychotropic treatment, 78 (44%), 72 (44%) and 62 (52%) patients developed early increase in total cholesterol, LDL-C and triglycerides levels, respectively, and 51 (29%) patients developed early decrease in HDL-C levels. Patients with early weight gain were more represented among patients with early total cholesterol and triglyceride increase, compared with others (18 *v.* 7%; *P* = 0.04 and 20 *v.* 5%; *P* = 0.04, respectively). The proportion of men was higher among patients with early triglyceride increase compared with others (55 *v.* 35%; *P* = 0.048).

### Predictive models

#### Models considering only clinical variables in the discovery sample

Low baseline lipid levels in total cholesterol, LDL-C and triglycerides, and high baseline HDL-C levels, were significant risk factors for early increase in total cholesterol (*P* = 0.002), LDL-C (*P* = 0.02) and triglycerides (*P* = 0.0006), and for early decrease in HDL-C (*P* = 0.04) (Supplementary Tables 4–7). Although not significantly associated with the dependent variable, low baseline BMI and smoking status improved fitted models on early worsening of total cholesterol and LDL-C. Men were more likely than women to have early HDL-C (*P* = 0.02) and triglyceride worsening (*P* = 0.002). In addition, compared with patients who started psychotropic medications with a low propensity to induce metabolic disturbances, patients who received drugs associated with a medium or high risk were more likely to develop early decrease in HDL-C (*P* = 0.01 and *P* = 0.005, respectively).

#### Models combining clinical and genetic variables in the discovery sample

Full information on SNPs considered in the present study is reported in Supplementary Table 2. LASSO models indicated that 17 (i.e. rs13114070T > C (*TMPRSS11E*), rs12588415G > A (*YLPM1*), rs2111485G > A (*unknown*), rs6557781C > T (*DMTN*), rs2287623A > G (*ABCB11*), rs964184C > G (*AP006216*.10; *ZPR1*), rs1077514T > C (*ASAP3*), rs4738684G > A (*unknown*), rs174554A > G (*FADS1*; *FADS2*; *MIR1908*), rs1997243A > G (*AC073957*.*15*; *C7orf50*; *GPR146*), rs1800961C > T (*HNF4A*), rs2758886G > A (*unknown*), rs12027135T > A (*TMEM57*), rs2618568A > C (*unknown*), rs10128711C > T (*SPTY2D1*; *SPTY2D1-AS1*), rs2954029A > T (*RP11-136O12.2*), rs10904908A > G (*VIM-AS1*)), 18 (i.e. rs2522061G > T (*AC116366.6* ; *C5orf56*; *Y_RNA*), rs7902274T > G (*RP11-564D11.3*), rs704G > A (*CTB-96E2.2; CTB-96E2.3; CTB-96E2.7; SARM1; SEBOX; TMEM199; VTN*), rs648324T > G (*PEX14*), rs7538216C > T (*unknown*), rs4773173A > G (*COL4A2*), rs3812945T > C (*SCAMP5*), rs28555129C > A (*OSGIN1*; *RP11-505K9.4*), rs826682A > C (*LIMS1*), rs14234A > G (*AC022201.5*; *FAM136A*; *SNRPG*), rs17404153G > T (*DNAJC13*), rs1030431G > A (*unknown*), rs174583C > T (*FADS2*), rs11136341A > G (*PLEC*), rs1800562G > A (*HFE*; *HIST1H2BB*), rs11648003A > G (*DHODH*), rs2920503C > T (*PPARG*), rs9987289G > A (*RP11-115J16.1*)), 19 (i.e. rs12529923C > T (*GSTA10P*; *GSTA11P*; *GSTA3*; *GSTA5*), rs17576323T > C (*AC007319.1*), rs11727676T > C (*HHIP*; *uc_338*), rs2683521G > A (*Metazoa_SRP; SP1*), rs10494363G > A (*MTMR11*; *OTUD7B*), rs10911505T > C (*unknown*), rs1519480T > C (*BDNF*; *BDNF-AS*), rs2373459T > C (*SPIC*), rs1126930G > C (*PRKAG1*; *RP11-386G11.5*), rs2280334C > T (*MEIS1*; *MEIS1-AS2*; *MEIS1-AS3*), rs7730898A > G (*RANBP17*), rs3173615C > G (*TMEM106B*), rs3822072G > A (*FAM13A*), rs970548A > C (*MARCH8*), rs7134594T > C (*MMAB*), rs11246602T > C (*OR4C46*), rs7134375C > A (*unknown*), rs11869286C > G (*STARD3*), rs4765127G > T (*CCDC92*; *FAM101A*; *RP11-214K3.18*; *RP11-214K3.25*; *ZNF664*)) and 16 (i.e. rs7519429A > C (*DNM3*; *PIGC*), rs4683438G > T (*RP11-372E1.4*), rs3851294G > A (*DSTYK*), rs1133400A > G (*INPP5A*), rs6430090G > A (*AC092484.1*), rs10199914A > G (*AC114788.1*), rs382534C > T (*SLC33A1*), rs12206516A > G (*unknown*), rs13266634C > T (*SLC30A8*), rs4738141A > G (*RP11-1102P16.1*), rs3843935C > T (*PRSS3*; *TRBV29OR9*-*2; UBE2R2-AS1*), rs2131925T > G (*DOCK7*), rs17585887C > T (*unknown*), rs1495741A > G (*unknown*), rs731839A > G (*PEPD*), rs72959041G > A (*RSPO3*)) individual SNPs were considered as important contributing factors in the prediction of early worsening of total cholesterol, LDL-C, HDL-C and triglyceride levels, respectively (Supplementary Tables 4–7). Of note, PRS (i.e. combining the contribution of all lipid level-associated SNPs weighted by their (external) population-based estimates) to EWL during the first month of treatment were not selected in predictive models.

**Fig. 1:**
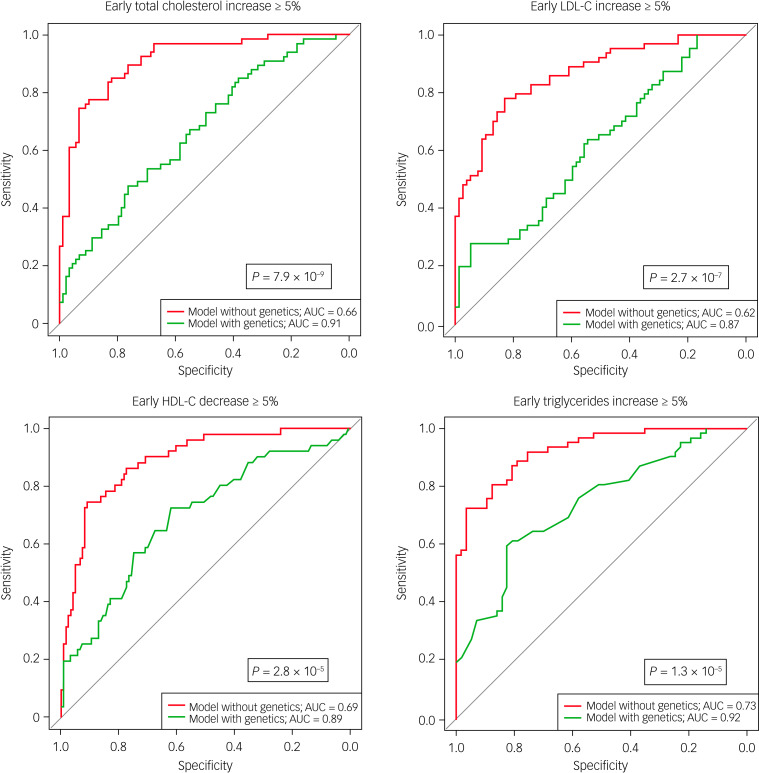
Receiver operating characteristic curves for early worsening of lipid levels in the discovery sample. Red curves correspond to models including clinical and genetics factors, whereas green curves include only clinical factors. Only fasting patients were included for triglyceride analyses. AUC, area under the curve; HDL-C, high-density lipoprotein cholesterol; LDL-C, low-density lipoprotein cholesterol.

Figure 1 and Table 1 show that in the discovery sample, compared with the area under the curve (AUC) from models restricted on clinical factors, the AUC from models including clinical and genetic factors were significantly improved for early increase in total cholesterol (from 0.66 to 0.91; *P* = 7.9 × 10^−9^), LDL-C (from 0.62 to 0.87; *P* = 2.7 × 10^−7^) and triglyceride (from 0.73 to 0.92; *P* = 1.3 × 10^−5^) levels, and early decrease in HDL-C levels (from 0.69 to 0.89; *P* = 2.8 × 10^−5^). Considerable increases in sensitivity, specificity, positive predictive value, negative predictive value and accuracy were also observed (Table 1). Likelihood ratio tests comparing models considering only clinical variables and models combining clinical and genetic parameters confirmed that adding genetic data improved the goodness of fit for EWL (*P* ≤ 4.1 × 10^−6^), which also indicated that the observed AUC improvements were not driven by the inclusion of a higher number of variables in models that considered genetic factors. Distribution of predicted risks for EWL were better discriminated in models combining clinical and genetic variables compared with models considering clinical factors only (Supplementary Fig. 2). Similarly, predictiveness curves, which also reflect the discriminatory power of prediction models, were better characterised for models including genetic variables compared with models restricted on clinical variables (Supplementary Fig. 3).

**Table 1:** Predictive power of models including only clinical variables and including clinical plus genetic variables on early worsening of lipid levels in the discovery sample

Dependent variable	Logistic model	Sensitivity, % (95% CI)	Specificity, % (95% CI)	PPV, % (95% CI)	NPV, % (95% CI)	Accuracy, % (95% CI)	AUC (95% CI)	*P*-value^c^	*P*-value Likelihood ratio test^d^
Total cholesterol ≥5%	Clinical^a^	0.63 (0.46–0.81)	0.64 (0.46–0.81)	0.57 (0.49–0.68)	0.7 (0.63–0.78)	0.63 (0.56–0.71)	0.66 (0.58–0.75)	**7.9 × 10^−9^**	**2.9 × 10^−10^**
	Clinical plus genetic^b^	0.85 (0.75–0.94)	0.85 (0.75–0.96)	0.82 (0.73–0.93)	0.88 (0.82–0.94)	0.85 (0.79–0.9)	0.91 (0.87–0.96)		
LDL-C ≥ 5%	Clinical^a^	0.64 (0.47–0.77)	0.57 (0.44–0.71)	0.55 (0.48–0.64)	0.65 (0.57–0.74)	0.6 (0.52–0.68)	0.62 (0.53–0.71)	**2.7 × 10^−7^**	**4.1 × 10^−6^**
	Clinical plus genetic^b^	0.8 (0.69–0.89)	0.83 (0.73–0.91)	0.79 (0.71–0.88)	0.83 (0.76–0.9)	0.82 (0.74–0.87)	0.87 (0.81–0.92)		
HDL-C ≥ 5%	Clinical^a^	0.69 (0.55–0.82)	0.66 (0.56–0.79)	0.46 (0.39–0.56)	0.84 (0.79–0.9)	0.67 (0.6–0.75)	0.69 (0.61–0.78)	**2.8 × 10^−5^**	**2.2 × 10^−6^**
	Clinical plus genetic^b^	0.82 (0.73–0.92)	0.85 (0.73–0.94)	0.69 (0.57–0.85)	0.92 (0.88–0.96)	0.84 (0.77–0.91)	0.89 (0.84–0.94)		
Triglycerides ≥5%	Clinical^a^	0.65 (0.53–0.82)	0.79 (0.58–0.89)	0.76 (0.66–0.87)	0.67 (0.6–0.77)	0.71 (0.64–0.79)	0.73 (0.65–0.83)	**1.3 × 10^−5^**	**3.9 × 10^−7^**
	Clinical plus genetic^b^	0.85 (0.74–0.95)	0.86 (0.75–0.96)	0.87 (0.79–0.96)	0.85 (0.76–0.94)	0.86 (0.8–0.92)	0.92 (0.88–0.97)		

PPV, positive predictive value; NPV, negative predictive value; AUC, area under the curve; LDL-C, low-density lipoprotein cholesterol; HDL-C, high-density lipoprotein cholesterol; AIC, Akaike information criterion; SNP, single nucleotide polymorphism; LASSO, least absolute shrinkage and selection operator. Significant *P*-values are indicated in bold.

a. Logistic model including clinical parameters selected using the AIC method.

b. Logistic model including clinical parameters selected using the AIC method plus SNPs selected using the LASSO method.

c. *P*-values of difference between the AUC of the model containing clinical factors and the model containing clinical plus genetic factors. Two thousand bootstraps were used for the analysis.

d. *P*-values of likelihood ratio tests to compare the model including only the clinical factors and the model containing clinical plus genetic factors. A significant *P*-value indicates an improved goodness of fit.

#### Validation in replication samples

Demographic and clinical characteristics of replication samples are shown in Supplementary Tables 9 and 10. Comparison of demographic and clinical factors between discovery and replication samples is reported in Supplementary Table 11 and further described in the Supplementary Material. Clinical and genetic variables selected in the discovery sample (i.e. listed in Supplementary Tables 4–7) were considered in replication samples to predict EWL. Estimates of each clinical and genetic factors calculated using logistic models on early worsening of total cholesterol, LDL-C, HDL-C and triglycerides levels are reported in Supplementary Tables 12–15 for replication sample 1 and in Supplementary Tables 16–19 for replication sample 2. In replication sample 1, adding genetic (i.e. SNPs and estimates from Supplementary Tables 12–15) to clinical factors significantly improved the prediction of early worsening of LDL-C and of HDL-C levels (with AUC increasing from 0.73 to 0.84 (*P* = 0.004) for LDL-C, and from 0.73 to 0.81 (*P* = 0.008) for HDL-C) (Table 2, Supplementary Fig. 4). In replication sample 2, prediction of early worsening of total cholesterol, LDL-C and triglycerides increased significantly when adding genetic (i.e. SNPs and estimates from Supplementary Tables 16–19) to clinical factors (AUC increasing from 0.65 to 0.85 (*P* = 0.002) for total cholesterol, from 0.66 to 0.90 (*P* = 0.0004) for LDL-C and from 0.67 to 0.82 (*P* = 0.04) for triglycerides) (Table 3, Supplementary Fig. 5).

**Table 2:** Predictive power of models including only clinical variables and including clinical plus genetic variables on early worsening of lipid levels in replication sample 1

Dependent variable	Logistic model	Sensitivity, % (95% CI)	Specificity, % (95% CI)	PPV, % (95% CI)	NPV, % (95% CI)	Accuracy, % (95% CI)	AUC (95% CI)	*P*-value^c^	*P*-value Likelihood ratio test^d^
Total cholesterol ≥5%	Clinical^a^	0.69 (0.57–0.87)	0.81 (0.61–0.9)	0.75 (0.64–0.85)	0.75 (0.69–0.85)	0.75 (0.68–0.82)	0.78 (0.70–0.85)	0.2	0.98
	Clinical plus genetic^b^	0.76 (0.63–0.88)	0.73 (0.62–0.87)	0.72 (0.64–0.82)	0.79 (0.71–0.87)	0.76 (0.69–0.82)	0.80 (0.73–0.87)		
LDL-C ≥ 5%	Clinical^a^	0.7 (0.57–0.83)	0.68 (0.54–0.8)	0.65 (0.57–0.74)	0.73 (0.66–0.82)	0.69 (0.62–0.76)	0.73 (0.65–0.82)	**0.004**	0.07
	Clinical plus genetic^b^	0.86 (0.75–0.94)	0.74 (0.64–0.84)	0.73 (0.66–0.81)	0.86 (0.78–0.94)	0.79 (0.73–0.86)	0.84 (0.77–0.90)		
HDL-C ≥ 5%	Clinical^a^	0.65 (0.53–0.79)	0.7 (0.56–0.83)	0.63 (0.54–0.73)	0.73 (0.66–0.8)	0.68 (0.61–0.75)	0.73 (0.65–0.80)	**0.008**	0.06
	Clinical plus genetic^b^	0.8 (0.65–0.91)	0.74 (0.62–0.88)	0.71 (0.63–0.82)	0.83 (0.75–0.91)	0.77 (0.71–0.83)	0.81 (0.75–0.88)		
Triglycerides ≥5%	Clinical^a^	0.77 (0.54–0.88)	0.57 (0.45–0.73)	0.62 (0.55–0.71)	0.71 (0.6–0.83)	0.66 (0.58–0.74)	0.68 (0.58–0.77)	0.08	0.88
	Clinical plus genetic^b^	0.75 (0.55–0.86)	0.65 (0.53–0.83)	0.67 (0.59–0.78)	0.73 (0.64–0.83)	0.7 (0.62–0.78)	0.74 (0.65–0.83)		

PPV, positive predictive value; NPV, negative predictive value; AUC, area under the curve; LDL-C, low-density lipoprotein cholesterol; HDL-C, high-density lipoprotein cholesterol; AIC, Akaike information criterion; SNP, single nucleotide polymorphism; LASSO, least absolute shrinkage and selection operator. Significant *P*-values are indicated in bold.

a. Logistic model conducted in the replication sample 1 including clinical factors selected using the AIC method in the discovery sample.

b. Logistic model conducted in the replication sample 1 including clinical factors selected using the AIC method plus SNPs selected using the LASSO method in the discovery sample.

c. *P*-values of difference between the AUC of the model containing clinical data and the model containing clinical and genetic data. Two thousand bootstraps were used for the analysis.

d. *P*-values of likelihood ratio tests to compare the model including only the clinical factors and the model containing clinical plus genetic factors. A significant *P*-value indicates an improved goodness of fit.

**Table 3:** Predictive power of models including only clinical variables and including clinical plus genetic variables on early worsening of lipid levels in replication sample 2

Dependent variable	Logistic model	Sensitivity, % (95% CI)	Specificity, % (95% CI)	PPV, % (95% CI)	NPV, % (95% CI)	Accuracy, % (95% CI)	AUC (95% CI)	*P*-value^c^	*P*-value Likelihood ratio test^d^
Total cholesterol ≥5%	Clinical^a^	0.69 (0.5–0.85)	0.7 (0.56–0.82)	0.54 (0.42–0.67)	0.81 (0.72–0.9)	0.68 (0.58–0.79)	0.65 (0.51–0.78)	**0.002**	0.16
	Clinical plus genetic^b^	0.85 (0.69–1)	0.76 (0.62–0.88)	0.65 (0.54–0.79)	0.91 (0.83–1)	0.79 (0.7–0.87)	0.85 (0.76–0.93)		
LDL-C ≥ 5%	Clinical^a^	0.64 (0.46–0.82)	0.74 (0.51–0.87)	0.63 (0.49–0.79)	0.73 (0.64–0.84)	0.69 (0.57–0.79)	0.66 (0.53–0.80)	**0.0004**	**0.006**
	Clinical plus genetic^b^	0.86 (0.71–0.96)	0.85 (0.72–0.97)	0.81 (0.69–0.96)	0.9 (0.81–0.97)	0.85 (0.78–0.93)	0.90 (0.84–0.97)		
HDL-C ≥ 5%	Clinical^a^	0.63 (0.43–0.83)	0.65 (0.44–0.85)	0.53 (0.43–0.7)	0.74 (0.65–0.85)	0.64 (0.54–0.76)	0.64 (0.51–0.77)	0.06	0.86
	Clinical plus genetic^b^	0.7 (0.5–0.9)	0.75 (0.52–0.96)	0.64 (0.5–0.9)	0.8 (0.72–0.9)	0.73 (0.62–0.85)	0.76 (0.64–0.87)		
Triglycerides ≥5%	Clinical^a^	0.63 (0.4–0.91)	0.7 (0.41–0.93)	0.72 (0.62–0.88)	0.59 (0.49–0.81)	0.66 (0.55–0.76)	0.67 (0.54–0.80)	**0.04**	0.36
	Clinical plus genetic^b^	0.77 (0.6–0.91)	0.81 (0.63–0.96)	0.84 (0.73–0.96)	0.73 (0.61–0.87)	0.79 (0.68–0.87)	0.82 (0.72–0.93)		

PPV, positive predictive value; NPV, negative predictive value; AUC, area under the curve; LDL-C, low-density lipoprotein cholesterol; HDL-C, high-density lipoprotein cholesterol; AIC, Akaike information criterion; SNP, single nucleotide polymorphism; LASSO, least absolute shrinkage and selection operator. Significant *P*-values are indicated in bold.

a. Logistic model conducted in the replication sample 2 including clinical factors selected using the AIC method in the discovery sample.

b. Logistic model conducted in the replication sample 2 including clinical factors selected using the AIC method plus SNPs selected using the LASSO method in the discovery sample.

c. *P*-values of difference between the AUC of the model containing clinical data and the model containing clinical and genetic data. Two thousand bootstraps were used for the analysis.

d. *P*-values of likelihood ratio tests to compare the model including only the clinical factors and the model containing clinical plus genetic factors. A significant *P*-value indicates an improved goodness of fit.

#### Prediction of NOD in the combined sample

In the combined sample consisting of patients from discovery and replication samples without baseline dyslipidaemia, 40%, 33%, 19% and 16% of patients developed total cholesterol, LDL-C, HDL-C and triglyceride NOD, respectively. Adding genetic (i.e. psyGRS) to clinical parameters significantly improved the prediction of new-onset hypercholesterolaemia (*P* = 0.04), new-onset LDL-hypercholesterolaemia (*P* = 0.002) and new-onset HDL-hypocholesterolaemia (*P* = 0.01), whereas a trend for improvement was observed for the prediction of new-onset hypertriglyceridaemia (*P* = 0.07) (Supplementary Table 20; Fig. 6). Predictive models including clinical and genetic factors on NOD for the four lipid traits provided clinically meaningful AUC (≥0.77) as well as high negative predictive values (0.83, 0.9, 0.95 and 0.95) for total cholesterol, LDL-C, HDL-C and triglyceride NOD, respectively, implying that most patients who were predicted not to develop NOD did not develop NOD during psychotropic treatment. As an example, Supplementary Figs 7–9 illustrate, for each lipid trait, the incidence of NOD in patients with fixed clinical parameters according to psyGRS groups, using different cut-offs.

Evaluation of the pharmacogenetic screening benefit is fully detailed in the Supplementary Material.

### Association between genetic risk scores and relative lipid difference in UK Biobank participants

Analyses conducted in the subset of UK Biobank participants taking a psychotropic treatment revealed a consistent but nominal association between psyGRS for triglycerides and a longitudinal increase of triglyceride levels (*P* = 0.03). Similar analysis in control participants yielded a non-significant association (*P* = 0.74). PsyGRS for the three remaining lipid phenotypes (i.e. total cholesterol, LDL-C and HDL-C) were not associated with relative differences of corresponding lipids, neither in ‘psychotropic treatment’ nor in ‘control’ UK Biobank participants (Supplementary Table 21).

## Discussion

The present study demonstrated that baseline clinical factors (i.e. before initiation of psychotropic treatment) combined with lipid-associated genetic variants are involved in the prediction of early lipid worsening and NOD in three psychiatric samples.

During the first month of psychotropic treatment, early worsening of total cholesterol, LDL-C, HDL-C and triglyceride levels was observed in 44%, 44%, 29% and 52% of patients, respectively. These proportions are globally consistent with those reported in our previous study including a similar number of patients, but with no genetic data.^7^ Of note, the prevalence of early decrease of HDL-C levels was lower than previously observed (42%),^7^ but this proportion was in agreement with those in replication samples 1 and 2 (43 and 38%, respectively).

Predictive models identified low baseline lipid levels of total cholesterol, LDL-C and triglycerides, and high baseline HDL-C levels, as significant risk factors for early worsening of the corresponding lipid levels. These findings are in accordance with our previous study reporting a lower proportion of baseline hyperlipidaemia in patients with EWL,^7^ as well as with other studies reporting low baseline BMI as a predictor of early weight gain,^26–29^ and may be explained by regression to the mean (i.e. low baseline lipid levels are expected to converge to the population mean during follow-up) and/or by the fact that low baseline lipid levels may be confounded by a short illness history and/or less prior psychotropic treatment exposure. Male gender was considered as an additional risk factor for early deterioration of HDL and triglycerides, consistent with a previous meta-analysis on predictors of metabolic dysregulation induced by antipsychotics.^3^ It is noteworthy that AIC models did not select illness duration as a predictive variable of early lipid disturbances.

In the discovery sample, adding genetic risk factors for lipids to baseline clinical parameters significantly improved the prediction of early worsening of total cholesterol, LDL-C, HDL-C and triglycerides levels during the first month of psychotropic treatment. The additive value of genetics to predict EWL was confirmed in the two replication samples for early worsening of LDL-C levels, and in one replication sample for early worsening of total cholesterol, HDL-C and triglycerides levels. Lack of improvement for early deterioration of total cholesterol in replication sample 1 may be explained by the relatively elevated and clinically informative predictive value (i.e. AUC = 0.78) of the model, which exclusively considered clinical factors.^30,31^

No PRS for any of the four lipid traits was considered as an important contributing factor in predictive models for EWL. One hypothesis to explain the poor predictive power of the PRS may be that the use of SNP estimates from the population-based sample could be not appropriate for the psychiatric population. Indeed, genetic variants can display greater influence on metabolic features in psychiatric cohorts compared with the general population, possibly explained by an intricate interaction between the mental disorder (or the medications used to treat it) and metabolic regulations,^32–34^ as well as a higher prevalence of metabolic abnormalities in the psychiatric population.^35^ Of note, when adding psyGRS (derived from SNP estimates calculated in the discovery sample) to clinical variables in replication samples, prediction of early lipid worsening was not improved. This can be explained by the fact that SNP estimates may be imprecise and/or unsuitable to other samples, because of the relatively low sample size of the discovery sample (*n* = 177). Future studies including a higher number of patients who start a psychotropic treatment would help to refine SNP estimates in this specific population.

Interestingly, this study demonstrated that the consideration of a reasonable number of SNPs (i.e. *n* ≤ 19) significantly improved predictive values of EWL, reaching AUC ≥ 0.8 in three psychiatric samples for each lipid phenotype (except for triglycerides in replication sample 1 (AUC = 0.74) and HDL-C in replication sample 2 (AUC = 0.76)). Predictive models combining clinical and genetic variables to predict early lipid worsening emphasised the additive value of genetics, displaying high predictive values (i.e. AUC ≥ 0.8) and high accuracy improvements (≥9% for early worsening of LDL-C and HDL-C in three samples). The considerable improvements of accuracy (i.e. ≥0.15) and AUC (i.e. ≥0.19) for predicting EWL when adding genetic factors in the discovery sample can be explained by the fact that predictive models specifically selected SNPs (i.e. *n* ≤ 19) that were best predicting EWL. When focusing on the contribution of each SNP on lipid-worsening outcomes, it appeared that estimate of each SNP was comparable or even higher than the estimate of one selected clinical factor (e.g. gender or smoking status), explaining how the addition of each selected SNP brought valuable additional information to the prediction of our outcomes. Of note, in replication sample 2, unexpectedly high estimates were observed for some SNPs (i.e. rs1800961C > T, rs1800562G > A, rs1126930G > C), which is explained by the low number of patients carrying the variant allele for the corresponding SNPs (i.e. minor allele frequencies of 0.02, 0.04 and 0.02, respectively). Sensitivity analyses showed that excluding these SNPs did not affect predictive values of outcomes of interest.

With regard to the incidence of NOD during psychotropic treatment, 40%, 33%, 19% and 16% of patients developed total cholesterol, LDL-C, HDL-C and triglyceride NOD, which is in accordance with NOD incidences reported in a previous study.^7^ The clinically meaningful predictive values of models considering clinical and genetic factors on NOD phenotypes observed for total cholesterol, LDL-C, HDL-C and triglycerides (AUC = 0.77, 0.81, 0.82 and 0.84, respectively; negative predictive value  = 0.83, 0.9, 0.95 and 0.95, respectively) validates the relevance of their consideration. Of note, NOD incidence varied depending on percentile groups of psyGRS (i.e. genetic risk score considering only the most relevant SNPs for EWL; *n* ≤ 19), which also further confirmed the added value of considering these selected genetic variants in the prediction of lipid phenotype deterioration under psychotropic treatment.

Although UK Biobank participants taking a psychotropic treatment were selected to reflect, as best as possible, PsyMetab participants, both samples varied considerably. For instance, in the UK Biobank, lipid values were measured independently of psychotropic treatment timeline. In addition, duration between both lipid measurements varied from 2 to 12 years in the UK Biobank sample, which is much longer than in PsyMetab (1 month). Despite these considerable differences, a consistent but nominal association between psyGRS and triglyceride relative difference was observed in UK Biobank ‘psychotropic treatment’ (but not ‘control’) participants. Hypotheses to explain the lack of association between psyGRS and relative difference of other lipid phenotypes (i.e. total cholesterol, HDL-C and LDL-C) in ‘psychotropic treatment’ and ‘control’ UK Biobank participants may reside in substantial design differences across PsyMetab and UK Biobank samples.

Of note, considering additional clinical variables (e.g. daily doses or plasma concentrations of psychotropic drugs) or genetic variants of enzymes involved in the metabolism of psychotropic drugs inducing worsening of lipid levels may potentially be of interest. However, since previous studies reported a small to moderate influence of doses of psychotropic drugs on weight gain,^36–39^ and because the association between psychotropic doses and/or plasma concentrations and worsening of lipid levels has never been assessed previously, these two variables were not included within the scope of present predictive analyses. In addition, multiple enzymes are involved in the metabolism of psychotropic drugs included in the present study (e.g. CYP1A2, CYP2C19, CYP2D6, CYP3A4 and CYP3A5). The influence of their genetic polymorphisms on lipid level worsening should be assessed in a second step, once (and if) future studies observe an association between lipid level worsening and psychotropic drug doses and/or plasma concentrations.

Findings of the present study should be considered with limitations. First, this study included only White patients and results are not transposable to other ethnicities. Second, most of the patients were not drug-naïve and would have probably already experienced lipid profile disturbances with previous psychotropic treatments. However, as in real-world clinical settings most psychiatric patients are not drug-naïve, these results may be relevant to the majority of patients who start a psychotropic medication inducing metabolic disturbances. Third, lifestyle modifications throughout psychotropic treatment (e.g. physical exercise or diet habits) or family history of atherosclerosis, which could have influenced lipid levels, were not available in this study and could not be taken into account. Fourth, SNP estimates calculated in the discovery sample are not appropriate for consideration in other samples, and future larger studies should be conducted to appraise SNP estimates to be integrated into clinically applicable predictive models. Strengths of the study include the fact that patients who were not adherent to the psychotropic medication were excluded from the study, which reduced the impact of possible false-negative patients (e.g. patients whose lipid levels remain stable because they do not adhere to the psychotropic drug). In addition, the longitudinal and naturalistic design of the study and the confirmation of our results in two replication samples represent major assets. Also, the present study was conducted in adherence with transparent reporting of a multivariable prediction model for individual prognosis or diagnosis + artificial intelligence statement guidance (Supplementary Material).^40^

In conclusion, we demonstrated that the consideration of baseline clinical variables combined with lipid-associated genetic risk factors could accurately predict early lipid worsening and dyslipidaemia development in patients who start a psychotropic treatment inducing metabolic disturbances. Future larger studies should be conducted to refine SNP estimates to be integrated into clinically applicable predictive models. Eventually, in the prospect of individualising psychotropic treatment, prospective validation studies should be implemented in clinical settings to evaluate the clinical utility of these predictive models, as well as to appraise their cost-effectiveness.

## Supporting information

Delacrétaz et al. supplementary material Delacrétaz et al. supplementary material

## Data Availability

The data are not publicly available owing to privacy and ethical considerations. The process for accessing PsyMetab data is described in the following link: https://www.chuv.ch/fr/psychiatrie/dp-home/recherche/centres-et-unites-de-recherche/centre-de-neurosciences-psychiatriques-cnp/unite-de-pharmacogenetique-et-de-psychopharmacologie-clinique-uppc/psymetab.
