# Supplementary material for: Lipid disturbances induced by psychotropic drugs: clinical and genetic predictors for early worsening of lipid levels and new-onset dyslipidaemia in Swiss psychiatric samples
Source: BJPsych Open. 2024 Dec 5;10(6):e227. doi: 10.1192/bjo.2024.757 (PMC11698187; doi:10.1192/bjo.2024.757)
Supplement: Delacrétaz et al. supplementary material [file S2056472424007579sup001.docx]

**METHODS**

**Psychiatric samples**

Patients were considered compliant when drug plasma concentrations were higher than 10% of the lower border of the recommended therapeutic range (1). Diagnostic groups were categorized according to the ICD-10 classification of mental and behavioural disorders diagnostic criteria as follows: organic mental disorders ([F00-F09]), psychotic disorders ([F20.0-F24.9] & [F28-F29]), schizoaffective disorders ([F25.0-F25.9]), bipolar disorders ([F30.0-F31.9]) and depressive disorders ([F32.00-F33.9]). Psychotropic drugs were categorized into three dyslipidemia risk groups, as follows: olanzapine, clozapine and valproate were associated with the highest risk, mirtazapine, lithium, risperidone, quetiapine were considered as conferring an intermediate risk, while aripiprazole, amisulpride, amitriptyline, brexpiprazole, carbamazepine, haloperidol, levomepromazine, lurasidone and zuclopenthixol were considered at lower risk (2, 3). Further description of the psychiatric samples was published elsewhere (4).

**SNP selection, genotyping and quality control**

DNA samples from patients from the discovery sample and the replication sample 1 were genotyped on GSA v2 with multiple disease option chip and processed on an iScan equipped platform (Illumina, San Diego, CA) at the iGE3 genomics platform of the University of Geneva (<http://www.ige3.unige.ch/genomics-platform.php>), whereas patients included in the replication sample 2 were processed similarly but genotyped on the third version of GSA (v3). All quality control and filtering steps were performed in PLINK (20). Ancestry was determined using *snpweights*, a software for inferring genome-wide ancestry using SNP weights precomputed from large external reference panels (5). For each participant, the software computed a respective percentage of European, East Asian, North American and East African ancestry. Using a threshold of 0.7 for East African, and 0.8 for all other ancestries, participants were attributed into one ethnic group. If no ancestry met this threshold, participants were defined as “mixed”. Only individuals from European ancestry were considered in the present study. Genotypes were called using data-driven clustering according to GenomeStudio recommendations, and subsequently exported in PLINK format (6, 7). Standard quality control filters were applied before imputation (8, 9). Finally, data was prepared for imputation according to Michigan Imputation Server guidelines and submitted to the server for imputation using HRC as a reference panel and European as the population (10, 11). Further quality control filters were applied post-imputation. KING kinship statistics were used to estimate relatedness. Using a kinship threshold of 0.0884 (as recommended by the KING authors), relatedness individuals were removed such that a maximal set of unrelated individuals were retained (12).

**Predictive models of EWL**

The area under the ROC curve (AUC) indicates how well model classifies a patient as a positive or a negative case (i.e. a patient with early lipid level worsening or not). An AUC of 0.5 would indicate a random test, whereas an AUC of 1 suggests an ideal test where all patients are correctly classified (13). Tests having an AUC of 0.75 are considered informative and useful enough (14). AUCs of the different models were compared using a bootstrap test as published previously (15). Median and confidence interval of accuracy (percentage of correctly classified cases among all patients), specificity, sensitivity, negative predictive value and positive predictive value were determined.

**Predictive models of NOD in the combined psychiatric sample**

ROC curve analyses on NOD were used to compare predictive powers of models including only clinical data with models considering both clinical and genetic data, using pROC and predictABEL packages of R (22, 23). Survival curves for NOD according to genetic risk score groups were fitted using the survival package of R. Predicted survivor functions were computed using Cox proportional hazard models considering mean clinical values for corresponding lipid phenotypes.

## Evaluation of pharmacogenetic screening benefit

The clinical value of pharmacogenetic testing could be assessed by calculating the number needed to genotype (NNG). NNG defines the number of patients who would need to be genotyped in order to prevent an EWL or NOD for one patient under psychotropic treatment (16). The calculation method is based on the inverse of the difference of accuracies between the model including genetic data and the model that considers clinical data without genetic data.

**Analyses in the UK Biobank (UKB)**

Only British, unrelated, consenting participants with two available prospective lipid measurements were included. Relative lipid difference was calculated for each participant as the second minus the first lipid value, divided by the first lipid value. Participants who reported to take cholesterol-lowering medications were removed from analyses (UKB data field: 6177). In both participant groups, psyGRS for TC, LDL-C, HDL-C and TG were tested for association with relative differences of corresponding lipids, adjusting for sex, age, age^2^ and duration between first and second lipid measurements.

**RESULTS**

**Validation in replication samples**

As compared to the discovery and replication sample 1, time elapsed between the initial and the first-month visit in replication sample 2 was slightly higher (34 days versus 31 days, p=0.002). Differences in diagnosis distribution were also observed, owing to the important proportion of missing diagnosis in the replication sample 2. Lower proportion of patients were treated with olanzapine, risperidone or valproate in the replication sample 2 as compared to other samples. Prevalence of early decrease of HDL levels was distributed differently across the three psychiatric samples (28.9%, 43.3% and 38%; p=0.02).

## Evaluation of the pharmacogenetic screening benefit

Accuracy (i.e., percentage of correctly classified cases) calculated in the discovery sample increased by 22% (from 63% to 85%) for early increase of TC, by 22% (from 60% to 82%) for early increase ≥5% in LDL-C, by 17% (from 67% to 84%) for early decrease of HDL-C and by 15% (from 71% to 86%) for early increase of TG. It corresponds to 5, 5, 7 and 6 patients that would be needed to be genotyped to detect one misclassified patient (when considering only clinical parameters) for early increase in TC, LDL-C, TG and early decrease in HDL-C, respectively. In the replication sample 1, accuracy improvements observed by adding genetic factors indicated that 10 and 12 patients would be needed to be genotyped to avoid one misclassified patient for early worsening of LDL-C and HDL-C levels, respectively (**Table 2**). Accuracy improvements observed in replication sample 2 (**Table 3**) indicated that 10, 7 and 8 patients would be needed to be genotyped to avoid one misclassified patient for early worsening of TC, LDL-C and TG levels, respectively. With regard to NOD prediction in the combined sample, the consideration of genetic factors increased accuracy by ≥5% for the four lipid phenotypes, corresponding to 20 patients that should be genotyped in order to detect one misclassified patient (**S20 Table**).

**S1 Table: Psychotropic drugs included in the metabolic follow-up recommendation.**

| **ANTIPSYCHOTICS** | | **ANTIDEPRESSANTS** | | **MOOD STABILIZERS** |
| --- | --- | --- | --- | --- |
|  |  |  |  |  |
| **Atypical  (second-generation)** | **Typical  (first-generation)** | **Tricyclic** | **Other** |  |
|  |  |  |  |  |
| Amisulpride | Chlorprothixene | Amitriptyline | Mirtazapine | Carbamazepine |
| Aripiprazole | Flupentixol | Clomipramine |  | Lithium |
| Asenapine | Haloperidol | Doxepine |  | Valproate |
| Clozapine | Levomepromazine | Imipramine |  |  |
| Lurasidone | Pipamperone | Nortriptyline |  |  |
| Olanzapine | Promazine | Opipramol |  |  |
| Paliperidone | Sulpiride | Trimipramine |  |  |
| Quetiapine | Tiapride |  |  |  |
| Risperidone | Zuclopenthixol |  |  |  |
| Sertindole |  |  |  |  |
|  |  |  |  |  |

According to international recommendations, a metabolic follow-up is ongoing since 2007 in the Department of Psychiatry at the Lausanne University Hospital, in which inpatients and outpatients are prospectively monitored when starting a pharmacological treatment known to have a potential risk to induce metabolic disturbances (i.e. drugs listed above). The list is based on psychotropic drugs available in Switzerland.

**S2 Table: Information on SNPs considered in the analyses**

| **Lipid trait** | **Source article** | **SNP** | **Analyzed proxy** | **Chr** | **Position  (GRCh37)** | **Ref allele** | **Alt allele** | **Effect  allele** | **Beta effect** | **MAF (CEU)** | **MAF** | **HW p-value** |
| --- | --- | --- | --- | --- | --- | --- | --- | --- | --- | --- | --- | --- |
| HDL-C | 1 | rs12748152 |  | 1 | 27138393 | C | T | C | 0.051 | 0.07 | 0.07 | 0.56 |
| HDL-C | 4 | rs12144063 |  | 1 | 28406047 | G | T | G | 0.004 | 0.29 | 0.29 | 0.81 |
| HDL-C | 2 | rs4660293 |  | 1 | 40028180 | A | G | A | 0.039 | 0.23 | 0.24 | 0.67 |
| HDL-C | 3 | rs55878063 |  | 1 | 61671909 | G | A | A | 0.04 | 0.08 | 0.05 | 0.33 |
| HDL-C | 4 | rs12740061 |  | 1 | 69407810 | C | T | T | 0.02 | 0.22 | 0.24 | 0.18 |
| HDL-C | 7 | rs2878349 |  | 1 | 107549245 | G | A | G | 0.0144 | 0.32 | 0.32 | 0.06 |
| HDL-C | 2 | rs6657811 |  | 1 | 109807283 | A | T | T | 0.057 | 0.11 | 0.14 | 0.79 |
| HDL-C | 3 | rs333947 |  | 1 | 110470764 | G | A | G | 0.028 | 0.13 | 0.14 | 0.15 |
| HDL-C | 7 | rs10494363 |  | 1 | 149909495 | G | A | A | 0.0324 | 0.09 | 0.07 | 0.56 |
| HDL-C | 1 | rs12145743 |  | 1 | 156700651 | T | G | G | 0.02 | 0.33 | 0.34 | 0.85 |
| HDL-C | 1 | rs1689800 |  | 1 | 182168885 | A | G | A | 0.034 | 0.35 | 0.41 | 0.30 |
| HDL-C | 7 | rs10911505 |  | 1 | 184049978 | T | C | T | 0.0131 | 0.34 | 0.32 | 0.09 |
| HDL-C | 7 | rs823114 |  | 1 | 205719532 | A | G | A | 0.0149 | 0.46 | 0.42 | 0.88 |
| HDL-C | 2 | rs4846914 |  | 1 | 230295691 | A | G | A | 0.048 | 0.40 | 0.42 | 0.82 |
| HDL-C | 7 | rs4850047 |  | 2 | 3634753 | C | T | T | 0.0195 | 0.16 | 0.15 | 0.52 |
| HDL-C | 2 | rs2678379 |  | 2 | 21226560 | G | A | A | 0.065 | 0.22 | 0.20 | 0.63 |
| HDL-C | 7 | rs17615494 |  | 2 | 58950363 | G | T | G | 0.0194 | 0.27 | 0.28 | 0.61 |
| HDL-C | 7 | rs2280334 |  | 2 | 66665146 | C | T | C | 0.0109 | 0.32 | 0.35 | 0.13 |
| HDL-C | 7 | rs6720034 |  | 2 | 111664756 | A | G | A | 0.0126 | 0.39 | 0.39 | 0.32 |
| HDL-C | 7 | rs13396091 |  | 2 | 146371961 | G | A | G | 0.0139 | 0.44 | 0.44 | 0.93 |
| HDL-C | 1 | rs12328675 |  | 2 | 165540800 | T | C | C | 0.045 | 0.16 | 0.14 | 0.93 |
| HDL-C | 3 | rs17576323 |  | 2 | 188003118 | T | C | C | 0.021 | 0.20 | 0.24 | 0.51 |
| HDL-C | 1 | rs1047891 |  | 2 | 211540507 | C | A | C | 0.027 | 0.30 | 0.34 | 0.37 |
| HDL-C | 2 | rs78058190 |  | 2 | 219699999 | G | A | G | 0.141 | 0.06 | 0.06 | 0.81 |
| HDL-C | 2 | rs2713536 |  | 2 | 227163642 | T | C | C | 0.038 | 0.38 | 0.39 | 0.75 |
| HDL-C | 2 | rs2606736 |  | 3 | 11400249 | T | C | C | 0.034 | 0.41 | 0.38 | 0.06 |
| HDL-C | 7 | rs2044753 |  | 3 | 24293001 | T | C | T | 0.0195 | 0.10 | 0.10 | 0.78 |
| HDL-C | 7 | rs6777217 |  | 3 | 36979042 | G | A | G | 0.0157 | 0.46 | 0.40 | 0.74 |
| HDL-C | 1 | rs2013208 |  | 3 | 50129399 | C | T | T | 0.025 | 0.49 | 0.49 | 0.15 |
| HDL-C | 1 | rs13326165 |  | 3 | 52532118 | G | A | A | 0.029 | 0.19 | 0.20 | 0.23 |
| HDL-C | 1 | rs6805251 |  | 3 | 119560606 | C | T | T | 0.02 | 0.38 | 0.42 | 0.15 |
| HDL-C | 3 | rs7614016 |  | 3 | 123070426 | G | A | G | 0.021 | 0.18 | 0.21 | 0.58 |
| HDL-C | 1 | rs13076253 |  | 3 | 131751775 | A | C | G | 0.028 | 0.15 | 0.15 | 0.11 |
| HDL-C | 2 | rs483465 |  | 3 | 136047977 | G | A | A | 0.045 | 0.25 | 0.27 | 0.72 |
| HDL-C | 7 | rs3773910 |  | 3 | 152171870 | C | G | G | 0.0141 | 0.25 | 0.26 | 0.13 |
| HDL-C | 7 | rs11248051 |  | 4 | 858332 | C | T | C | 0.0192 | 0.12 | 0.12 | 0.34 |
| HDL-C | 4 | rs34311866 |  | 4 | 951947 | T | C | T | 0.006 | 0.19 | 0.18 | 0.22 |
| HDL-C | 1 | rs10019888 |  | 4 | 26062990 | A | G | A | 0.027 | 0.18 | 0.18 | 0.49 |
| HDL-C | 1 | rs3822072 |  | 4 | 89741269 | G | A | G | 0.025 | 0.48 | 0.47 | 0.72 |
| HDL-C | 2 | rs13107325 |  | 4 | 103188709 | C | T | C | 0.12 | 0.08 | 0.07 | 0.60 |
| HDL-C | 3 | rs11727676 |  | 4 | 145659064 | T | C | T | 0.028 | 0.08 | 0.08 | 0.45 |
| HDL-C | 7 | rs17369400 |  | 4 | 154224048 | A | G | A | 0.0252 | 0.08 | 0.09 | 0.15 |
| HDL-C | 1 | rs6450176 |  | 5 | 53298025 | G | A | G | 0.025 | 0.28 | 0.27 | 0.57 |
| HDL-C | 7 | rs6881956 |  | 5 | 72926514 | G | A | G | 0.0177 | 0.28 | 0.27 | 0.48 |
| HDL-C | 7 | rs1045241 |  | 5 | 118729286 | C | T | T | 0.0135 | 0.26 | 0.31 | 0.11 |
| HDL-C | 7 | rs2434612 |  | 5 | 158022041 | A | G | A | 0.0226 | 0.21 | 0.20 | 0.86 |
| HDL-C | 7 | rs7730898 |  | 5 | 170459675 | A | G | G | 0.0182 | 0.27 | 0.26 | 0.37 |
| HDL-C | 2 | rs2814944 |  | 6 | 34552797 | G | A | G | 0.048 | 0.14 | 0.13 | 0.21 |
| HDL-C | 7 | rs4130023 |  | 6 | 41934514 | C | T | C | 0.0136 | 0.25 | 0.28 | 0.69 |
| HDL-C | 3 | rs10948059 |  | 6 | 42928461 | C | T | T | 0.02 | 0.49 | 0.47 | 0.35 |
| HDL-C | 1 | rs998584 |  | 6 | 43757896 | A | C | C | 0.026 | 0.50 | 0.47 | 0.38 |
| HDL-C | 3 | rs12529923 |  | 6 | 52736056 | C | T | C | 0.021 | 0.37 | 0.40 | 0.67 |
| HDL-C | 1 | rs1936800 |  | 6 | 127436064 | C | T | C | 0.02 | 0.48 | 0.48 | 0.35 |
| HDL-C | 7 | rs6925103 |  | 6 | 137076010 | T | C | C | 0.0121 | 0.47 | 0.48 | 0.30 |
| HDL-C | 1 | rs605066 |  | 6 | 139829666 | T | C | T | 0.028 | 0.43 | 0.41 | 0.35 |
| HDL-C | 3 | rs1281955 |  | 6 | 153459444 | T | A | A | 0.02 | 0.43 | 0.37 | 0.42 |
| HDL-C | 1 | rs702485 |  | 7 | 6449272 | A | G | G | 0.024 | 0.44 | 0.47 | 0.33 |
| HDL-C | 7 | rs3173615 |  | 7 | 12269417 | C | G | C | 0.0141 | 0.40 | 0.42 | 0.39 |
| HDL-C | 3 | rs2534596 |  | 7 | 38277792 | A | G | G | 0.02 | 0.34 | 0.37 | 0.42 |
| HDL-C | 1 | rs4917014 |  | 7 | 50305863 | T | G | G | 0.022 | 0.35 | 0.32 | 0.44 |
| HDL-C | 4 | rs77810251 |  | 7 | 121504149 | G | A | A | 0.052 | 0.12 | 0.14 | 0.18 |
| HDL-C | 2 | rs13241165 |  | 7 | 130432538 | A | T | T | 0.037 | 0.45 | 0.44 | 0.73 |
| HDL-C | 1 | rs17173637 |  | 7 | 150529449 | T | C | T | 0.036 | 0.09 | 0.09 | 0.90 |
| HDL-C | 7 | rs2936512 |  | 8 | 6599005 | C | T | C | 0.0149 | 0.31 | 0.31 | 0.10 |
| HDL-C | 2 | rs9987289 |  | 8 | 9183358 | G | A | G | 0.094 | 0.07 | 0.06 | 0.20 |
| HDL-C | 2 | rs12678919 |  | 8 | 19844222 | A | G | G | 0.167 | 0.13 | 0.12 | 0.95 |
| HDL-C | 3 | rs10091038 |  | 8 | 29360305 | C | A | A | 0.016 | 0.41 | 0.50 | 0.71 |
| HDL-C | 3 | rs10504474 |  | 8 | 71260460 | G | A | G | 0.0357 | 0.12 | 0.10 | 0.90 |
| HDL-C | 7 | rs10504474 |  | 8 | 71260460 | G | A | G | 0.0357 | 0.12 | 0.10 | 0.90 |
| HDL-C | 3 | rs13248499 |  | 8 | 121860589 | A | G | G | 0.025 | 0.37 | 0.41 | 0.85 |
| HDL-C | 2 | rs10808546 |  | 8 | 126495818 | C | T | T | 0.037 | 0.43 | 0.45 | 0.66 |
| HDL-C | 3 | rs10087900 |  | 8 | 144303418 | G | A | G | 0.027 | 0.45 | 0.45 | 0.89 |
| HDL-C | 2 | rs540885 |  | 9 | 15294596 | A | G | A | 0.055 | 0.13 | 0.13 | 0.27 |
| HDL-C | 2 | rs1883025 |  | 9 | 107664301 | C | T | C | 0.085 | 0.24 | 0.23 | 0.52 |
| HDL-C | 7 | rs4749779 |  | 10 | 8576206 | A | G | G | 0.0157 | 0.27 | 0.29 | 0.99 |
| HDL-C | 1 | rs970548 |  | 10 | 46013277 | A | C | C | 0.026 | 0.27 | 0.24 | 0.89 |
| HDL-C | 3 | rs4619105 |  | 10 | 115795236 | G | A | A | 0.026 | 0.11 | 0.11 | 0.96 |
| HDL-C | 3 | rs7079858 |  | 10 | 122859177 | G | T | G | 0.031 | 0.08 | 0.07 | 0.15 |
| HDL-C | 7 | rs2901286 |  | 10 | 122900623 | C | A | A | 0.0493 | 0.01 | 0.02 | 0.77 |
| HDL-C | 3 | rs7935422 | rs12802296 | 11 | 2963612 | C | T | C | 0.02 | 0.40 | 0.40 | 0.16 |
| HDL-C | 1 | rs2923084 |  | 11 | 10388782 | A | G | A | 0.026 | 0.17 | 0.17 | 0.63 |
| HDL-C | 7 | rs6486121 |  | 11 | 13355770 | T | C | C | 0.0122 | 0.34 | 0.40 | 0.94 |
| HDL-C | 7 | rs1519480 |  | 11 | 27675712 | T | C | T | 0.0139 | 0.30 | 0.31 | 0.10 |
| HDL-C | 2 | rs10838692 |  | 11 | 47345100 | T | C | C | 0.06 | 0.31 | 0.25 | 0.53 |
| HDL-C | 1 | rs11246602 |  | 11 | 51512090 | T | C | C | 0.034 | 0.13 | 0.09 | 0.56 |
| HDL-C | 3 | rs12575459 |  | 11 | 58162382 | G | A | A | 0.033 | 0.06 | 0.06 | 0.26 |
| HDL-C | 1 | rs174546 |  | 11 | 61569830 | C | T | C | 0.039 | 0.35 | 0.28 | 0.90 |
| HDL-C | 1 | rs499974 |  | 11 | 75455021 | C | A | C | 0.026 | 0.17 | 0.17 | 0.06 |
| HDL-C | 3 | rs10789752 |  | 11 | 109979945 | C | T | T | 0.018 | 0.31 | 0.32 | 0.15 |
| HDL-C | 2 | rs964184 |  | 11 | 116648917 | C | G | C | 0.102 | 0.16 | 0.15 | 0.81 |
| HDL-C | 1 | rs7941030 |  | 11 | 122522375 | T | C | C | 0.027 | 0.38 | 0.37 | 0.35 |
| HDL-C | 1 | rs7134375 |  | 12 | 20473758 | C | A | A | 0.021 | 0.40 | 0.43 | 0.63 |
| HDL-C | 7 | rs10842708 |  | 12 | 26474867 | A | G | A | 0.0174 | 0.25 | 0.23 | 0.57 |
| HDL-C | 7 | rs4551851 |  | 12 | 33459554 | A | G | G | 0.0158 | 0.47 | 0.45 | 0.78 |
| HDL-C | 7 | rs1126930 |  | 12 | 49399132 | G | C | G | 0.0358 | 0.02 | 0.02 | 0.68 |
| HDL-C | 1 | rs11613352 |  | 12 | 57792580 | C | T | T | 0.028 | 0.19 | 0.17 | 0.89 |
| HDL-C | 7 | rs2373459 |  | 12 | 101873956 | T | C | T | 0.0139 | 0.35 | 0.31 | 0.79 |
| HDL-C | 2 | rs7134594 |  | 12 | 110000193 | T | C | T | 0.036 | 0.46 | 0.46 | 0.30 |
| HDL-C | 7 | rs10507274 |  | 12 | 117160976 | T | C | C | 0.0247 | 0.07 | 0.05 | 0.34 |
| HDL-C | 1 | rs4759375 |  | 12 | 123796238 | C | T | T | 0.056 | 0.10 | 0.09 | 0.46 |
| HDL-C | 1 | rs4765127 |  | 12 | 124460167 | G | T | T | 0.032 | 0.37 | 0.38 | 0.87 |
| HDL-C | 2 | rs838880 |  | 12 | 125261593 | T | C | C | 0.056 | 0.31 | 0.33 | 0.24 |
| HDL-C | 3 | rs7148864 |  | 14 | 65119839 | A | G | G | 0.025 | 0.19 | 0.20 | 0.45 |
| HDL-C | 2 | rs4983559 |  | 14 | 105277209 | A | G | G | 0.039 | 0.39 | 0.39 | 0.60 |
| HDL-C | 2 | rs1532085 |  | 15 | 58683366 | G | A | A | 0.121 | 0.36 | 0.39 | 0.75 |
| HDL-C | 7 | rs2245477 |  | 15 | 61948435 | C | A | C | 0.0122 | 0.34 | 0.39 | 0.30 |
| HDL-C | 3 | rs1037116 |  | 15 | 102068904 | C | T | T | 0.02 | 0.21 | 0.18 | 0.99 |
| HDL-C | 1 | rs2652834 |  | 15 | 63396867 | G | A | G | 0.028 | 0.23 | 0.24 | 0.57 |
| HDL-C | 2 | rs7188861 |  | 16 | 11454650 | C | A | A | 0.044 | 0.19 | 0.19 | 0.81 |
| HDL-C | 1 | rs1121980 |  | 16 | 53809247 | G | A | G | 0.02 | 0.44 | 0.46 | 0.55 |
| HDL-C | 2 | rs17231506 |  | 16 | 56994528 | C | T | T | 0.243 | 0.29 | 0.29 | 0.50 |
| HDL-C | 2 | rs73591976 |  | 16 | 67811590 | C | A | A | 0.096 | 0.12 | 0.10 | 0.31 |
| HDL-C | 1 | rs2925979 |  | 16 | 81534790 | C | T | C | 0.035 | 0.29 | 0.30 | 0.60 |
| HDL-C | 7 | rs12938449 |  | 17 | 486821 | C | A | C | 0.0181 | 0.20 | 0.21 | 0.54 |
| HDL-C | 1 | rs11869286 |  | 17 | 37813856 | C | G | C | 0.032 | 0.33 | 0.33 | 0.46 |
| HDL-C | 5 | rs77697917 |  | 17 | 41840849 | C | T | C | 0.241 | 0.03 | 0.03 | 0.60 |
| HDL-C | 8 | rs12940636 |  | 17 | 53400110 | T | C | C | 0.025 | 0.34 | 0.34 | 0.46 |
| HDL-C | 1 | rs4148008 |  | 17 | 66875294 | C | G | C | 0.028 | 0.33 | 0.35 | 0.96 |
| HDL-C | 2 | rs12948394 |  | 17 | 76382791 | C | T | C | 0.034 | 0.47 | 0.49 | 0.88 |
| HDL-C | 7 | rs1788783 |  | 18 | 21161134 | C | T | T | 0.018 | 0.47 | 0.48 | 0.37 |
| HDL-C | 1 | rs12967135 |  | 18 | 57849023 | G | A | G | 0.026 | 0.24 | 0.24 | 0.89 |
| HDL-C | 7 | rs12975319 |  | 19 | 3414088 | G | A | G | 0.0135 | 0.30 | 0.32 | 0.91 |
| HDL-C | 1 | rs7255436 |  | 19 | 8433196 | A | C | A | 0.032 | 0.46 | 0.49 | 0.27 |
| HDL-C | 1 | rs737337 |  | 19 | 11347493 | T | C | T | 0.056 | 0.07 | 0.08 | 0.35 |
| HDL-C | 1 | rs731839 |  | 19 | 33899065 | A | G | A | 0.022 | 0.33 | 0.36 | 0.39 |
| HDL-C | 1 | rs17695224 |  | 19 | 52324216 | G | A | G | 0.029 | 0.25 | 0.31 | 0.21 |
| HDL-C | 2 | rs386000 |  | 19 | 54792761 | G | C | C | 0.043 | 0.19 | 0.17 | 0.20 |
| HDL-C | 7 | rs6120815 |  | 20 | 30184866 | T | C | C | 0.0149 | 0.16 | 0.15 | 0.09 |
| HDL-C | 3 | rs6059988 |  | 20 | 33278101 | T | C | T | 0.016 | 0.46 | 0.49 | 0.72 |
| HDL-C | 2 | rs1800961 |  | 20 | 43042364 | C | T | C | 0.149 | 0.04 | 0.02 | 0.72 |
| HDL-C | 2 | rs6073972 |  | 20 | 44590298 | C | G | C | 0.065 | 0.20 | 0.17 | 0.29 |
| HDL-C | 7 | rs2281279 |  | 20 | 46290250 | T | C | C | 0.0189 | 0.29 | 0.31 | 0.45 |
| HDL-C | 7 | rs856404 |  | 20 | 51263786 | A | G | G | 0.0125 | 0.33 | 0.31 | 0.99 |
| HDL-C | 7 | rs2834707 |  | 21 | 36343552 | C | T | C | 0.0152 | 0.33 | 0.35 | 0.26 |
| HDL-C | 3 | rs235374 |  | 21 | 46294986 | G | C | G | 0.017 | 0.45 | 0.43 | 0.70 |
| HDL-C | 2 | rs5754344 |  | 22 | 21963786 | A | G | A | 0.045 | 0.18 | 0.20 | 0.61 |
| LDL-C | 7 | rs648324 |  | 1 | 10556447 | T | G | T | 0.0146 | 0.29 | 0.29 | 0.39 |
| LDL-C | 7 | rs7538216 |  | 1 | 16509671 | C | T | C | 0.0131 | 0.39 | 0.40 | 0.32 |
| LDL-C | 7 | rs2992753 |  | 1 | 18808292 | A | C | C | 0.0126 | 0.39 | 0.36 | 0.52 |
| LDL-C | 1 | rs12027135 |  | 1 | 25775733 | T | A | T | 0.03 | 0.46 | 0.48 | 0.97 |
| LDL-C | 1 | rs12748152 |  | 1 | 27138393 | C | T | T | 0.05 | 0.07 | 0.07 | 0.56 |
| LDL-C | 2 | rs2479409 |  | 1 | 55504650 | A | G | G | 0.071 | 0.32 | 0.32 | 0.45 |
| LDL-C | 2 | rs3850634 |  | 1 | 63050598 | T | G | T | 0.042 | 0.30 | 0.28 | 0.26 |
| LDL-C | 3 | rs1730859 |  | 1 | 107617707 | A | G | G | 0.0195 | 0.32 | 0.33 | 0.21 |
| LDL-C | 7 | rs1730859 |  | 1 | 107617707 | A | G | G | 0.0195 | 0.32 | 0.33 | 0.21 |
| LDL-C | 2 | rs646776 |  | 1 | 109818530 | T | C | T | 0.146 | 0.21 | 0.23 | 0.90 |
| LDL-C | 1 | rs267733 |  | 1 | 150958836 | A | G | A | 0.033 | 0.14 | 0.15 | 0.76 |
| LDL-C | 1 | rs2642442 |  | 1 | 220973563 | T | C | T | 0.036 | 0.29 | 0.30 | 0.35 |
| LDL-C | 2 | rs514230 |  | 1 | 234858597 | T | A | T | 0.041 | 0.48 | 0.47 | 0.07 |
| LDL-C | 7 | rs73219351 |  | 2 | 17476540 | T | G | T | 0.0292 | 0.00 | 0.00 | 0.98 |
| LDL-C | 2 | rs1367117 |  | 2 | 21263900 | G | A | A | 0.108 | 0.30 | 0.29 | 0.39 |
| LDL-C | 2 | rs4299376 |  | 2 | 44072576 | T | G | G | 0.074 | 0.31 | 0.33 | 0.71 |
| LDL-C | 7 | rs12712955 |  | 2 | 46166321 | A | G | A | 0.0147 | 0.50 | 0.48 | 0.51 |
| LDL-C | 1 | rs2710642 |  | 2 | 63149557 | A | G | A | 0.024 | 0.37 | 0.40 | 0.36 |
| LDL-C | 7 | rs14234 |  | 2 | 70524142 | A | G | G | 0.0127 | 0.39 | 0.41 | 0.70 |
| LDL-C | 7 | rs826682 |  | 2 | 109232388 | A | C | C | 0.0149 | 0.15 | 0.14 | 0.93 |
| LDL-C | 1 | rs2030746 |  | 2 | 121309488 | C | T | T | 0.021 | 0.40 | 0.39 | 0.53 |
| LDL-C | 1 | rs1250229 |  | 2 | 216304384 | C | T | C | 0.024 | 0.23 | 0.24 | 0.66 |
| LDL-C | 2 | rs2920503 |  | 3 | 12324230 | C | T | T | 0.041 | 0.28 | 0.27 | 0.61 |
| LDL-C | 1 | rs7640978 |  | 3 | 32533010 | C | T | C | 0.039 | 0.11 | 0.12 | 0.86 |
| LDL-C | 1 | rs17404153 |  | 3 | 132163200 | G | T | G | 0.034 | 0.13 | 0.13 | 0.07 |
| LDL-C | 7 | rs10513551 |  | 3 | 160086055 | T | G | G | 0.0138 | 0.48 | 0.49 | 0.62 |
| LDL-C | 1 | rs6818397 |  | 4 | 3434885 | G | T | G | 0.022 | 0.41 | 0.42 | 0.12 |
| LDL-C | 7 | rs870992 |  | 5 | 52193237 | A | G | G | 0.0244 | 0.08 | 0.10 | 0.70 |
| LDL-C | 7 | rs3010276 |  | 5 | 72014569 | G | A | G | 0.0133 | 0.20 | 0.19 | 0.44 |
| LDL-C | 2 | rs12916 |  | 5 | 74656539 | T | C | C | 0.089 | 0.41 | 0.40 | 0.91 |
| LDL-C | 1 | rs4530754 |  | 5 | 122855416 | A | G | A | 0.028 | 0.42 | 0.42 | 0.32 |
| LDL-C | 3 | rs2522061 |  | 5 | 131804045 | G | T | G | 0.034 | 0.21 | 0.23 | 0.51 |
| LDL-C | 3 | rs4244029 |  | 5 | 141912841 | C | T | T | 0.027 | 0.15 | 0.17 | 0.08 |
| LDL-C | 7 | rs249756 |  | 5 | 141913503 | G | A | A | 0.0138 | 0.21 | 0.23 | 0.08 |
| LDL-C | 2 | rs6882076 |  | 5 | 156390297 | C | T | C | 0.042 | 0.35 | 0.41 | 0.55 |
| LDL-C | 2 | rs3757354 |  | 6 | 16127407 | C | T | C | 0.043 | 0.21 | 0.21 | 0.35 |
| LDL-C | 1 | rs1800562 |  | 6 | 26093141 | G | A | G | 0.062 | 0.04 | 0.04 | 0.44 |
| LDL-C | 2 | rs3177928 |  | 6 | 32412435 | G | A | A | 0.05 | 0.18 | 0.11 | 0.91 |
| LDL-C | 3 | rs17789218 |  | 6 | 100600097 | T | C | T | 0.023 | 0.24 | 0.26 | 0.25 |
| LDL-C | 1 | rs9488822 |  | 6 | 116312893 | A | T | T | 0.031 | 0.33 | 0.35 | 0.77 |
| LDL-C | 5 | rs186696265 |  | 6 | 161111700 | C | T | T | 0.304 | 0.01 | 0.01 | 0.85 |
| LDL-C | 7 | rs144787122 |  | 7 | 2296552 | A | G | A | 0.1055 | 0.00 | 0.00 | 0.96 |
| LDL-C | 2 | rs12670798 |  | 7 | 21607352 | T | C | C | 0.05 | 0.22 | 0.23 | 0.63 |
| LDL-C | 1 | rs4722551 |  | 7 | 25991826 | T | C | C | 0.039 | 0.17 | 0.18 | 0.71 |
| LDL-C | 2 | rs41279633 |  | 7 | 44580876 | G | T | T | 0.054 | 0.14 | 0.14 | 0.13 |
| LDL-C | 7 | rs1014283 |  | 7 | 87076587 | C | A | C | 0.0147 | 0.19 | 0.17 | 0.11 |
| LDL-C | 7 | rs221797 |  | 7 | 100285974 | C | A | A | 0.0224 | 0.11 | 0.08 | 0.57 |
| LDL-C | 7 | rs2712199 |  | 7 | 107140239 | A | G | G | 0.0153 | 0.34 | 0.34 | 0.67 |
| LDL-C | 3 | rs2912054 |  | 8 | 6601492 | T | G | T | 0.02 | 0.33 | 0.32 | 0.21 |
| LDL-C | 1 | rs9987289 |  | 8 | 9183358 | G | A | G | 0.071 | 0.07 | 0.06 | 0.20 |
| LDL-C | 7 | rs6557781 |  | 8 | 21937667 | C | T | C | 0.0187 | 0.15 | 0.13 | 0.16 |
| LDL-C | 1 | rs10102164 |  | 8 | 55421614 | G | A | A | 0.032 | 0.17 | 0.17 | 0.41 |
| LDL-C | 2 | rs1030431 |  | 8 | 59311697 | G | A | A | 0.037 | 0.34 | 0.39 | 0.96 |
| LDL-C | 2 | rs2954022 | rs2980860 | 8 | 126485337 | A | G | A | 0.054 | 0.45 | 0.45 | 0.92 |
| LDL-C | 2 | rs11136341 |  | 8 | 145043543 | A | G | G | 0.038 | 0.39 | 0.42 | 0.40 |
| LDL-C | 1 | rs3780181 |  | 9 | 2640759 | A | G | A | 0.044 | 0.05 | 0.08 | 0.11 |
| LDL-C | 2 | rs649129 |  | 9 | 136154304 | C | T | T | 0.064 | 0.21 | 0.24 | 0.93 |
| LDL-C | 3 | rs57176252 |  | 10 | 74662593 | C | A | C | 0.055 | 0.04 | 0.03 | 0.58 |
| LDL-C | 1 | rs2255141 |  | 10 | 113933886 | G | A | A | 0.03 | 0.31 | 0.34 | 0.22 |
| LDL-C | 3 | rs7902274 |  | 10 | 124657809 | T | G | T | 0.019 | 0.42 | 0.46 | 0.95 |
| LDL-C | 3 | rs11601507 |  | 11 | 5701074 | C | A | A | 0.051 | 0.07 | 0.06 | 0.70 |
| LDL-C | 2 | rs174583 |  | 11 | 61609750 | C | T | C | 0.063 | 0.36 | 0.31 | 0.81 |
| LDL-C | 7 | rs1981405 |  | 11 | 77976208 | C | T | C | 0.0181 | 0.13 | 0.11 | 0.82 |
| LDL-C | 2 | rs964184 |  | 11 | 116648917 | C | G | G | 0.08 | 0.16 | 0.15 | 0.81 |
| LDL-C | 8 | rs35882350 |  | 12 | 623129 | A | G | G | 0.033 | 0.25 | 0.25 | 0.46 |
| LDL-C | 3 | rs1907631 |  | 12 | 40606663 | T | C | C | 0.032 | 0.10 | 0.10 | 0.66 |
| LDL-C | 7 | rs1521516 |  | 12 | 51055708 | C | T | C | 0.0156 | 0.37 | 0.34 | 0.56 |
| LDL-C | 3 | rs7968419 |  | 12 | 109137726 | C | T | T | 0.02 | 0.28 | 0.28 | 0.24 |
| LDL-C | 1 | rs11065987 |  | 12 | 112072424 | A | G | A | 0.027 | 0.42 | 0.43 | 0.85 |
| LDL-C | 2 | rs1169314 |  | 12 | 121443116 | A | G | G | 0.037 | 0.32 | 0.32 | 0.27 |
| LDL-C | 1 | rs4942486 |  | 13 | 32953388 | C | T | T | 0.024 | 0.48 | 0.50 | 0.79 |
| LDL-C | 7 | rs4773173 |  | 13 | 111025118 | A | G | A | 0.0143 | 0.36 | 0.39 | 0.96 |
| LDL-C | 1 | rs8017377 |  | 14 | 24883887 | G | A | A | 0.03 | 0.48 | 0.49 | 0.36 |
| LDL-C | 3 | rs11620731 |  | 14 | 70817141 | C | T | C | 0.041 | 0.14 | 0.13 | 0.72 |
| LDL-C | 3 | rs17580 |  | 14 | 94847262 | T | A | A | 0.073 | 0.06 | 0.05 | 0.30 |
| LDL-C | 7 | rs3812945 |  | 15 | 75289722 | T | C | C | 0.0123 | 0.44 | 0.42 | 0.71 |
| LDL-C | 2 | rs247617 |  | 16 | 56990716 | C | A | C | 0.069 | 0.29 | 0.29 | 0.50 |
| LDL-C | 2 | rs11648003 |  | 16 | 72052348 | A | G | G | 0.067 | 0.23 | 0.23 | 0.78 |
| LDL-C | 3 | rs67890964 |  | 16 | 83979317 | T | C | T | 0.027 | 0.38 | 0.34 | 0.85 |
| LDL-C | 7 | rs28555129 |  | 16 | 83984776 | C | A | A | 0.0141 | 0.32 | 0.35 | 0.09 |
| LDL-C | 2 | rs314253 |  | 17 | 7091650 | T | C | T | 0.04 | 0.33 | 0.33 | 0.41 |
| LDL-C | 3 | rs704 |  | 17 | 26694861 | G | A | A | 0.018 | 0.45 | 0.49 | 0.88 |
| LDL-C | 2 | rs7206971 |  | 17 | 45425115 | G | A | A | 0.038 | 0.47 | 0.50 | 0.79 |
| LDL-C | 3 | rs2645492 |  | 17 | 57875554 | G | A | G | 0.029 | 0.18 | 0.20 | 0.77 |
| LDL-C | 1 | rs1801689 |  | 17 | 64210580 | A | C | C | 0.103 | 0.04 | 0.04 | 0.47 |
| LDL-C | 3 | rs73352129 | rs8076052 | 17 | 73779198 | A | C | C | 0.019 | 0.31 | 0.31 | 0.65 |
| LDL-C | 7 | rs329007 |  | 18 | 9522606 | A | G | A | 0.0154 | 0.21 | 0.23 | 0.93 |
| LDL-C | 2 | rs79588679 |  | 18 | 19907770 | C | T | C | 0.049 | 0.16 | 0.16 | 0.30 |
| LDL-C | 7 | rs941408 |  | 19 | 2814181 | C | T | T | 0.0136 | 0.29 | 0.30 | 0.84 |
| LDL-C | 2 | rs112374545 |  | 19 | 11188899 | C | T | C | 0.25 | 0.11 | 0.13 | 0.85 |
| LDL-C | 6 | rs72658867 |  | 19 | 11231203 | G | A | G | 0.253 | 0.01 | 0.00 | 0.94 |
| LDL-C | 2 | rs10401969 |  | 19 | 19407718 | T | C | T | 0.111 | 0.07 | 0.07 | 0.76 |
| LDL-C | 2 | rs1065853 | rs7412 | 19 | [45412079](https://www.ncbi.nlm.nih.gov/variation/view/?q=rs7412&filters=source:dbsnp&assm=GCF_000001405.25) | C | T | C | 0.603 | 0.06 | 0.06 | 0.77 |
| LDL-C | 2 | rs117492019 |  | 19 | 58681861 | G | T | G | 0.047 | 0.20 | 0.19 | 0.32 |
| LDL-C | 8 | rs3747910 |  | 20 | 5528518 | A | G | A | 0.028 | 0.21 | 0.19 | 0.68 |
| LDL-C | 1 | rs364585 |  | 20 | 12962718 | G | A | G | 0.025 | 0.36 | 0.35 | 0.45 |
| LDL-C | 2 | rs2618568 |  | 20 | 17843968 | A | C | C | 0.049 | 0.42 | 0.44 | 0.05 |
| LDL-C | 2 | rs2902941 |  | 20 | 39091514 | A | G | A | 0.022 | 0.29 | 0.33 | 0.11 |
| LDL-C | 1 | rs6029526 |  | 20 | 39672618 | T | A | A | 0.044 | 0.46 | 0.45 | 0.52 |
| LDL-C | 3 | rs17660708 |  | 21 | 33059831 | T | C | C | 0.0355 | 0.06 | 0.03 | 0.34 |
| LDL-C | 7 | rs17660708 |  | 21 | 33059831 | T | C | C | 0.0355 | 0.06 | 0.03 | 0.34 |
| LDL-C | 7 | rs2183573 |  | 21 | 40574305 | G | A | G | 0.0163 | 0.40 | 0.43 | 0.70 |
| LDL-C | 1 | rs5763662 |  | 22 | 30378703 | C | T | T | 0.077 | 0.03 | 0.01 | 0.79 |
| LDL-C | 7 | rs855791 |  | 22 | 37462936 | G | A | A | 0.0154 | 0.39 | 0.45 | 0.52 |
| LDL-C | 1 | rs4253776 |  | 22 | 46629479 | A | G | T | 0.031 | 0.12 | 0.15 | 0.14 |
| LDL-C | 7 | rs12171249 |  | 22 | 50878927 | G | A | G | 0.0135 | 0.30 | 0.29 | 0.20 |
| TC | 7 | rs648324 |  | 1 | 10556447 | T | G | T | 0.0139 | 0.29 | 0.29 | 0.39 |
| TC | 7 | rs6656611 |  | 1 | 16515805 | C | T | C | 0.0116 | 0.42 | 0.39 | 0.30 |
| TC | 1 | rs1077514 |  | 1 | 23766233 | T | C | T | 0.03 | 0.14 | 0.14 | 0.49 |
| TC | 1 | rs12027135 |  | 1 | 25775733 | T | A | T | 0.027 | 0.46 | 0.48 | 0.97 |
| TC | 7 | rs6699701 |  | 1 | 28298951 | C | T | C | 0.013 | 0.28 | 0.28 | 0.93 |
| TC | 2 | rs2479409 |  | 1 | 55504650 | A | G | G | 0.066 | 0.32 | 0.32 | 0.45 |
| TC | 2 | rs3850634 |  | 1 | 63050598 | T | G | T | 0.076 | 0.30 | 0.28 | 0.26 |
| TC | 2 | rs7515577 |  | 1 | 93009438 | A | C | A | 0.042 | 0.19 | 0.20 | 0.49 |
| TC | 2 | rs646776 |  | 1 | 109818530 | T | C | T | 0.12 | 0.21 | 0.23 | 0.90 |
| TC | 7 | rs17030613 |  | 1 | 113190807 | A | C | A | 0.0164 | 0.23 | 0.22 | 0.28 |
| TC | 7 | rs477992 |  | 1 | 120257576 | G | A | G | 0.0132 | 0.32 | 0.32 | 0.90 |
| TC | 3 | rs373579 | rs11810143 | 1 | 161480649 | A | G | G | 0.033 | 0.14 | 0.14 | 0.76 |
| TC | 3 | rs4651135 |  | 1 | 182970547 | G | A | G | 0.025 | 0.47 | 0.47 | 0.60 |
| TC | 1 | rs2642442 |  | 1 | 220973563 | T | C | T | 0.035 | 0.29 | 0.30 | 0.35 |
| TC | 2 | rs514230 |  | 1 | 234858597 | T | A | T | 0.048 | 0.48 | 0.47 | 0.07 |
| TC | 7 | rs1126627 |  | 1 | 236718620 | C | T | T | 0.0109 | 0.32 | 0.35 | 0.72 |
| TC | 7 | rs6761104 |  | 2 | 17930195 | G | A | G | 0.0175 | 0.11 | 0.11 | 0.19 |
| TC | 2 | rs1041968 |  | 2 | 21232804 | G | A | A | 0.095 | 0.44 | 0.46 | 0.42 |
| TC | 2 | rs1260326 |  | 2 | 27730940 | C | T | T | 0.045 | 0.41 | 0.44 | 0.53 |
| TC | 2 | rs4299376 |  | 2 | 44072576 | T | G | G | 0.069 | 0.31 | 0.33 | 0.71 |
| TC | 7 | rs12712955 |  | 2 | 46166321 | A | G | A | 0.0138 | 0.50 | 0.48 | 0.51 |
| TC | 3 | rs2706770 |  | 2 | 70471899 | A | G | G | 0.025 | 0.39 | 0.41 | 0.64 |
| TC | 7 | rs14234 |  | 2 | 70524142 | A | G | G | 0.014 | 0.39 | 0.41 | 0.70 |
| TC | 7 | rs13395354 |  | 2 | 111600519 | C | T | T | 0.0165 | 0.18 | 0.16 | 0.09 |
| TC | 3 | rs55709272 |  | 2 | 113867288 | T | C | T | 0.024 | 0.44 | 0.44 | 0.80 |
| TC | 1 | rs2030746 |  | 2 | 121309488 | C | T | T | 0.02 | 0.40 | 0.39 | 0.53 |
| TC | 1 | rs7570971 |  | 2 | 135837906 | A | C | A | 0.03 | 0.49 | 0.45 | 0.40 |
| TC | 7 | rs2111485 |  | 2 | 163110536 | G | A | A | 0.0156 | 0.40 | 0.43 | 0.40 |
| TC | 1 | rs2287623 |  | 2 | 169830155 | A | G | G | 0.027 | 0.40 | 0.39 | 0.78 |
| TC | 2 | rs115400054 |  | 2 | 203676105 | C | T | C | 0.054 | 0.12 | 0.13 | 0.18 |
| TC | 2 | rs1699337 | rs709149 | 3 | 12450354 | G | A | G | 0.043 | 0.38 | 0.38 | 0.29 |
| TC | 1 | rs7640978 |  | 3 | 32533010 | C | T | C | 0.038 | 0.11 | 0.12 | 0.86 |
| TC | 1 | rs13315871 |  | 3 | 58381287 | G | A | G | 0.036 | 0.09 | 0.09 | 0.22 |
| TC | 7 | rs9870322 |  | 3 | 64706499 | T | C | T | 0.0113 | 0.29 | 0.34 | 0.78 |
| TC | 7 | rs1470121 |  | 3 | 142625778 | G | A | G | 0.0171 | 0.39 | 0.39 | 0.48 |
| TC | 7 | rs721023 |  | 3 | 160223172 | A | G | G | 0.0203 | 0.45 | 0.49 | 0.88 |
| TC | 1 | rs6818397 |  | 4 | 3434885 | G | T | G | 0.025 | 0.41 | 0.42 | 0.12 |
| TC | 7 | rs278981 |  | 4 | 40428010 | C | T | C | 0.0149 | 0.22 | 0.23 | 0.97 |
| TC | 3 | rs13114070 |  | 4 | 69349893 | T | C | T | 0.028 | 0.30 | 0.26 | 0.37 |
| TC | 7 | rs870992 |  | 5 | 52193237 | A | G | G | 0.0236 | 0.08 | 0.10 | 0.70 |
| TC | 7 | rs3010276 |  | 5 | 72014569 | G | A | G | 0.0135 | 0.20 | 0.19 | 0.44 |
| TC | 2 | rs12916 |  | 5 | 74656539 | T | C | C | 0.082 | 0.41 | 0.40 | 0.91 |
| TC | 1 | rs4530754 |  | 5 | 122855416 | A | G | A | 0.023 | 0.42 | 0.42 | 0.32 |
| TC | 2 | rs6882076 |  | 5 | 156390297 | C | T | C | 0.051 | 0.35 | 0.41 | 0.55 |
| TC | 1 | rs3757354 |  | 6 | 16127407 | C | T | C | 0.035 | 0.21 | 0.21 | 0.35 |
| TC | 3 | rs6456350 |  | 6 | 20405539 | G | A | G | 0.019 | 0.35 | 0.30 | 0.69 |
| TC | 1 | rs1800562 |  | 6 | 26093141 | G | A | G | 0.056 | 0.04 | 0.04 | 0.44 |
| TC | 5 | rs6457374 |  | 6 | 31272261 | T | C | T | 0.062 | 0.20 | 0.18 | 0.71 |
| TC | 2 | rs3177928 |  | 6 | 32412435 | G | A | A | 0.055 | 0.18 | 0.11 | 0.91 |
| TC | 1 | rs2814982 |  | 6 | 34546560 | C | T | C | 0.044 | 0.10 | 0.07 | 0.95 |
| TC | 7 | rs913499 |  | 6 | 37038432 | G | A | A | 0.0147 | 0.44 | 0.49 | 0.80 |
| TC | 1 | rs2758886 |  | 6 | 39250837 | G | A | A | 0.023 | 0.29 | 0.29 | 0.67 |
| TC | 7 | rs1326543 |  | 6 | 53497222 | A | G | G | 0.0135 | 0.24 | 0.25 | 0.84 |
| TC | 1 | rs9488822 |  | 6 | 116312893 | A | T | T | 0.034 | 0.33 | 0.35 | 0.77 |
| TC | 3 | rs9371220 |  | 6 | 151858598 | C | T | T | 0.027 | 0.13 | 0.11 | 0.21 |
| TC | 2 | rs12208357 |  | 6 | 160543148 | C | T | T | 0.092 | 0.06 | 0.08 | 0.99 |
| TC | 5 | rs186696265 |  | 6 | 161111700 | C | T | T | 0.278 | 0.01 | 0.01 | 0.85 |
| TC | 1 | rs1997243 |  | 7 | 1083777 | A | G | G | 0.033 | 0.12 | 0.17 | 0.44 |
| TC | 1 | rs12670798 |  | 7 | 21607352 | T | C | C | 0.036 | 0.22 | 0.23 | 0.63 |
| TC | 1 | rs4722551 |  | 7 | 25991826 | T | C | C | 0.029 | 0.17 | 0.18 | 0.71 |
| TC | 2 | rs2072183 |  | 7 | 44579180 | G | C | C | 0.041 | 0.23 | 0.22 | 0.45 |
| TC | 7 | rs1014283 |  | 7 | 87076587 | C | A | C | 0.0135 | 0.19 | 0.17 | 0.11 |
| TC | 7 | rs445 |  | 7 | 92408370 | C | T | T | 0.0159 | 0.11 | 0.14 | 0.36 |
| TC | 3 | rs314311 |  | 7 | 100422481 | T | G | G | 0.023 | 0.21 | 0.19 | 0.41 |
| TC | 2 | rs9987289 |  | 8 | 9183358 | G | A | G | 0.097 | 0.07 | 0.06 | 0.20 |
| TC | 1 | rs1495741 |  | 8 | 18272881 | A | G | G | 0.032 | 0.24 | 0.20 | 0.81 |
| TC | 7 | rs6557781 |  | 8 | 21937667 | C | T | C | 0.0195 | 0.15 | 0.13 | 0.16 |
| TC | 1 | rs10102164 |  | 8 | 55421614 | G | A | A | 0.03 | 0.17 | 0.17 | 0.41 |
| TC | 2 | rs4738684 |  | 8 | 59393273 | G | A | A | 0.041 | 0.37 | 0.39 | 0.50 |
| TC | 7 | rs7013120 |  | 8 | 74881674 | G | A | G | 0.0125 | 0.27 | 0.25 | 0.49 |
| TC | 2 | rs2954022 | rs2954029 | 8 | 126490972 | A | T | A | 0.063 | 0.45 | 0.45 | 0.90 |
| TC | 1 | rs11136341 |  | 8 | 145043543 | A | G | G | 0.038 | 0.39 | 0.42 | 0.40 |
| TC | 1 | rs3780181 |  | 9 | 2640759 | A | G | A | 0.044 | 0.05 | 0.08 | 0.11 |
| TC | 1 | rs581080 |  | 9 | 15305378 | C | G | C | 0.038 | 0.18 | 0.21 | 0.13 |
| TC | 7 | rs10738607 |  | 9 | 22088094 | A | G | A | 0.0167 | 0.49 | 0.48 | 0.97 |
| TC | 2 | rs1883025 |  | 9 | 107664301 | C | T | C | 0.068 | 0.24 | 0.23 | 0.52 |
| TC | 3 | rs74551598 |  | 9 | 117177566 | A | C | A | 0.03 | 0.49 | 0.25 | 0.16 |
| TC | 2 | rs507666 |  | 9 | 136149399 | G | A | A | 0.067 | 0.20 | 0.21 | 0.96 |
| TC | 1 | rs10904908 |  | 10 | 17260290 | A | G | G | 0.025 | 0.45 | 0.41 | 0.22 |
| TC | 1 | rs970548 |  | 10 | 46013277 | A | C | C | 0.025 | 0.27 | 0.24 | 0.89 |
| TC | 3 | rs12784396 |  | 10 | 102027407 | C | T | T | 0.02 | 0.43 | 0.44 | 0.91 |
| TC | 2 | rs2255141 |  | 10 | 113933886 | G | A | A | 0.036 | 0.31 | 0.34 | 0.22 |
| TC | 1 | rs10128711 |  | 11 | 18632984 | C | T | C | 0.031 | 0.28 | 0.23 | 0.79 |
| TC | 2 | rs174554 |  | 11 | 61579463 | A | G | A | 0.062 | 0.34 | 0.28 | 0.90 |
| TC | 7 | rs2511158 |  | 11 | 77973980 | C | T | C | 0.0226 | 0.18 | 0.15 | 0.78 |
| TC | 2 | rs964184 |  | 11 | 116648917 | C | G | G | 0.118 | 0.16 | 0.15 | 0.81 |
| TC | 1 | rs11603023 |  | 11 | 118486067 | C | T | T | 0.022 | 0.44 | 0.40 | 0.58 |
| TC | 2 | rs7128198 |  | 11 | 122526601 | C | T | T | 0.036 | 0.37 | 0.36 | 0.40 |
| TC | 8 | rs35882350 |  | 12 | 623129 | A | G | G | 0.028 | 0.25 | 0.25 | 0.46 |
| TC | 1 | rs4883201 |  | 12 | 9082581 | A | G | A | 0.035 | 0.11 | 0.10 | 0.29 |
| TC | 7 | rs12320328 |  | 12 | 25408464 | A | G | A | 0.0189 | 0.09 | 0.08 | 0.45 |
| TC | 3 | rs7955221 |  | 12 | 100850750 | A | C | A | 0.02 | 0.49 | 0.43 | 0.83 |
| TC | 1 | rs11065987 |  | 12 | 112072424 | A | G | A | 0.031 | 0.42 | 0.43 | 0.85 |
| TC | 2 | rs1169288 |  | 12 | 121416650 | A | C | C | 0.037 | 0.34 | 0.33 | 0.28 |
| TC | 7 | rs17532301 |  | 13 | 41609047 | G | A | G | 0.0304 | 0.08 | 0.08 | 0.33 |
| TC | 7 | rs4942859 |  | 13 | 50198158 | A | G | G | 0.014 | 0.43 | 0.42 | 0.13 |
| TC | 7 | rs4773173 |  | 13 | 111025118 | A | G | A | 0.0161 | 0.36 | 0.39 | 0.96 |
| TC | 3 | rs12588415 |  | 14 | 75278211 | G | A | G | 0.018 | 0.47 | 0.48 | 0.35 |
| TC | 3 | rs72731954 |  | 15 | 57442759 | A | G | G | 0.042 | 0.07 | 0.09 | 0.50 |
| TC | 2 | rs1532085 |  | 15 | 58683366 | G | A | A | 0.049 | 0.36 | 0.39 | 0.75 |
| TC | 1 | rs3764261 |  | 16 | 56993324 | C | A | A | 0.05 | 0.29 | 0.29 | 0.50 |
| TC | 2 | rs11648003 |  | 16 | 72052348 | A | G | G | 0.07 | 0.23 | 0.23 | 0.78 |
| TC | 2 | rs314253 |  | 17 | 7091650 | T | C | T | 0.037 | 0.33 | 0.33 | 0.41 |
| TC | 7 | rs7214248 |  | 17 | 28574177 | G | A | G | 0.0139 | 0.34 | 0.29 | 0.67 |
| TC | 3 | rs2854322 |  | 17 | 29699416 | T | C | T | 0.02 | 0.28 | 0.25 | 0.59 |
| TC | 2 | rs7206971 |  | 17 | 45425115 | G | A | A | 0.043 | 0.47 | 0.50 | 0.79 |
| TC | 3 | rs328996 |  | 18 | 9526184 | C | T | C | 0.021 | 0.21 | 0.23 | 0.93 |
| TC | 3 | rs941408 |  | 19 | 2814181 | C | T | T | 0.021 | 0.29 | 0.30 | 0.84 |
| TC | 2 | rs112374545 |  | 19 | 11188899 | C | T | C | 0.217 | 0.11 | 0.13 | 0.85 |
| TC | 2 | rs10401969 |  | 19 | 19407718 | T | C | T | 0.123 | 0.07 | 0.07 | 0.76 |
| TC | 2 | rs7412 |  | 19 | 45412079 | C | T | C | 0.413 | 0.06 | 0.06 | 0.77 |
| TC | 1 | rs492602 |  | 19 | 49206417 | A | G | G | 0.031 | 0.44 | 0.44 | 0.54 |
| TC | 8 | rs3747910 |  | 20 | 5528518 | A | G | A | 0.038 | 0.21 | 0.19 | 0.68 |
| TC | 2 | rs2618568 |  | 20 | 17843968 | A | C | C | 0.044 | 0.42 | 0.44 | 0.05 |
| TC | 7 | rs6058893 |  | 20 | 31392777 | C | T | T | 0.0133 | 0.37 | 0.34 | 0.96 |
| TC | 2 | rs2277862 |  | 20 | 34152782 | C | T | C | 0.052 | 0.15 | 0.18 | 0.74 |
| TC | 1 | rs6029526 |  | 20 | 39672618 | T | A | A | 0.04 | 0.46 | 0.45 | 0.52 |
| TC | 1 | rs1800961 |  | 20 | 43042364 | C | T | C | 0.106 | 0.04 | 0.02 | 0.72 |
| TC | 7 | rs2183573 |  | 21 | 40574305 | G | A | G | 0.0193 | 0.40 | 0.43 | 0.70 |
| TC | 1 | rs138777 |  | 22 | 35711098 | G | A | A | 0.021 | 0.33 | 0.37 | 0.51 |
| TC | 1 | rs4253772 |  | 22 | 46627603 | C | T | T | 0.032 | 0.12 | 0.14 | 0.29 |
| TG | 7 | rs55738118 |  | 1 | 11838451 | C | T | C | 0.0189 | 0.10 | 0.07 | 0.40 |
| TG | 1 | rs12748152 |  | 1 | 27138393 | C | T | T | 0.037 | 0.07 | 0.07 | 0.56 |
| TG | 7 | rs1278530 |  | 1 | 50889255 | G | A | G | 0.0174 | 0.46 | 0.48 | 0.18 |
| TG | 3 | rs144432213 | rs12068606 | 1 | 51351281 | C | T | C | 0.039 | 0.10 | 0.10 | 0.38 |
| TG | 2 | rs2131925 |  | 1 | 63025942 | T | G | T | 0.074 | 0.31 | 0.28 | 0.42 |
| TG | 7 | rs2613503 |  | 1 | 72839774 | A | C | A | 0.0184 | 0.18 | 0.16 | 0.80 |
| TG | 3 | rs6671166 |  | 1 | 154093825 | G | A | A | 0.018 | 0.34 | 0.37 | 0.86 |
| TG | 3 | rs7519429 |  | 1 | 172349246 | A | C | A | 0.025 | 0.37 | 0.37 | 0.25 |
| TG | 7 | rs2821231 |  | 1 | 203518382 | C | T | T | 0.0137 | 0.49 | 0.47 | 0.24 |
| TG | 7 | rs3851294 |  | 1 | 205130413 | G | A | G | 0.0267 | 0.11 | 0.07 | 0.85 |
| TG | 2 | rs340839 |  | 1 | 214161820 | G | A | A | 0.039 | 0.48 | 0.45 | 0.10 |
| TG | 3 | rs2791547 |  | 1 | 219659668 | T | A | T | 0.025 | 0.38 | 0.39 | 0.62 |
| TG | 2 | rs10864728 |  | 1 | 230304914 | G | A | A | 0.052 | 0.39 | 0.41 | 0.88 |
| TG | 2 | rs4665710 |  | 2 | 21221035 | C | A | C | 0.082 | 0.22 | 0.20 | 0.63 |
| TG | 2 | rs1260326 |  | 2 | 27730940 | C | T | T | 0.123 | 0.41 | 0.44 | 0.53 |
| TG | 7 | rs1861410 |  | 2 | 58933591 | T | C | C | 0.015 | 0.43 | 0.42 | 0.79 |
| TG | 2 | rs2540948 |  | 2 | 65284623 | T | C | T | 0.036 | 0.36 | 0.38 | 0.65 |
| TG | 3 | rs2280334 |  | 2 | 66665146 | C | T | T | 0.017 | 0.32 | 0.35 | 0.13 |
| TG | 7 | rs1519104 |  | 2 | 66680892 | A | G | G | 0.0153 | 0.30 | 0.34 | 0.05 |
| TG | 3 | rs77004761 |  | 2 | 111868691 | T | A | T | 0.039 | 0.12 | 0.11 | 0.91 |
| TG | 7 | rs6430090 |  | 2 | 146347459 | G | A | A | 0.0182 | 0.40 | 0.40 | 0.74 |
| TG | 1 | rs2972146 |  | 2 | 227100698 | T | G | T | 0.028 | 0.37 | 0.38 | 0.57 |
| TG | 7 | rs10199914 |  | 2 | 239896861 | A | G | G | 0.0114 | 0.36 | 0.37 | 1.00 |
| TG | 3 | rs6792725 |  | 3 | 24520283 | G | A | A | 0.018 | 0.33 | 0.30 | 0.33 |
| TG | 7 | rs6777217 |  | 3 | 36979042 | G | A | A | 0.0113 | 0.46 | 0.40 | 0.74 |
| TG | 1 | rs645040 |  | 3 | 135926622 | T | G | T | 0.029 | 0.23 | 0.23 | 0.93 |
| TG | 3 | rs4683438 |  | 3 | 142652559 | G | T | G | 0.018 | 0.36 | 0.36 | 0.61 |
| TG | 7 | rs2354167 |  | 3 | 142657205 | G | T | T | 0.0154 | 0.46 | 0.50 | 0.79 |
| TG | 7 | rs382534 |  | 3 | 155547274 | C | T | T | 0.0133 | 0.26 | 0.28 | 0.95 |
| TG | 3 | rs78086267 |  | 3 | 156915089 | A | G | A | 0.032 | 0.15 | 0.18 | 0.61 |
| TG | 7 | rs11720145 |  | 3 | 170739663 | G | A | A | 0.0196 | 0.14 | 0.14 | 0.33 |
| TG | 7 | rs17600346 |  | 3 | 172223982 | T | C | C | 0.0535 | 0.03 | 0.04 | 0.47 |
| TG | 7 | rs1564282 |  | 4 | 852313 | C | T | T | 0.0227 | 0.12 | 0.12 | 0.15 |
| TG | 4 | rs34311866 |  | 4 | 951947 | T | C |  | 0.006 | 0.19 | 0.18 | 0.22 |
| TG | 2 | rs6831256 |  | 4 | 3473139 | A | G | G | 0.037 | 0.40 | 0.42 | 0.42 |
| TG | 2 | rs2035403 |  | 4 | 88018991 | A | G | G | 0.039 | 0.38 | 0.41 | 0.99 |
| TG | 3 | rs2322549 |  | 4 | 143317027 | G | A | A | 0.018 | 0.38 | 0.41 | 0.21 |
| TG | 1 | rs9686661 |  | 5 | 55861786 | C | T | T | 0.038 | 0.19 | 0.18 | 0.38 |
| TG | 3 | rs1045241 |  | 5 | 118729286 | C | T | C | 0.0155 | 0.26 | 0.31 | 0.11 |
| TG | 7 | rs1045241 |  | 5 | 118729286 | C | T | C | 0.0155 | 0.26 | 0.31 | 0.11 |
| TG | 1 | rs6882076 |  | 5 | 156390297 | C | T | C | 0.029 | 0.35 | 0.41 | 0.55 |
| TG | 7 | rs2914228 |  | 5 | 158007165 | C | T | T | 0.0252 | 0.24 | 0.24 | 0.67 |
| TG | 7 | rs12206516 |  | 6 | 20506815 | A | G | A | 0.015 | 0.20 | 0.20 | 0.61 |
| TG | 2 | rs419132 |  | 6 | 32210799 | A | G | G | 0.056 | 0.27 | 0.39 | 0.95 |
| TG | 7 | rs2395655 |  | 6 | 36645696 | A | G | A | 0.0125 | 0.38 | 0.48 | 0.58 |
| TG | 2 | rs1358980 |  | 6 | 43764551 | C | T | T | 0.039 | 0.50 | 0.47 | 0.43 |
| TG | 7 | rs12208493 |  | 6 | 86662267 | T | C | C | 0.0148 | 0.29 | 0.32 | 0.78 |
| TG | 2 | rs72959041 |  | 6 | 127454893 | G | A | A | 0.075 | 0.05 | 0.05 | 0.12 |
| TG | 2 | rs17585887 |  | 6 | 139835498 | C | T | T | 0.039 | 0.43 | 0.41 | 0.30 |
| TG | 2 | rs7759633 |  | 6 | 161070990 | G | A | G | 0.051 | 0.13 | 0.13 | 0.44 |
| TG | 7 | rs4709741 |  | 6 | 164092291 | C | A | C | 0.0218 | 0.09 | 0.08 | 0.71 |
| TG | 7 | rs3173615 |  | 7 | 12269417 | C | G | G | 0.0128 | 0.40 | 0.42 | 0.39 |
| TG | 7 | rs38246 |  | 7 | 15941283 | C | T | T | 0.0147 | 0.28 | 0.28 | 0.84 |
| TG | 1 | rs4719841 |  | 7 | 25997536 | A | G | C | 0.023 | 0.39 | 0.45 | 0.99 |
| TG | 2 | rs9638182 |  | 7 | 72999105 | T | G | T | 0.1 | 0.19 | 0.18 | 0.32 |
| TG | 2 | rs2255811 |  | 7 | 112722196 | A | G | G | 0.041 | 0.27 | 0.27 | 0.66 |
| TG | 1 | rs38855 |  | 7 | 116358044 | A | G | A | 0.019 | 0.48 | 0.47 | 0.24 |
| TG | 1 | rs11776767 |  | 8 | 10683929 | G | C | C | 0.022 | 0.40 | 0.40 | 0.09 |
| TG | 1 | rs1495741 |  | 8 | 18272881 | A | G | G | 0.04 | 0.24 | 0.20 | 0.81 |
| TG | 2 | rs12678919 |  | 8 | 19844222 | A | G | A | 0.194 | 0.13 | 0.12 | 0.95 |
| TG | 7 | rs746011 |  | 8 | 22457804 | C | T | T | 0.02 | 0.33 | 0.36 | 0.75 |
| TG | 4 | rs10101067 |  | 8 | 72407374 | G | C | C | 0.014 | 0.06 | 0.05 | 0.29 |
| TG | 7 | rs4738141 |  | 8 | 72469742 | A | G | G | 0.0125 | 0.25 | 0.24 | 0.55 |
| TG | 7 | rs13266634 |  | 8 | 118184783 | C | T | C | 0.0141 | 0.28 | 0.27 | 0.24 |
| TG | 2 | rs2954029 |  | 8 | 126490972 | A | T | A | 0.082 | 0.45 | 0.47 | 0.90 |
| TG | 7 | rs3843935 |  | 9 | 33787871 | C | T | T | 0.0152 | 0.37 | 0.37 | 0.86 |
| TG | 7 | rs1982151 |  | 9 | 86617265 | G | A | G | 0.0116 | 0.28 | 0.31 | 0.45 |
| TG | 1 | rs1832007 |  | 10 | 5254847 | A | G | A | 0.033 | 0.12 | 0.13 | 0.60 |
| TG | 2 | rs10761731 |  | 10 | 65027610 | A | T | A | 0.034 | 0.43 | 0.43 | 0.46 |
| TG | 7 | rs2298117 |  | 10 | 70346740 | C | T | T | 0.0118 | 0.43 | 0.43 | 0.15 |
| TG | 1 | rs2068888 |  | 10 | 94839642 | G | A | G | 0.024 | 0.49 | 0.50 | 0.26 |
| TG | 7 | rs17779355 |  | 10 | 103928374 | G | A | G | 0.039 | 0.07 | 0.05 | 0.74 |
| TG | 7 | rs963059 |  | 10 | 112097073 | T | C | C | 0.0164 | 0.21 | 0.20 | 0.61 |
| TG | 7 | rs1133400 |  | 10 | 134459388 | A | G | G | 0.0178 | 0.21 | 0.23 | 0.24 |
| TG | 3 | rs2896635 |  | 11 | 13359745 | A | T | A | 0.019 | 0.30 | 0.34 | 0.45 |
| TG | 7 | rs1519480 |  | 11 | 27675712 | T | C | C | 0.0195 | 0.30 | 0.31 | 0.10 |
| TG | 3 | rs331446 |  | 11 | 36446712 | C | T | T | 0.02 | 0.22 | 0.22 | 0.84 |
| TG | 2 | rs174546 |  | 11 | 61569830 | C | T | T | 0.053 | 0.35 | 0.28 | 0.90 |
| TG | 3 | rs11231698 |  | 11 | 63877163 | C | T | T | 0.051 | 0.07 | 0.05 | 0.97 |
| TG | 7 | rs10793310 |  | 11 | 78099014 | T | G | T | 0.022 | 0.18 | 0.16 | 0.57 |
| TG | 7 | rs1079596 |  | 11 | 113296619 | C | T | T | 0.0148 | 0.15 | 0.12 | 0.28 |
| TG | 2 | rs964184 |  | 11 | 116648917 | C | G | G | 0.244 | 0.16 | 0.15 | 0.81 |
| TG | 3 | rs4149056 |  | 12 | 21331549 | T | C | C | 0.024 | 0.16 | 0.14 | 0.18 |
| TG | 7 | rs718314 |  | 12 | 26453283 | A | G | G | 0.0184 | 0.24 | 0.23 | 0.52 |
| TG | 7 | rs7316454 |  | 12 | 46200396 | G | T | T | 0.0254 | 0.20 | 0.21 | 0.93 |
| TG | 3 | rs2657880 |  | 12 | 56863770 | G | C | C | 0.023 | 0.21 | 0.24 | 1.00 |
| TG | 2 | rs61352607 |  | 12 | 57839173 | G | T | G | 0.038 | 0.19 | 0.17 | 0.65 |
| TG | 3 | rs10778215 |  | 12 | 103537266 | A | T | T | 0.016 | 0.49 | 0.41 | 0.71 |
| TG | 1 | rs4765127 |  | 12 | 124460167 | G | T | G | 0.029 | 0.37 | 0.38 | 0.87 |
| TG | 3 | rs2812208 |  | 13 | 50707087 | G | C | G | 0.075 | 0.03 | 0.02 | 0.77 |
| TG | 7 | rs2298058 |  | 13 | 95248566 | C | T | T | 0.0273 | 0.32 | 0.34 | 0.15 |
| TG | 3 | rs7333748 |  | 13 | 111018729 | C | G | C | 0.016 | 0.43 | 0.46 | 0.54 |
| TG | 3 | rs12431415 |  | 14 | 77503443 | G | A | A | 0.018 | 0.31 | 0.30 | 0.85 |
| TG | 7 | rs7176058 |  | 15 | 39464167 | A | G | A | 0.0145 | 0.16 | 0.13 | 0.90 |
| TG | 3 | rs1531140 |  | 15 | 42125234 | C | T | T | 0.018 | 0.36 | 0.31 | 0.16 |
| TG | 1 | rs2412710 |  | 15 | 42683787 | G | A | A | 0.099 | 0.03 | 0.02 | 0.70 |
| TG | 1 | rs2929282 |  | 15 | 44245931 | A | T | T | 0.072 | 0.05 | 0.05 | 0.34 |
| TG | 2 | rs1077835 |  | 15 | 58723426 | A | G | G | 0.059 | 0.21 | 0.24 | 0.47 |
| TG | 3 | rs4776342 |  | 15 | 67418391 | A | G | G | 0.024 | 0.21 | 0.24 | 0.22 |
| TG | 7 | rs730180 |  | 15 | 73007893 | A | G | A | 0.0129 | 0.20 | 0.27 | 0.99 |
| TG | 7 | rs11247287 |  | 15 | 101905533 | T | C | C | 0.0144 | 0.31 | 0.32 | 0.49 |
| TG | 1 | rs3198697 |  | 16 | 15129940 | C | T | C | 0.02 | 0.40 | 0.41 | 0.85 |
| TG | 1 | rs9930333 |  | 16 | 53799977 | T | G | A | 0.021 | 0.44 | 0.46 | 0.42 |
| TG | 1 | rs3764261 |  | 16 | 56993324 | C | A | C | 0.04 | 0.29 | 0.29 | 0.50 |
| TG | 7 | rs871290 |  | 16 | 85707367 | G | C | C | 0.015 | 0.33 | 0.31 | 0.75 |
| TG | 1 | rs8077889 |  | 17 | 41878166 | A | C | C | 0.025 | 0.25 | 0.23 | 0.38 |
| TG | 7 | rs1051424 |  | 17 | 58024324 | A | G | G | 0.0206 | 0.15 | 0.18 | 0.61 |
| TG | 7 | rs9948087 |  | 18 | 268992 | C | A | C | 0.0157 | 0.28 | 0.25 | 0.10 |
| TG | 7 | rs1788783 |  | 18 | 21161134 | C | T | C | 0.0158 | 0.47 | 0.48 | 0.37 |
| TG | 7 | rs12454712 |  | 18 | 60845884 | T | C | T | 0.0115 | 0.39 | 0.38 | 0.35 |
| TG | 7 | rs10853981 |  | 19 | 4965064 | G | A | A | 0.0177 | 0.35 | 0.32 | 0.18 |
| TG | 1 | rs7248104 |  | 19 | 7224431 | G | A | G | 0.022 | 0.42 | 0.43 | 0.47 |
| TG | 5 | rs116843064 |  | 19 | 8429323 | G | A | G | 0.087 | 0.03 | 0.02 | 0.70 |
| TG | 2 | rs10401969 |  | 19 | 19407718 | T | C | T | 0.12 | 0.07 | 0.07 | 0.76 |
| TG | 1 | rs731839 |  | 19 | 33899065 | A | G | G | 0.022 | 0.33 | 0.36 | 0.39 |
| TG | 7 | rs2018519 |  | 19 | 35559787 | T | C | C | 0.0241 | 0.17 | 0.17 | 0.23 |
| TG | 2 | rs439401 |  | 19 | 45414451 | C | T | C | 0.073 | 0.38 | 0.35 | 0.26 |
| TG | 3 | rs59774409 |  | 19 | 50016748 | C | T | T | 0.04 | 0.09 | 0.08 | 0.25 |
| TG | 7 | rs6088793 |  | 20 | 30211597 | T | C | T | 0.0136 | 0.16 | 0.16 | 0.06 |
| TG | 2 | rs4810479 |  | 20 | 44545048 | T | C | C | 0.052 | 0.29 | 0.24 | 0.42 |
| TG | 3 | rs6066141 |  | 20 | 45597546 | T | C | T | 0.021 | 0.26 | 0.29 | 0.81 |
| TG | 7 | rs1884507 |  | 20 | 51012164 | A | C | A | 0.0246 | 0.08 | 0.08 | 0.96 |
| TG | 3 | rs6025606 |  | 20 | 56098733 | C | T | C | 0.02 | 0.34 | 0.36 | 0.75 |
| TG | 7 | rs2836878 |  | 21 | 40465534 | G | A | A | 0.0134 | 0.26 | 0.27 | 0.27 |
| TG | 3 | rs5752792 |  | 22 | 29153759 | T | C | C | 0.022 | 0.19 | 0.20 | 0.31 |
| TG | 1 | rs5756931 |  | 22 | 38546033 | T | C | T | 0.02 | 0.39 | 0.38 | 0.95 |

SNPs are sorted by lipid trait, chromosome and genomic position.

Abbreviation: Alt allele: alternative allele, CEU: Caucasian European, Chr: chromosome, HDL-C: high-density lipoprotein cholesterol, HW: Hardy-Weinberg, LDL-C: low-density lipoprotein cholesterol, MAF: minor allele frequency calculated in the present study, MAF (CEU): reported minor allele frequency in the Caucasian European population, Ref allele: reference allele, SNP: single nucleotide polymorphism, TC: total cholesterol, TG: triglycerides.

Source articles:

1: Global Lipids Genetics C, et al. Discovery and refinement of loci associated with lipid levels. Nature genetics. 2013;45(11):1274-83.

2: Surakka I, et al. The impact of low-frequency and rare variants on lipid levels. Nature genetics. 2015;47(6):589-97.

3: Hoffmann TJ, et al. A large electronic-health-record-based genome-wide study of serum lipids. Nature genetics. 2018;50(3):401-13.

4: Bentley AR et al. Multi-ancestry genome-wide gene-smoking interaction study of 387,272 individuals identifies new loci associated with serum lipids. Nature genetics. 2019;51(4):636-48.

5: van Leeuwen EM et al. Meta-analysis of 49 549 individuals imputed with the 1000 Genomes Project reveals an exonic damaging variant in ANGPTL4 determining fasting TG levels. Journal of medical genetics. 2016;53(7):441-9.

6: Walter K, et al. The UK10K project identifies rare variants in health and disease. Nature. 2015;526(7571):82-90.

7: Klarin D, et al. Genetics of blood lipids among ~300,000 multi-ethnic participants of the Million Veteran Program. Nature genetics. 2018;50(11):1514-23.

8: Hu Y, et al. Minority-centric meta-analyses of blood lipid levels identify novel loci in the Population Architecture using Genomics and Epidemiology (PAGE) study. PLoS genetics. 2020;16(3):e1008684

**S3 Table: SNPs selected from the literature but not included in analyses**

| **Lipid trait** | **Source article** | **SNP** | **Reason for non-inclusion** |
| --- | --- | --- | --- |
| TC | 3 | rs56394279 | In LD with another included SNP |
| TC | 3 | rs306890 | Not available in GSA |
| TC | 3 | rs202246180 | Not available in GSA |
| TC | 1 | rs17526895 | in HW disequilibrium |
| TC | 1 | rs11220462 | in HW disequilibrium |
| TC | 2 | rs7239867 | in HW disequilibrium |
| LDL-C | 3 | rs826681 | In LD with another included SNP |
| LDL-C | 3 | rs72285796 | Not available in GSA |
| LDL-C | 1 | rs10490626 | in HW disequilibrium |
| LDL-C | 1 | rs1564348 | in HW disequilibrium |
| LDL-C | 2 | rs11220462 | in HW disequilibrium |
| LDL-C | 7 | rs892161 | in HW disequilibrium |
| LDL-C | 6 | rs12006849 | in HW disequilibrium |
| HDL-C | 3 | rs11048456 | In LD with another included SNP |
| HDL-C | 3 | rs138331350 | Not available in GSA |
| HDL-C | 3 | rs147879509 | Not available in GSA |
| HDL-C | 3 | rs200441621 | Not available in GSA |
| HDL-C | 3 | rs201330646 | Not available in GSA |
| HDL-C | 3 | rs145882729 | Not available in GSA |
| HDL-C | 3 | rs10198423 | Not available in GSA |
| HDL-C | 3 | rs1651274 | In LD with another included SNP |
| HDL-C | 3 | rs62362194 | Not available in GSA |
| HDL-C | 3 | rs58752567 | Not available in GSA |
| HDL-C | 2 | rs116569761 | Not available in GSA |
| HDL-C | 2 | rs77147124 | Not available in GSA |
| HDL-C | 7 | rs41278045 | Not available in GSA |
| HDL-C | 7 | rs139271800 | Not available in GSA |
| HDL-C | 4 | rs10937241 | in HW disequilibrium |
| HDL-C | 1 | rs2602836 | in HW disequilibrium |
| HDL-C | 1 | rs4142995 | in HW disequilibrium |
| HDL-C | 3 | rs112545201 | in HW disequilibrium |
| HDL-C | 3 | rs77403571 | in HW disequilibrium |
| HDL-C | 2 | rs10438978 | in HW disequilibrium |
| HDL-C | 3 | rs2683521 | in HW disequilibrium |
| TG | 3 | rs2958557 | Not available in GSA |
| TG | 3 | rs181937009 | Not available in GSA |
| TG | 3 | rs6695980 | In LD with another included SNP |
| TG | 3 | rs3859931 | Not available in GSA |
| TG | 3 | rs9556404 | In LD with another included SNP |
| TG | 3 | rs74664507 | Not available in GSA |
| TG | 7 | rs41278045 | Not available in GSA |
| TG | 1 | rs11649653 | in HW disequilibrium |
| TG | 3 | rs2299247 | in HW disequilibrium |
| TG | 7 | rs2245586 | in HW disequilibrium |

SNPs are sorted by lipid trait, chromosome and genomic position.

Abbreviation: HDL-C: high-density lipoprotein cholesterol, HW: Hardy-Weinberg, LDL-C: low-density lipoprotein cholesterol, SNP: single nucleotide polymorphism, TC: total cholesterol, TG: triglycerides.

Source articles:

1: Global Lipids Genetics C, et al. Discovery and refinement of loci associated with lipid levels. Nature genetics. 2013;45(11):1274-83.

2: Surakka I, et al. The impact of low-frequency and rare variants on lipid levels. Nature genetics. 2015;47(6):589-97.

3: Hoffmann TJ, et al. A large electronic-health-record-based genome-wide study of serum lipids. Nature genetics. 2018;50(3):401-13.

4: Bentley AR et al. Multi-ancestry genome-wide gene-smoking interaction study of 387,272 individuals identifies new loci associated with serum lipids. Nature genetics. 2019;51(4):636-48.

5: van Leeuwen EM et al. Meta-analysis of 49 549 individuals imputed with the 1000 Genomes Project reveals an exonic damaging variant in ANGPTL4 determining fasting TG levels. Journal of medical genetics. 2016;53(7):441-9.

6: Walter K, et al. The UK10K project identifies rare variants in health and disease. Nature. 2015;526(7571):82-90.

7: Klarin D, et al. Genetics of blood lipids among ~300,000 multi-ethnic participants of the Million Veteran Program. Nature genetics. 2018;50(11):1514-23.

8: Hu Y, et al. Minority-centric meta-analyses of blood lipid levels identify novel loci in the Population Architecture using Genomics and Epidemiology (PAGE) study. PLoS genetics. 2020;16(3):e1008684

**S4 Table: Logistic models for early TC increase ≥5% in the discovery sample**

|  | **Model with clinical variables** | | | **Model with clinical and  genetic variables** | | |
| --- | --- | --- | --- | --- | --- | --- |
|  | **Estimate** | **SE** | **p-value** | **Estimate** | **SE** | **p-value** |
| (Intercept) | 3.41 | 1.15 | **0.003** | 3.98 | 3.61 | 0.27 |
| Baseline BMI | -0.05 | 0.04 | 0.15 | -0.08 | 0.06 | 0.19 |
| Smoker | 0.19 | 0.34 | 0.58 | 0.22 | 0.52 | 0.67 |
| Baseline TC levels (mmol/l) | -0.53 | 0.17 | **0.002** | -0.86 | 0.29 | **0.003** |
| SNP (*GENE*)_effect allele |  |  |  |  |  |  |
| rs13114070T>C (*TMPRSS11E*)_T |  |  |  | -0.66 | 0.37 | 0.08 |
| rs12588415G>A (*YLPM1*)_G |  |  |  | 1.17 | 0.45 | **0.009** |
| rs2111485G>A (*unknown*)_A |  |  |  | 0.84 | 0.38 | **0.03** |
| rs6557781C>T (*DMTN*)_T |  |  |  | -1.25 | 0.64 | 0.05 |
| rs2287623A>G (*ABCB11*)_G |  |  |  | -0.89 | 0.38 | **0.02** |
| rs964184C>G (*AP006216.10; ZPR1*)_G |  |  |  | -1.25 | 0.54 | **0.02** |
| rs1077514T>C (*ASAP3*)_C |  |  |  | -0.66 | 0.55 | 0.23 |
| rs4738684G>A (*unknown*)_A |  |  |  | -1.36 | 0.42 | **0.001** |
| rs174554A>G (*FADS1; FADS2; MIR1908*)_A |  |  |  | 1.09 | 0.52 | **0.03** |
| rs1997243A>G (*AC073957.15; C7orf50; GPR146*)_A |  |  |  | -0.76 | 0.50 | 0.13 |
| rs1800961C>T (*HNF4A*)_C |  |  |  | 2.09 | 1.36 | 0.13 |
| rs2758886G>A (*unknown*)_G |  |  |  | 1.61 | 0.47 | **0.0006** |
| rs12027135T>A (*TMEM57*)_A |  |  |  | -0.89 | 0.40 | **0.02** |
| rs2618568A>C (*unknown*)_C |  |  |  | -0.31 | 0.36 | 0.39 |
| rs10128711C>T (*SPTY2D1; SPTY2D1-AS1*)_T |  |  |  | 1.10 | 0.44 | **0.01** |
| rs2954029A>T (*RP11-136O12.2*)_A |  |  |  | 0.66 | 0.37 | 0.07 |
| rs10904908A>G (*VIM-AS1*)_A |  |  |  | -1.88 | 0.49 | **0.0001** |

P-values in bold are significant. Abbreviation: BMI: body mass index; SE: standard error; SNP: single nucleotide polymorphism; TC: total cholesterol.

**S5 Table. Logistic models for early LDL-C increase ≥5% in the discovery sample**

|  | **Model with clinical variables** | | | **Model with clinical and  genetic variables** | | |
| --- | --- | --- | --- | --- | --- | --- |
|  | **Estimate** | **SE** | **p-value** | **Estimate** | **SE** | **p-value** |
| (Intercept) | 2.27 | 1.01 | **0.02** | -0.82 | 3.01 | 0.79 |
| Baseline BMI | -0.05 | 0.04 | 0.15 | -0.08 | 0.05 | 0.11 |
| Smoker | 0.20 | 0.35 | 0.56 | 0.47 | 0.46 | 0.31 |
| Baseline LDL-C levels (mmol/l) | -0.46 | 0.21 | **0.02** | -0.56 | 0.28 | 0.05 |
| SNP (*GENE*)_effect allele |  |  |  |  |  |  |
| rs2522061G>T (*AC116366.6 ; C5orf56 ; Y_RNA*)_G |  |  |  | 1.14 | 0.40 | **0.004** |
| rs7902274T>G (*RP11-564D11.3*)_G |  |  |  | 1.06 | 0.38 | **0.006** |
| rs704G>A (*CTB-96E2.2; CTB-96E2.3; CTB-96E2.7; SARM1; SEBOX; TMEM199; VTN*)_G |  |  |  | -0.88 | 0.35 | **0.01** |
| rs648324T>G (*PEX14*)_G |  |  |  | -0.45 | 0.35 | 0.20 |
| rs7538216C>T (*unknown*)_T |  |  |  | 0.55 | 0.37 | 0.14 |
| rs4773173A>G (*COL4A2*)_A |  |  |  | -0.92 | 0.37 | **0.01** |
| rs3812945T>C (*SCAMP5*)_T |  |  |  | -0.15 | 0.35 | 0.66 |
| rs28555129C>A (*OSGIN1; RP11-505K9.4*)_A |  |  |  | 0.42 | 0.36 | 0.24 |
| rs826682A>C (*LIMS1*)_A |  |  |  | -1.04 | 0.59 | 0.08 |
| rs14234A>G (*AC022201.5; FAM136A; SNRPG*)_A |  |  |  | 1.07 | 0.39 | **0.005** |
| rs17404153G>T (*DNAJC13*)_G |  |  |  | 0.64 | 0.48 | 0.18 |
| rs1030431G>A (*unknown*)_A |  |  |  | -0.88 | 0.37 | **0.02** |
| rs174583C>T (*FADS2*)_C |  |  |  | 0.42 | 0.40 | 0.29 |
| rs11136341A>G (*PLEC*)_A |  |  |  | -1.13 | 0.38 | **0.003** |
| rs1800562G>A (*HFE; HIST1H2BB*)_G |  |  |  | -1.34 | 0.79 | 0.09 |
| rs11648003A>G (*DHODH*)_A |  |  |  | 0.75 | 0.37 | **0.04** |
| rs2920503C>T (*PPARG*)_C |  |  |  | 1.07 | 0.43 | **0.01** |
| rs9987289G>A (*RP11-115J16.1*)_A |  |  |  | 1.85 | 0.87 | **0.03** |

P-values in bold are significant. Abbreviation: BMI: body mass index; LDL-C: low density lipoprotein cholesterol; SE: standard error; SNP: single nucleotide polymorphism.

**S6 Table. Logistic models for early HDL-C decrease ≥5% in the discovery sample**

|  | **Model with clinical  variables** | | | **Model with clinical and  genetic variables** | | |
| --- | --- | --- | --- | --- | --- | --- |
|  | **Estimate** | **SE** | **p-value** | **Estimate** | **SE** | **p-value** |
| (Intercept) | -3.90 | 0.90 | **1.55E-05** | -0.96 | 2.89 | 0.74 |
| Male gender | 0.92 | 0.39 | **0.02** | 1.11 | 0.54 | **0.04** |
| Baseline HDL-C levels (mmol/l) | 0.92 | 0.45 | **0.04** | 0.89 | 0.60 | 0.14 |
| Medication |  |  |  |  |  |  |
| Medium versus low risk | 1.39 | 0.58 | **0.01** | 1.11 | 0.72 | 0.13 |
| High versus low risk | 1.76 | 0.62 | **0.005** | 1.85 | 0.80 | **0.02** |
| SNP (*GENE*)_effect allele |  |  |  |  |  |  |
| rs12529923C>T (*GSTA10P; GSTA11P; GSTA3; GSTA5*)_C |  |  |  | 0.24 | 0.37 | 0.52 |
| rs17576323T>C (*AC007319.1*)_T |  |  |  | 0.41 | 0.38 | 0.28 |
| rs11727676T>C (*HHIP; uc_338*)_T |  |  |  | 1.72 | 0.86 | **0.04** |
| rs2683521G>A (*Metazoa_SRP; SP1*)_G |  |  |  | -0.49 | 0.36 | 0.17 |
| rs10494363G>A (*MTMR11; OTUD7B*)_G |  |  |  | 1.80 | 0.67 | **0.007** |
| rs10911505T>C (*unknown*)_T |  |  |  | 0.29 | 0.37 | 0.43 |
| rs1519480T>C (*BDNF; BDNF-AS*)_C |  |  |  | -0.71 | 0.42 | 0.09 |
| rs2373459T>C (*SPIC*)_C |  |  |  | -1.14 | 0.42 | **0.007** |
| rs1126930G>C (*PRKAG1; RP11-386G11.5*)_G |  |  |  | -2.71 | 1.06 | **0.01** |
| rs2280334C>T (*MEIS1; MEIS1-AS2; MEIS1-AS3*)_T |  |  |  | 1.08 | 0.43 | **0.01** |
| rs7730898A>G (*RANBP17*)_G |  |  |  | -0.72 | 0.47 | 0.13 |
| rs3173615C>G (*TMEM106B*)_C |  |  |  | 0.88 | 0.34 | **0.01** |
| rs3822072G>A (*FAM13A*)_G |  |  |  | -0.85 | 0.34 | **0.01** |
| rs970548A>C (*MARCH8*)_A |  |  |  | 0.38 | 0.37 | 0.31 |
| rs7134594T>C (*MMAB*)_C |  |  |  | 0.36 | 0.37 | 0.33 |
| rs11246602T>C (*OR4C46*)_T |  |  |  | 1.17 | 0.61 | 0.06 |
| rs7134375C>A (*unknown*)_C |  |  |  | -0.40 | 0.34 | 0.24 |
| rs11869286C>G (*STARD3*)_G |  |  |  | -0.51 | 0.36 | 0.16 |
| rs4765127G>T (*CCDC92; FAM101A; RP11-214K3.18; RP11-214K3.25; ZNF664*)_G |  |  |  | -0.48 | 0.35 | 0.17 |

P-values in bold are significant. Abbreviation: HDL-C: high density lipoprotein cholesterol; SE: standard error; SNP: single nucleotide polymorphism.

Psychotropic drugs were categorized into three categories depending on their risk of inducing metabolic disturbances, as described in the method section.

**S7 Table. Logistic models for early TG increase ≥5% in the discovery sample**

|  | **Model with clinical variables** | | | **Model with clinical and  genetic variables** | | |
| --- | --- | --- | --- | --- | --- | --- |
|  | **Estimate** | **SE** | **p-value** | **Estimate** | **SE** | **p-value** |
| (Intercept) | 1.17 | 0.48 | **0.01** | -4.23 | 2.79 | 0.13 |
| Male gender | 1.35 | 0.44 | **0.002** | 1.84 | 0.71 | **0.009** |
| Baseline TG levels (mmol/l) | -1.23 | 0.36 | **0.0006** | -1.18 | 0.55 | **0.03** |
| SNP (*GENE*)_effect allele |  |  |  |  |  |  |
| rs7519429A>C (*DNM3; PIGC*)_A |  |  |  | -0.82 | 0.42 | 0.05 |
| rs4683438G>T (*RP11-372E1.4*)_G |  |  |  | -0.84 | 0.45 | 0.06 |
| rs3851294G>A (*DSTYK*)_A |  |  |  | 1.05 | 0.86 | 0.22 |
| rs1133400A>G (*INPP5A*)_A |  |  |  | 1.77 | 0.60 | **0.003** |
| rs6430090G>A (*AC092484.1*)_A |  |  |  | 0.77 | 0.44 | 0.08 |
| rs10199914A>G (*AC114788.1*)_A |  |  |  | 0.93 | 0.47 | **0.04** |
| rs382534C>T (*SLC33A1*)_C |  |  |  | -1.46 | 0.62 | **0.02** |
| rs12206516A>G (*unknown*)_A |  |  |  | 0.68 | 0.52 | 0.18 |
| rs13266634C>T (*SLC30A8*)_C |  |  |  | 0.59 | 0.53 | 0.27 |
| rs4738141A>G (*RP11-1102P16.1*)_A |  |  |  | 0.85 | 0.59 | 0.15 |
| rs3843935C>T (*PRSS3; TRBV29OR9-2; UBE2R2-AS1*)_C |  |  |  | 0.99 | 0.50 | **0.04** |
| rs2131925T>G (*DOCK7*)_G |  |  |  | 1.26 | 0.60 | **0.03** |
| rs17585887C>T (*unknown*)_T |  |  |  | -0.03 | 0.49 | 0.95 |
| rs1495741A>G (*unknown*)_G |  |  |  | -1.71 | 0.65 | **0.009** |
| rs731839A>G (*PEPD*)_G |  |  |  | -0.52 | 0.53 | 0.33 |
| rs72959041G>A (*RSPO3*)_G |  |  |  | 2.81 | 1.19 | **0.02** |

P-values in bold are significant. Abbreviation: SE: standard error; SNP: single nucleotide polymorphism; TG: triglyceride.

**S8 Table: Demographic and clinical characteristics of the discovery sample**

| **Number of patients** | **177** | **TC < 5%** | **TC ≥ 5%** | **p-value** | **LDL-C < 5%** | **LDL-C ≥ 5 %** | **p-value** | **HDL-C > -5%** | **HDL-C ≤ -5%** | **p-value** | **TG < 5%** | **TG ≥ 5%** | **p-value** |
| --- | --- | --- | --- | --- | --- | --- | --- | --- | --- | --- | --- | --- | --- |
|  |  | **99** | **78** |  | **90** | **72** |  | **125** | **51** |  | **57** | **62** |  |
|  |  |  |  |  |  |  |  |  |  |  |  |  |  |
| Age, median (IQR), y | 41 (29-55) | 42 (31-55) | 40 (29-55) | 0.55 | 43 (29-57) | 40 (29-53) | 0.56 | 42 (29-55) | 40 (31-55) | 0.96 | 43 (32-55) | 37 (25-49) | 0.06 |
| Men, n(%) | 82 (46.3) | 43 (43.4) | 39 (50) | 0.47 | 40 (44.4) | 32 (44.4) | 1 | 52 (0.41) | 30 (58.8) | 0.06 | 20 (35.1) | 34 (54.8) | **0.048** |
| Smoking, n(%) | 77 (43.5) | 41 (41.4) | 36 (46.2) | 0.36 | 36 (40.0) | 33 (45.8) | 0.73 | 54 (0.43) | 23 (45.1) | 0.97 | 22 (38.6) | 29 (46.8) | 0.22 |
| Illness duration, median (IQR), y | 7 (2-12) | 7 (2-14.5) | 7.5 (2.8-10.3) | 0.92 | 7.5 (2-18.0) | 6 (2.5-10.0) | 0.78 | 8 (2-11) | 6 (3-17) | 0.69 | 8 (1.3-10.8) | 3.5 (1-10) | 0.66 |
| One-month visit, median (IQR), y | 31 (28-35) | 31 (28-34) | 31 (28-36) | 0.7 | 31 (28-34) | 30 (27-35) | 0.51 | 30 (28-34) | 31 (28-36) | 0.17 | 30 (28-34) | 31 (27-36) | 0.47 |
| Diagnosis, n(%) |  |  |  |  |  |  |  |  |  |  |  |  |  |
| Psychotic disorders | 44 (24.8) | 22 (22.2) | 22 (28.2) | 0.46 | 23 (25.5) | 18 (25.0) | 1 | 32 (0.26) | 12 (23.5) | 0.92 | 10 (17.5) | 19 (30.6) | 0.15 |
| Schizoaffective disorders | 9 (5.1) | 3 (3) | 6 (7.7) | 0.29 | 4 (4.4) | 5 (6.9) | 0.74 | 6 (4.8) | 3 (5.9) | 1 | 1 (1.8) | 2 (3.2) | 1 |
| Bipolar disorders | 37 (20.9) | 24 (24.2) | 13 (16.7) | 0.29 | 22 (24.4) | 11 (15.3) | 0.21 | 23 (18.4) | 13 (25.5) | 0.39 | 11 (19.3) | 13 (20.9) | 1 |
| Depressive disorders | 24 (13.6) | 11 (11.1) | 13 (16.7) | 0.39 | 9 (10) | 12 (16.7) | 0.31 | 19 (15.2) | 5 (9.8) | 0.48 | 11 (19.3) | 8 (12.9) | 0.48 |
| Organic disorders | 6 (3.4) | 4 (4) | 2 (2.5) | 0.9 | 3 (3.3) | 2 (2.8) | 1 | 4 (3.2) | 2 (3.9) | 1 | 2 (3.5) | 1 (1.6) | 0.94 |
| Other | 19 (10.7) | 10 (10.1) | 9 (11.5) | 0.95 | 9 (10) | 7 (9.7) | 1 | 14 (11.2) | 5 (9.8) | 0.99 | 6 (10.5) | 8 (12.9) | 0.91 |
| Not available | 38 (21.4) | 25 (25.2) | 13 (16.7) | 0.23 | 20 (22.2) | 17 (23.6) | 0.98 | 27 (21.6) | 11 (21.6) | 1 | 16 (28.1) | 11 (17.7) | 0.26 |
|  |  |  |  |  |  |  |  |  |  |  |  |  |  |
| Medication, n(%) |  |  |  |  |  |  |  |  |  |  |  |  |  |
| Amisulpride | 7 (3.9) | 3 (3) | 4 (5.1) | 0.76 | 3 (3.4) | 3 (4.2) | 1 | 7 (5.6) | 0 (0) | 0.19 | 3 (5.3) | 3 (4.8) | 1 |
| Amitriptyline | 2 (1.1) | 1 (1) | 1 (1.3) | 1 | 1 (1.1) | 1 (1.4) | 1 | 2 (1.6) | 0 (0) | 0.89 | 1 (1.7) | 1 (1.6) | 1 |
| Aripiprazole | 25 (14.2) | 16 (16.3) | 9 (11.5) | 0.49 | 18 (20.2) | 7 (9.7) | 0.11 | 23 (18.5) | 2 (3.9) | **0.02** | 11 (19.3) | 8 (12.9) | 0.48 |
| Brexpiprazole | 1 (0.5) | 1 (1) | 0 (0) | 1 | 1 (1.1) | 0 (0) | 1 | 1 (0.8) | 0 (0) | 1 | 0 (0) | 1 (1.6) | 1 |
| Carbamazepine | 3 (1.7) | 1 (1) | 2 (2.5) | 0.84 | 1 (1.1) | 2 (2.8) | 0.85 | 2 (1.6) | 1 (1.9) | 1 | 2 (3.5) | 1 (1.6) | 0.94 |
| Clozapine | 9 (5.1) | 6 (6.1) | 3 (3.8) | 0.74 | 6 (6.7) | 3 (4.2) | 0.72 | 7 (5.6) | 2 (3.9) | 0.92 | 4 (7.0) | 2 (3.2) | 0.6 |
| Haloperidol | 1 (0.5) | 0 (0) | 1 (1.3) | 0.91 | 0 (0) | 1 (1.4) | 0.91 | 1 (0.8) | 0 (0) | 1 | 0 (0) | 1 (1.6) | 1 |
| Lithium | 19 (10.8) | 15 (15.3) | 4 (5.1) | 0.055 | 13 (14.6) | 4 (5.6) | 0.11 | 14 (11.3) | 5 (9.8) | 0.98 | 7 (12.3) | 5 (8.1) | 0.65 |
| Lurasidone | 2 (1.1) | 2 (2) | 0 (0) | 0.58 | 2 (2.2) | 0 (0) | 0.57 | 2 (1.6) | 0 (0) | 0.89 | 2 (3.5) | 0 (0) | 0.44 |
| Mirtazapine | 18 (10.2) | 5 (5.1) | 13 (16.7) | **0.02** | 4 (4.5) | 9 (12.5) | 0.12 | 12 (9.7) | 6 (11.7) | 0.89 | 5 (8.8) | 9 (14.5) | 0.49 |
| Olanzapine | 24 (13.6) | 15 (15.3) | 9 (11.5) | 0.61 | 15 (16.8) | 7 (9.7) | 0.28 | 14 (11.3) | 10 (19.6) | 0.22 | 6 (10.5) | 9 (14.5) | 0.7 |
| Quetiapine | 44 (25.0) | 23 (23.5) | 21 (26.9) | 0.73 | 16 (17.9) | 25 (34.7) | **0.025** | 27 (21.8) | 16 (31.4) | 0.25 | 12 (21.0) | 17 (27.4) | 0.55 |
| Risperidone | 11 (6.2) | 5 (5.1) | 6 (7.7) | 0.69 | 4 (4.5) | 5 (6.9) | 0.74 | 7 (5.6) | 4 (7.8) | 0.84 | 2 (3.5) | 2 (3.2) | 1 |
| Valproate | 9 (5.1) | 5 (5.1) | 4 (5.1) | 1 | 5 (5.6) | 4 (5.6) | 1 | 5 (4.0) | 4 (7.8) | 0.51 | 2 (3.5) | 3 (4.8) | 1 |
| Zuclopenthixol | 1 (0.5) | 0 (0) | 1 (1.2) | 0.91 | 0 (0) | 1 (1.4) | 0.91 | 0 (0) | 1 (1.9) | 0.64 | 0 (0) | 0 (0) | 0.65 |
|  |  |  |  |  |  |  |  |  |  |  |  |  |  |
| Early weight gain (≥5%), n(%) | 21 (12.0) | 7 (7.1) | 14 (18.4) | **0.04** | 9 (10.1) | 10 (14.3) | 0.58 | 14 (11.3) | 7 (14.3) | 0.78 | 3 (5.3) | 12 (19.7) | **0.04** |

P-values were calculated using ranksum tests for continuous variables and Chi2 tests for categorical variables. Values in bold are significant. Abbreviation: IQR: interquartile range; HDL-C: high-density lipoprotein cholesterol; LDL-C: low-density lipoprotein cholesterol; TC: total cholesterol; TG: triglycerides.

**S9 Table: Demographic and clinical characteristics of the replication sample 1**

| **Number of patients** | **176** | **TC < 5%** | **TC ≥ 5%** | **p-value** | **LDL < 5%** | **LDL ≥ 5 %** | **p-value** | **HDL > -5%** | **HDL ≤ -5%** | **p-value** | **TG < 5%** | **TG ≥ 5%** | **p-value** |
| --- | --- | --- | --- | --- | --- | --- | --- | --- | --- | --- | --- | --- | --- |
|  |  | **93** | **83** |  | **84** | **81** |  | **98** | **75** |  | **60** | **56** |  |
|  |  |  |  |  |  |  |  |  |  |  |  |  |  |
| Age, median (IQR), y | 41 (29-54) | 43 (32-53) | 39 (28-55.5) | 0.44 | 42 (28.8-53.0) | 42 (29-56) | 0.5 | 39.5 (29-54.8) | 43 (32-54) | 0.76 | 42.5 (32-53) | 36 (24-49) | **0.02** |
| Men, n(%) | 88 (50.0) | 51 (54.8) | 37 (44.6) | 0.23 | 45 (53.6) | 32 (0.40) | 0.1 | 48 (49.0) | 37 (49.3) | 1 | 28 (46.7) | 32 (57.1) | 0.35 |
| Smoking, n(%) | 75 (42.6) | 43 (46.2) | 32 (38.6) | 0.59 | 41 (48.8) | 29 (0.36) | 0.16 | 46 (46.9) | 28 (37.3) | 0.43 | 29 (48.3) | 27 (48.2) | 0.86 |
| Illness duration, median (IQR), y | 4.5 (1-13) | 4 (2-10) | 6 (1-14) | 0.33 | 4 (1-10.3) | 6 (1.25-13.75) | 0.25 | 5.5 (1.3-13.0) | 4 (1.5-13) | 0.61 | 5.5 (1.3-15.2) | 4 (1-11) | 0.54 |
| One-month visit, median (IQR), d | 31 (28-34) | 30 (27-35) | 31 (28-34) | 0.6 | 31 (28-35) | 31 (28-33) | 0.44 | 31 (26-34) | 31 (28-35) | 0.19 | 30 (27-33) | 31 (27-34) | 0.79 |
| Diagnosis, n(%) |  |  |  |  |  |  |  |  |  |  |  |  |  |
| Psychotic disorders | 31 (17.6) | 14 (0.15) | 17 (20.5) | 0.46 | 11 (13.1) | 17 (20.9) | 0.25 | 13 (0.13) | 17 (22.7) | 0.16 | 10 (16.7) | 12 (21.4) | 0.68 |
| Schizoaffective disorders | 12 (21.1) | 4 (4.3) | 8 (9.6) | 0.27 | 5 (5.9) | 6 (7.4) | 0.95 | 7 (7.1) | 5 (6.7) | 1 | 3 (5.0) | 5 (8.9) | 0.64 |
| Bipolar disorders | 36 (20.4) | 21 (22.6) | 15 (18.1) | 0.58 | 18 (21.4) | 17 (20.9) | 1 | 17 (17.3) | 19 (25.3) | 0.27 | 11 (18.3) | 10 (17.9) | 1 |
| Depressive disorders | 31 (17.6) | 21 (22.6) | 10 (12.0) | 0.1 | 20 (23.8) | 11 (13.6) | 0.14 | 16 (16.3) | 15 (20.0) | 0.67 | 8 (13.3) | 10 (17.9) | 0.68 |
| Organic disorders | 4 (2.3) | 3 (3.2) | 1 (1.2) | 0.69 | 3 (3.6) | 1 (1.2) | 0.64 | 2 (2.0) | 2 (2.7) | 1 | 1 (1.7) | 0 (0) | 1 |
| Other | 23 (13.0) | 9 (9.7) | 14 (16.8) | 0.23 | 7 (8.3) | 13 (16.0) | 0.2 | 16 (16.3) | 6 (8.0) | 0.16 | 12 (20.0) | 4 (7.1) | 0.08 |
| Not available | 39 (22.1) | 21 (22.6) | 18 (21.7) | 1 | 20 (23.8) | 16 (19.7) | 0.66 | 27 (27.6) | 11 (14.7) | 0.06 | 15 (25.0) | 15 (26.8) | 0.99 |
|  |  |  |  |  |  |  |  |  |  |  |  |  |  |
| Medication, n(%) |  |  |  |  |  |  |  |  |  |  |  |  |  |
| Amisulpride | 12 (6.8) | 7 (7.5) | 5 (6.0) | 0.92 | 7 (8.3) | 5 (6.2) | 0.81 | 10 (10.2) | 2 (2.7) | 0.1 | 3 (5.0) | 5 (8.9) | 0.64 |
| Amitriptyline | 2 (1.1) | 1 (1.1) | 1 (1.2) | 1 | 1 (1.2) | 1 (1.2) | 1 | 2 (2.0) | 0 (0) | 0.59 | 2 (3.3) | 0 (0) | 0.51 |
| Aripiprazole | 26 (14.8) | 17 (18.3) | 9 (10.8) | 0.24 | 15 (17.8) | 8 (9.9) | 0.21 | 15 (15.3) | 11 (14.7) | 1 | 11 (18.3) | 5 (8.9) | 0.23 |
| Brexpiprazole | 0 (0) | 0 (0) | 0 (0) | 0.45 | 0 (0) | 0 (0) | 0.81 | 0 (0) | 0 (0) | 0.08 | 0 (0) | 0 (0) | 0.71 |
| Carbamazepine | 1 (0.5) | 0 (0) | 1 (1.2) | 0.95 | 0 (0) | 1 (1.2) | 0.98 | 0 (0) | 1 (1.3) | 0.89 | 1 (1.7) | 0 (0) | 1 |
| Clozapine | 10 (5.7) | 3 (3.2) | 7 (8.4) | 0.24 | 3 (3.6) | 7 (8.6) | 0.3 | 7 (7.1) | 3 (4.0) | 0.58 | 6 (10.0) | 2 (3.6) | 0.32 |
| Haloperidol | 4 (2.3) | 2 (2.2) | 2 (2.4) | 1 | 1 (1.2) | 3 (3.7) | 0.59 | 1 (1.0) | 3 (4.0) | 0.43 | 3 (5.0) | 0 (0) | 0.27 |
| Lithium | 17 (9.7) | 8 (8.6) | 9 (10.8) | 0.81 | 8 (9.5) | 8 (9.9) | 1 | 12 (12.2) | 5 (6.7) | 0.33 | 3 (5.0) | 6 (10.7) | 0.42 |
| Lurasidone | 3 (1.7) | 2 (2.2) | 1 (1.2) | 1 | 1 (1.2) | 2 (2.5) | 0.97 | 1 (1.0) | 2 (2.7) | 0.81 | 0 (0) | 0 (0) | 0.71 |
| Mirtazapine | 21 (11.9) | 13 (14.0) | 8 (9.6) | 0.51 | 14 (16.7) | 6 (7.4) | 0.11 | 13 (13.2) | 7 (9.3) | 0.57 | 10 (16.7) | 5 (8.9) | 0.33 |
| Olanzapine | 22 (12.5) | 11 (11.8) | 11 (13.3) | 0.95 | 8 (9.5) | 12 (14.8) | 0.42 | 9 (9.2) | 12 (16.0) | 0.26 | 5 (8.3) | 8 (14.2) | 0.47 |
| Quetiapine | 33 (18.7) | 16 (17.2) | 17 (20.5) | 0.72 | 15 (17.8) | 16 (19.7) | 0.91 | 12 (12.2) | 20 (26.7) | **0.03** | 9 (15.0) | 16 (28.6) | 0.12 |
| Risperidone | 16 (9.1) | 9 (9.7) | 7 (8.4) | 0.98 | 6 (7.1) | 9 (11.1) | 0.54 | 12 (12.2) | 4 (5.3) | 0.19 | 4 (6.7) | 6 (10.7) | 0.66 |
| Valproate | 9 (5.1) | 4 (4.3) | 5 (6.0) | 0.86 | 5 (5.9) | 3 (3.7) | 0.76 | 4 (4.1) | 5 (6.7) | 0.68 | 3 (5.0) | 3 (5.4) | 1 |
| Zuclopenthixol | 0 (0) | 0 (0) | 0 (0) | 0.45 | 0 (0) | 0 (0) | 0.81 | 0 (0) | 0 (0) | 0.08 | 0 (0) | 0 (0) | 0.71 |
|  |  |  |  |  |  |  |  |  |  |  |  |  |  |
| Early weight gain (≥5%), n(%) | 35 (20.4) | 13 (14.0) | 22 (27.2) | 0.06 | 13 (15.9) | 18 (23.1) | 0.34 | 25 (25.8) | 9 (12.7) | 0.06 | 14 (23.7) | 9 (16.7) | 0.49 |

P-values were calculated using ranksum tests for continuous variables and Chi2 tests for categorical variables. Values in bold are significant; Abbreviation: IQR: interquartile range; HDL-C: high-density lipoprotein cholesterol; LDL-C: low-density lipoprotein cholesterol; TC: total cholesterol; TG: triglycerides.

**S10 Table: Demographic and clinical characteristics of the replication sample 2**

| **Number of patients** | **86** | **TC < 5%** | **TC ≥ 5%** | **p-value** | **LDL < 5%** | **LDL ≥ 5 %** | **p-value** | **HDL > -5%** | **HDL ≤ -5%** | **p-value** | **TG < 5%** | **TG ≥ 5%** | **p-value** |
| --- | --- | --- | --- | --- | --- | --- | --- | --- | --- | --- | --- | --- | --- |
|  |  | **54** | **27** |  | **41** | **28** |  | **49** | **30** |  | **27** | **36** |  |
|  |  |  |  |  |  |  |  |  |  |  |  |  |  |
| Age, median (IQR), y | 42 (33-52) | 42 (33-53) | 36 (33-53) | 0.39 | 40 (31-52) | 39 (34-55) | 0.72 | 43 (33-55) | 36 (28-52) | 0.13 | 46 (40-55) | 37 (31-54) | 0.21 |
| Men, n(%) | 38 (44.7) | 21 (39.6) | 14 (51.8) | 0.42 | 15 (37.5) | 13 (46.4) | 0.63 | 21 (43.8) | 14 (46.7) | 0.98 | 11 (40.7) | 18 (51.4) | 0.56 |
| Smoking, n(%) | 37 (43.0) | 20 (37) | 16 (59.3) | 0.09 | 19 (46.3) | 13 (46.4) | 1 | 20 (40.8) | 14 (46.7) | 0.78 | 10 (37) | 19 (52.8) | 0.32 |
| Illness duration, median (IQR), y | 6.5 (2-13) | 3 (2.5-10.5) | 5.5 (3.3-7.8) | 0.8 | 10 (6-14) | 10 (10-10) | 1 | 3 (2.5-6.5) | 18 (18-18) | 0.5 | 6 (4-8) | 3 (2-10.5) | 1 |
| One-month visit, median (IQR), d | 34 (30-37) | 34 (29-36) | 34 (32-37) | 0.82 | 33 (29-35) | 35 (30-36) | 0.45 | 34 (29-35) | 34 (32-37) | 0.31 | 35 (32-36) | 34 (30-37) | 0.94 |
| Diagnosis, n(%) |  |  |  |  |  |  |  |  |  |  |  |  |  |
| Psychotic disorders | 1 (1.1) | 0 (0) | 0 (0) | NA | 0 (0) | 0 (0) | NA | 0 (0) | 0 (0) | NA | 0 (0) | 0 (0) | NA |
| Schizoaffective disorders | 1 (1.1) | 1 (1.8) | 0 (0) | NA | 1 (2.4) | 0 (0) | NA | 1 (2) | 0 (0) | NA | 1 (3.7) | 0 (0) | NA |
| Bipolar disorders | 2 (2.3) | 2 (3.7) | 0 (0) | NA | 2 (4.9) | 0 (0) | NA | 1 (2) | 1 (3.3) | 1 | 0 (0) | 0 (0) | NA |
| Depressive disorders | 9 (10.4) | 5 (9.3) | 3 (11.1) | 0.48 | 4 (9.7) | 4 (14.3) | 1 | 8 (16.3) | 0 (0) | NA | 5 (18.5) | 3 (8.3) | 0.48 |
| Organic disorders | 0 (0) | 0 (0) | 0 (0) | NA | 0 (0) | 0 (0) | NA | 0 (0) | 0 (0) | NA | 0 (0) | 0 (0) | NA |
| Other | 4 (4.6) | 3 (5.6) | 1 (3.7) | 0.32 | 2 (4.9) | 2 (7.1) | 1 | 3 (6.1) | 1 (3.3) | 0.32 | 1 (3.7) | 2 (5.6) | 0.56 |
| Not available | 69 (80.2) | 43 (79.6) | 23 (85.2) | **0.01** | 32 (78) | 22 (78.6) | 0.17 | 36 (73.4) | 28 (93.3) | 0.31 | 20 (74) | 31 (86.1) | 0.12 |
|  |  |  |  |  |  |  |  |  |  |  |  |  |  |
| Medication, n(%) |  |  |  |  |  |  |  |  |  |  |  |  |  |
| Amisulpride | 3 (3.5) | 2 (3.7) | 0 (0) | NA | 2 (4.9) | 0 (0) | NA | 1 (2) | 1 (3.3) | 1 | 1 (3.7) | 1 (2.8) | 1 |
| Amitriptyline | 3 (3.5) | 1 (1.8) | 2 (7.4) | 0.56 | 0 (0) | 2 (7.1) | NA | 1 (2) | 2 (6.6) | 0.56 | 0 (0) | 2 (5.6) | NA |
| Aripiprazole | 16 (18.6) | 12 (22.2) | 4 (14.8) | **0.04** | 7 (17.1) | 8 (28.6) | 0.79 | 10 (20.4) | 6 (20.0) | 0.32 | 7 (25.9) | 7 (19.4) | 1 |
| Brexpiprazole | 1 (1.1) | 1 (1.8) | 0 (0) | NA | 0 (0) | 0 (0) | NA | 1 (2) | 0 (0) | NA | 0 (0) | 1 (2.8) | NA |
| Carbamazepine | 2 (2.3) | 0 (0) | 2 (7.4) | NA | 0 (0) | 2 (7.1) | NA | 2 (4.1) | 0 (0) | NA | 0 (0) | 1 (2.8) | NA |
| Levomepromazine | 1 (1.1) | 0 (0) | 0 (0) | NA | 0 (0) | 0 (0) | NA | 0 (0) | 0 (0) | NA | 0 (0) | 0 (0) | NA |
| Lithium | 6 (6.9) | 4 (7.4) | 2 (7.4) | 0.41 | 5 (12.2) | 0 (0) | NA | 5 (10.2) | 1 (3.3) | 0.1 | 2 (7.4) | 2 (5.6) | 1 |
| Lurasidone | 4 (4.6) | 2 (3.7) | 2 (7.4) | 1 | 2 (4.9) | 1 (3.6) | 0.56 | 1 (2) | 3 (10.0) | 0.32 | 1 (3.7) | 2 (5.6) | 0.56 |
| Mirtazapine | 19 (22.1) | 8 (14.8) | 9 (33.3) | 0.8 | 7 (17.1) | 9 (32.1) | 0.62 | 13 (26.5) | 4 (13.3) | **0.03** | 7 (25.9) | 7 (19.4) | 1 |
| Olanzapine | 3 (3.5) | 1 (1.9) | 1 (3.7) | 1 | 0 (0) | 1 (3.6) | NA | 0 (0) | 1 (3.3) | NA | 1 (3.7) | 1 (2.8) | 1 |
| Quetiapine | 25 (29.0) | 20 (37) | 5 (18.5) | **0.002** | 16 (39) | 5 (17.8) | **0.01** | 12 (24.5) | 12 (40.0) | 1 | 7 (25.9) | 11 (30.6) | 0.35 |
| Risperidone | 2 (2.3) | 2 (3.7) | 0 (0) | NA | 1 (2.4) | 0 (0) | NA | 2 (4.1) | 0 (0) | NA | 1 (3.7) | 1 (2.8) | 1 |
| Valproate | 1 (1.1) | 1 (1.8) | 0 (0) | NA | 1 (2.4) | 0 (0) | NA | 1 (2) | 0 (0) | NA | 0 (0) | 0 (0) | NA |
|  |  |  |  |  |  |  |  |  |  |  |  |  |  |
| Early weight gain (≥5%), n(%) | 13 (15.1) | 8 (14.8) | 5 (18.5) | 0.72 | 7 (17.1) | 3 (10.7) | 0.52 | 2 (4.1) | 10 (33.3) | **0.001** | 2 (7.4) | 9 (25) | 0.11 |

P-values were calculated using ranksum tests for continuous variables and Chi2 tests for categorical variables. Values in bold are significant; Abbreviation: IQR: interquartile range; HDL-C: high-density lipoprotein cholesterol; LDL-C: low-density lipoprotein cholesterol; TC: total cholesterol; TG: triglycerides.

**S11 Table: Comparison of demographic and clinical characteristics between the discovery and replication samples**

| **Number of patients** | **Discovery sample** | **Replication sample 1** | **Replication sample 2** | **p-value** |
| --- | --- | --- | --- | --- |
|  | **177** | **176** | **86** |  |
|  |  |  |  |  |
| Age, median (IQR), y | 41 (29-55) | 41 (29-54) | 42 (33-52) | 0.73 |
| Men, n(%) | 82 (46.3) | 88 (50.0) | 38 (44.7) | 0.67 |
| Smoking, n(%) | 77 (43.5) | 75 (42.6) | 37 (43.0) | 0.69 |
| Illness duration, median (IQR), y | 7 (2-12) | 4.5 (1-13) | 6.5 (2-13) | 0.44 |
| One-month visit, median (IQR), d | 31 (28-35) | 31 (28-34) | 34 (30-37) | **0.002** |
| Diagnosis, n(%) |  |  |  |  |
| Psychotic disorders | 44 (24.8) | 31 (17.6) | 1 (1.1) | **4.60E-09** |
| Schizoaffective disorders | 9 (5.1) | 12 (21.1) | 1 (1.1) | **0.01** |
| Bipolar disorders | 37 (20.9) | 36 (20.4) | 2 (2.3) | **1.30E-07** |
| Depressive disorders | 24 (13.6) | 31 (17.6) | 9 (10.4) | **0.003** |
| Organic disorders | 6 (3.4) | 4 (2.3) | 0 (0) | 0.53 |
| Other | 19 (10.7) | 23 (13.0) | 4 (4.6) | **0.001** |
| Not available | 38 (21.4) | 39 (22.1) | 69 (80.2) | **0.002** |
| Medication, n(%) |  |  |  |  |
| Amisulpride | 7 (3.9) | 12 (6.8) | 3 (3.5) | 0.06 |
| Amitriptyline | 2 (1.1) | 2 (1.1) | 3 (3.5) | 0.87 |
| Aripiprazole | 25 (14.2) | 26 (14.8) | 16 (18.6) | 0.26 |
| Brexpiprazole | 1 (0.5) | 0 (0) | 1 (1.1) | 1 |
| Carbamazepine | 3 (1.7) | 1 (0.5) | 2 (2.3) | 0.61 |
| Clozapine | 9 (5.1) | 10 (5.7) | 0 (0) | 0.82 |
| Haloperidol | 1 (0.5) | 4 (2.3) | 0 (0) | 0.18 |
| Levomepromazine | 0 (0) | 0 (0) | 1 (1.1) | NA |
| Lithium | 19 (10.8) | 17 (9.7) | 6 (6.9) | **0.03** |
| Lurasidone | 2 (1.1) | 3 (1.7) | 4 (4.6) | 0.72 |
| Mirtazapine | 18 (10.2) | 21 (11.9) | 19 (22.1) | 0.89 |
| Olanzapine | 24 (13.6) | 22 (12.5) | 3 (3.5) | **0.0002** |
| Quetiapine | 44 (25.0) | 33 (18.7) | 25 (29.0) | 0.07 |
| Risperidone | 11 (6.2) | 16 (9.1) | 2 (2.3) | **0.005** |
| Valproate | 9 (5.1) | 9 (5.1) | 1 (1.1) | **0.03** |
| Zuclopenthixol | 1 (0.5) | 0 (0) | 0 (0) | NA |
|  |  |  |  |  |
| Early weight gain (≥5%), n(%) | 21 (12.0) | 35 (20.4) | 13 (15.1) | 0.1 |
| Early TC increase (≥5%), n(%) | 78 (44.1) | 83 (47.1) | 27 (33.3) | 0.11 |
| Early LDL-C increase (≥5%), n(%) | 72 (44.4) | 81 (49.1) | 28 (40.5) | 0.45 |
| Early HDL-C decrease (≥5%), n(%) | 51 (28.9) | 75 (43.3) | 30 (38.0) | **0.02** |
| Early TG increase (≥5%), n(%) | 62 (52.1) | 56 (48.3) | 36 (57.1) | 0.86 |
|  |  |  |  |  |

P-values were calculated using ANOVA for continuous variables and Chi2 tests for categorical variables. Values in bold are significant. Abbreviation: IQR: interquartile range; HDL-C: high-density lipoprotein cholesterol; LDL-C: low-density lipoprotein cholesterol; TC: total cholesterol; TG: triglycerides.

**S12 Table: Logistic models for early TC increase ≥5% in replication sample 1**

|  | **Model with clinical variables** | | | **Model with clinical and  genetic variables** | | |
| --- | --- | --- | --- | --- | --- | --- |
|  | **Estimate** | **SE** | **p-value** | **Estimate** | **SE** | **p-value** |
| (Intercept) | 5.25 | 1.23 | **0.00002** | 4.80 | 3.41 | 0.16 |
| Baseline BMI | -0.02 | 0.03 | 0.62 | -0.02 | 0.04 | 0.58 |
| Smoker | -0.68 | 0.38 | 0.08 | -0.63 | 0.41 | 0.12 |
| Baseline TC levels (mmol/l) | -0.98 | 0.20 | **0.000001** | -1.06 | 0.23 | **0.000003** |
| SNP (*GENE*)_effect allele |  |  |  |  |  |  |
| rs13114070T>C (*TMPRSS11E*)_T |  |  |  | -0.09 | 0.35 | 0.80 |
| rs12588415G>A (*YLPM1*)_G |  |  |  | 0.31 | 0.33 | 0.35 |
| rs2111485G>A (*unknown*)_A |  |  |  | -0.27 | 0.29 | 0.35 |
| rs6557781C>T (*DMTN*)_T |  |  |  | 0.08 | 0.38 | 0.84 |
| rs2287623A>G (*ABCB11*)_G |  |  |  | 0.19 | 0.30 | 0.53 |
| rs964184C>G (*AP006216.10; ZPR1*)_G |  |  |  | 0.21 | 0.44 | 0.64 |
| rs1077514T>C (*ASAP3*)_C |  |  |  | -0.16 | 0.42 | 0.71 |
| rs4738684G>A (*unknown*)_A |  |  |  | 0.10 | 0.30 | 0.74 |
| rs174554A>G (*FADS1; FADS2; MIR1908*)_A |  |  |  | -0.17 | 0.32 | 0.59 |
| rs1997243A>G (*AC073957.15; C7orf50; GPR146*)_A |  |  |  | -0.03 | 0.38 | 0.94 |
| rs1800961C>T (*HNF4A*)_C |  |  |  | 0.65 | 1.30 | 0.62 |
| rs2758886G>A (*unknown*)_G |  |  |  | -0.23 | 0.33 | 0.49 |
| rs12027135T>A (*TMEM57*)_A |  |  |  | 0.24 | 0.30 | 0.42 |
| rs2618568A>C (*unknown*)_C |  |  |  | 0.21 | 0.29 | 0.47 |
| rs10128711C>T (*SPTY2D1; SPTY2D1-AS1*)_T |  |  |  | -0.14 | 0.37 | 0.70 |
| rs2954029A>T (*RP11-136O12.2*)_A |  |  |  | -0.05 | 0.33 | 0.87 |
| rs10904908A>G (*VIM-AS1*)_A |  |  |  | -0.32 | 0.32 | 0.31 |

P-values in bold are significant. Abbreviation: BMI: body mass index; SE: standard error; SNP: single nucleotide polymorphism; TC: total cholesterol.

**S13 Table: Logistic models for early LDL-C increase ≥5% in replication sample 1**

|  | **Model with clinical variables** | | | **Model with clinical and  genetic variables** | | |
| --- | --- | --- | --- | --- | --- | --- |
|  | **Estimate** | **SE** | **p-value** | **Estimate** | **SE** | **p-value** |
| (Intercept) | 3.39 | 1.03 | **0.001** | -0.83 | 2.84 | 0.77 |
| Baseline BMI | -0.03 | 0.03 | 0.46 | -0.06 | 0.04 | 0.14 |
| Smoker | -0.75 | 0.38 | **0.05** | -1.09 | 0.48 | **0.02** |
| Baseline LDL-C levels (mmol/l) | -0.94 | 0.23 | **0.00004** | -1.04 | 0.28 | **0.0002** |
| SNP (*GENE*)_effect allele |  |  |  |  |  |  |
| rs2522061G>T (*AC116366.6; C5orf56; Y_RNA*)_G |  |  |  | 0.86 | 0.42 | **0.04** |
| rs7902274T>G (*RP11-564D11.3*)_G |  |  |  | -0.26 | 0.31 | 0.41 |
| rs704G>A (*CTB-96E2.2; CTB-96E2.3; CTB-96E2.7; SARM1; SEBOX; TMEM199; VTN*)_G |  |  |  | -0.01 | 0.33 | 0.98 |
| rs648324T>G (*PEX14*)_G |  |  |  | 0.44 | 0.40 | 0.27 |
| rs7538216C>T (*unknown*)_T |  |  |  | -0.12 | 0.33 | 0.70 |
| rs4773173A>G (*COL4A2*)_A |  |  |  | 0.14 | 0.34 | 0.68 |
| rs3812945T>C (*SCAMP5*)_T |  |  |  | 0.37 | 0.33 | 0.26 |
| rs28555129C>A (*OSGIN1; RP11-505K9.4*)_A |  |  |  | 0.01 | 0.36 | 0.98 |
| rs826682A>C (*LIMS1*)_A |  |  |  | -0.63 | 0.48 | 0.19 |
| rs14234A>G (*AC022201.5; FAM136A; SNRPG*)_A |  |  |  | 0.40 | 0.35 | 0.26 |
| rs17404153G>T (*DNAJC13*)_G |  |  |  | 0.15 | 0.44 | 0.74 |
| rs1030431G>A (*unknown*)_A |  |  |  | -0.99 | 0.37 | **0.01** |
| rs174583C>T (*FADS2*)_C |  |  |  | -0.63 | 0.36 | 0.08 |
| rs11136341A>G (*PLEC*)_A |  |  |  | -0.25 | 0.35 | 0.47 |
| rs1800562G>A (*HFE; HIST1H2BB*)_G |  |  |  | 1.48 | 1.02 | 0.15 |
| rs11648003A>G (*DHODH*)_A |  |  |  | 0.66 | 0.40 | 0.10 |
| rs2920503C>T (*PPARG*)_C |  |  |  | -0.10 | 0.34 | 0.78 |
| rs9987289G>A (*RP11-115J16.1*)_A |  |  |  | 0.94 | 0.75 | 0.21 |

P-values in bold are significant. Abbreviation: BMI: body mass index; LDL-C: Low-density lipoprotein cholesterol; SE: standard error; SNP: single nucleotide polymorphism.

**S14 Table: Logistic models for early HDL-C decrease ≥5% in replication sample 1**

|  | **Model with clinical  variables** | | | **Model with clinical and  genetic variables** | | |  |
| --- | --- | --- | --- | --- | --- | --- | --- |
|  | **Estimate** | **SE** | **p-value** | **Estimate** | **SE** | **p-value** | |
| (Intercept) | -4.24 | 0.90 | **0.000003** | -0.60 | 3.10 | 0.85 | |
| Male gender | 0.95 | 0.39 | **0.02** | 1.36 | 0.47 | **0.004** | |
| Baseline HDL-C levels (mmol/l) | 2.43 | 0.54 | **0.000007** | 2.87 | 0.66 | **0.00001** | |
| Medication |  |  |  |  |  |  | |
| Medium versus low risk | -0.07 | 0.41 | 0.86 | -0.18 | 0.47 | 0.70 | |
| High versus low risk | 0.33 | 0.47 | 0.48 | 0.29 | 0.57 | 0.62 | |
| SNP (*GENE*)_effect allele |  |  |  |  |  |  | |
| rs12529923C>T (*GSTA10P; GSTA11P; GSTA3; GSTA5*)_C |  |  |  | 0.20 | 0.30 | 0.51 | |
| rs17576323T>C (*AC007319.1*)_T |  |  |  | -0.17 | 0.38 | 0.65 | |
| rs11727676T>C (*HHIP; uc_338*)_T |  |  |  | 0.38 | 0.55 | 0.50 | |
| rs2683521G>A (*Metazoa_SRP; SP1*)_G |  |  |  | -0.48 | 0.26 | 0.07 | |
| rs10494363G>A (*MTMR11; OTUD7B*)_G |  |  |  | -1.57 | 0.69 | **0.02** | |
| rs10911505T>C (*unknown*)_T |  |  |  | 0.64 | 0.33 | **0.05** | |
| rs1519480T>C (*BDNF; BDNF-AS*)_C |  |  |  | -0.19 | 0.31 | 0.55 | |
| rs2373459T>C (*SPIC*)_C |  |  |  | -0.25 | 0.29 | 0.38 | |
| rs1126930G>C (*PRKAG1; RP11-386G11.5*)_G |  |  |  | -2.60 | 1.38 | 0.06 | |
| rs2280334C>T (*MEIS1; MEIS1-AS2; MEIS1-AS3*)_T |  |  |  | 0.03 | 0.32 | 0.92 | |
| rs7730898A>G (*RANBP17*)_G |  |  |  | -0.16 | 0.30 | 0.58 | |
| rs3173615C>G (*TMEM106B*)_C |  |  |  | -0.40 | 0.30 | 0.18 | |
| rs3822072G>A (*FAM13A*)_G |  |  |  | 0.16 | 0.29 | 0.58 | |
| rs970548A>C (*MARCH8*)_A |  |  |  | -0.12 | 0.34 | 0.72 | |
| rs7134594T>C (*MMAB*)_C |  |  |  | -0.35 | 0.30 | 0.24 | |
| rs11246602T>C (*OR4C46*)_T |  |  |  | 1.07 | 0.49 | **0.03** | |
| rs7134375C>A (*unknown*)_C |  |  |  | -0.03 | 0.30 | 0.91 | |
| rs11869286C>G (*STARD3*)_G |  |  |  | 0.55 | 0.32 | 0.08 | |
| rs4765127G>T (*CCDC92; FAM101A; RP11-214K3.18; RP11-214K3.25; ZNF664*)_G |  |  |  | 0.10 | 0.28 | 0.72 | |

P-values in bold are significant. Abbreviation: HDL-C: High-density lipoprotein cholesterol; SE: standard error; SNP: single nucleotide polymorphism. Psychotropic drugs were categorized into three categories depending on their risk of inducing metabolic disturbances, as described in the method section.

**S15 Table: Logistic models for early TG increase ≥5% in replication sample 1**

|  | **Model with clinical variables** | | | **Model with clinical and  genetic variables** | | | |
| --- | --- | --- | --- | --- | --- | --- | --- |
|  | **Estimate** | **SE** | **p-value** | | **Estimate** | **SE** | **p-value** |
| (Intercept) | 1.05 | 0.50 | **0.03** | | -0.55 | 1.59 | 0.73 |
| Male gender | 0.65 | 0.40 | 0.11 | | 0.70 | 0.44 | 0.11 |
| Baseline TG levels (mmol/l) | -1.14 | 0.36 | **0.002** | | -1.32 | 0.42 | **0.002** |
| SNP (*GENE*)_effect allele |  |  |  | |  |  |  |
| rs7519429A>C (*DNM3; PIGC*)_A |  |  |  | | -0.01 | 0.31 | 0.99 |
| rs4683438G>T (*RP11-372E1.4*)_G |  |  |  | | -0.15 | 0.33 | 0.65 |
| rs3851294G>A (*DSTYK*)_A |  |  |  | | 0.12 | 0.60 | 0.84 |
| rs1133400A>G (*INPP5A*)_A |  |  |  | | -0.18 | 0.35 | 0.60 |
| rs6430090G>A (*AC092484.1*)_A |  |  |  | | -0.09 | 0.34 | 0.79 |
| rs10199914A>G (*AC114788.1*)_A |  |  |  | | 0.15 | 0.35 | 0.68 |
| rs382534C>T (*SLC33A1*)_C |  |  |  | | -0.13 | 0.33 | 0.69 |
| rs12206516A>G (*unknown*)_A |  |  |  | | -0.04 | 0.39 | 0.91 |
| rs13266634C>T (*SLC30A8*)_C |  |  |  | | 0.25 | 0.34 | 0.47 |
| rs4738141A>G (*RP11-1102P16.1*)_A |  |  |  | | 0.03 | 0.36 | 0.94 |
| rs3843935C>T (*PRSS3; TRBV29OR9-2; UBE2R2-AS1*)_C |  |  |  | | 0.25 | 0.31 | 0.42 |
| rs2131925T>G (*DOCK7*)_G |  |  |  | | 0.48 | 0.34 | 0.15 |
| rs17585887C>T (*unknown*)_T |  |  |  | | 0.41 | 0.33 | 0.21 |
| rs1495741A>G (*unknown*)_G |  |  |  | | -0.06 | 0.39 | 0.87 |
| rs731839A>G (*PEPD*)_G |  |  |  | | 0.69 | 0.36 | 0.06 |
| rs72959041G>A (*RSPO3*)_G |  |  |  | | -0.28 | 0.66 | 0.68 |

P-values in bold are significant. Abbreviation; SE: standard error; SNP: single nucleotide polymorphism; TG: triglycerides.

**S16 Table: Logistic models for early TC increase ≥5% in replication sample 2**

|  | **Model with clinical variables** | | | **Model with clinical and  genetic variables** | | |
| --- | --- | --- | --- | --- | --- | --- |
|  | **Estimate** | **SE** | **p-value** | **Estimate** | **SE** | **p-value** |
| (Intercept) | 0.45 | 1.47 | 0.76 | -35.83 | 3790.00 | 0.99 |
| Baseline BMI | 0.00 | 0.04 | 0.93 | 0.01 | 0.05 | 0.88 |
| Smoker | 0.92 | 0.51 | 0.07 | 1.25 | 0.75 | 0.10 |
| Baseline TC levels (mmol/l) | -0.29 | 0.24 | 0.22 | -0.50 | 0.33 | 0.13 |
| SNP (*GENE*)_effect allele |  |  |  |  |  |  |
| rs13114070T>C (*TMPRSS11E*)_T |  |  |  | -0.03 | 0.48 | 0.95 |
| rs12588415G>A (*YLPM1*)_G |  |  |  | -0.26 | 0.61 | 0.67 |
| rs2111485G>A (*unknown*)_A |  |  |  | -0.34 | 0.52 | 0.51 |
| rs6557781C>T (*DMTN*)_T |  |  |  | 0.90 | 0.66 | 0.17 |
| rs2287623A>G (*ABCB11*)_G |  |  |  | -0.33 | 0.52 | 0.53 |
| rs964184C>G (*AP006216.10; ZPR1*)_G |  |  |  | -0.53 | 0.72 | 0.46 |
| rs1077514T>C (*ASAP3*)_C |  |  |  | -0.14 | 0.74 | 0.85 |
| rs4738684G>A (*unknown*)_A |  |  |  | -0.20 | 0.51 | 0.69 |
| rs174554A>G (*FADS1; FADS2; MIR1908*)_A |  |  |  | 1.29 | 0.63 | **0.04** |
| rs1997243A>G (*AC073957.15; C7orf50; GPR146*)_A |  |  |  | 0.02 | 0.61 | 0.97 |
| rs1800961C>T (*HNF4A*)_C |  |  |  | 16.67 | 1895.00 | 0.99 |
| rs2758886G>A (*unknown*)_G |  |  |  | 1.06 | 0.61 | 0.08 |
| rs12027135T>A (*TMEM57*)_A |  |  |  | 0.05 | 0.47 | 0.91 |
| rs2618568A>C (*unknown*)_C |  |  |  | 0.32 | 0.51 | 0.53 |
| rs10128711C>T (*SPTY2D1; SPTY2D1-AS1*)_T |  |  |  | -0.43 | 0.59 | 0.46 |
| rs2954029A>T (*RP11-136O12.2*)_A |  |  |  | 0.74 | 0.60 | 0.22 |
| rs10904908A>G (*VIM-AS1*)_A |  |  |  | 1.00 | 0.58 | 0.08 |

P-values in bold are significant. Abbreviation: BMI: body mass index; SE: standard error; SNP: single nucleotide polymorphism; TC: total cholesterol.

**S17 Table: Logistic models for early LDL-C increase ≥5% in replication sample 2**

|  | **Model with clinical variables** | | | **Model with clinical and  genetic variables** | | |
| --- | --- | --- | --- | --- | --- | --- |
|  | **Estimate** | **SE** | **p-value** | **Estimate** | **SE** | **p-value** |
| (Intercept) | 2.18 | 1.32 | 0.10 | -33.15 | 3528.00 | 0.99 |
| Baseline BMI | -0.03 | 0.04 | 0.39 | -0.03 | 0.06 | 0.61 |
| Smoker | 0.25 | 0.53 | 0.64 | 0.13 | 0.90 | 0.89 |
| Baseline LDL-C levels (mmol/l) | -0.59 | 0.31 | **0.05** | -0.97 | 0.63 | 0.12 |
| SNP (*GENE*)_effect allele |  |  |  |  |  |  |
| rs2522061G>T (*AC116366.6; C5orf56; Y_RNA*)_G |  |  |  | 0.00 | 0.78 | 1.00 |
| rs7902274T>G (*RP11-564D11.3*)_G |  |  |  | -1.48 | 0.72 | **0.04** |
| rs704G>A (*CTB-96E2.2; CTB-96E2.3; CTB-96E2.7; SARM1; SEBOX; TMEM199; VTN*)_G |  |  |  | 0.29 | 0.66 | 0.66 |
| rs648324T>G (*PEX14*)_G |  |  |  | 0.36 | 0.77 | 0.64 |
| rs7538216C>T (*unknown*)_T |  |  |  | -1.60 | 0.92 | 0.08 |
| rs4773173A>G (*COL4A2*)_A |  |  |  | 0.53 | 0.70 | 0.45 |
| rs3812945T>C (*SCAMP5*)_T |  |  |  | 1.87 | 0.92 | **0.04** |
| rs28555129C>A (*OSGIN1; RP11-505K9.4*)_A |  |  |  | 0.78 | 0.78 | 0.32 |
| rs826682A>C (*LIMS1*)_A |  |  |  | 1.75 | 1.03 | 0.09 |
| rs14234A>G (*AC022201.5; FAM136A; SNRPG*)_A |  |  |  | 0.41 | 0.72 | 0.57 |
| rs17404153G>T (*DNAJC13*)_G |  |  |  | -0.36 | 1.19 | 0.76 |
| rs1030431G>A (*unknown*)_A |  |  |  | 0.20 | 0.62 | 0.75 |
| rs174583C>T (*FADS2*)_C |  |  |  | 0.72 | 0.78 | 0.36 |
| rs11136341A>G (*PLEC*)_A |  |  |  | -0.35 | 0.77 | 0.65 |
| rs1800562G>A (*HFE; HIST1H2BB*)_G |  |  |  | 22.60 | 1764.00 | 0.99 |
| rs11648003A>G (*DHODH*)_A |  |  |  | -2.32 | 1.04 | 0.03 |
| rs2920503C>T (*PPARG*)_C |  |  |  | 0.42 | 0.79 | 0.59 |
| rs9987289G>A (*RP11-115J16.1*)_A |  |  |  | -4.60 | 2.12 | **0.03** |

P-values in bold are significant. Abbreviation: BMI: body mass index; LDL-C: Low-density lipoprotein cholesterol; SE: standard error; SNP: single nucleotide polymorphism.

**S18 Table: Logistic models for early HDL-C decrease ≥5% in replication sample 2**

|  | **Model with clinical  variables** | | | **Model with clinical and  genetic variables** | | | |  |
| --- | --- | --- | --- | --- | --- | --- | --- | --- |
|  | **Estimate** | **SE** | **p-value** | | **Estimate** | **SE** | **p-value** | |
| (Intercept) | -1.13 | 1.20 | 0.35 | | -38.49 | 2721.00 | 0.99 | |
| Male gender | 0.23 | 0.52 | 0.67 | | -0.08 | 0.70 | 0.91 | |
| Baseline HDL-C levels (mmol/l) | 0.47 | 0.78 | 0.55 | | 0.32 | 1.00 | 0.75 | |
| Medication |  |  |  | |  |  |  | |
| Medium versus low risk | -0.80 | 0.63 | 0.21 | | -1.22 | 0.86 | 0.15 | |
| High versus low risk | 0.42 | 0.54 | 0.44 | | 0.20 | 0.66 | 0.76 | |
| SNP (*GENE*)_effect allele |  |  |  | |  |  |  | |
| rs12529923C>T (*GSTA10P; GSTA11P; GSTA3; GSTA5*)_C |  |  |  | | -0.03 | 0.46 | 0.94 | |
| rs17576323T>C (*AC007319.1*)_T |  |  |  | | 0.28 | 0.55 | 0.62 | |
| rs11727676T>C (*HHIP; uc_338*)_T |  |  |  | | -0.33 | 0.89 | 0.71 | |
| rs2683521G>A (*Metazoa_SRP; SP1*)_G |  |  |  | | 0.36 | 0.48 | 0.45 | |
| rs10494363G>A (*MTMR11; OTUD7B*)_G |  |  |  | | -0.20 | 0.95 | 0.84 | |
| rs10911505T>C (*unknown*)_T |  |  |  | | 0.41 | 0.51 | 0.42 | |
| rs1519480T>C (*BDNF; BDNF-AS*)_C |  |  |  | | 0.08 | 0.47 | 0.87 | |
| rs2373459T>C (*SPIC*)_C |  |  |  | | 0.55 | 0.49 | 0.26 | |
| rs1126930G>C (*PRKAG1; RP11-386G11.5*)_G |  |  |  | | 17.92 | 1361.00 | 0.99 | |
| rs2280334C>T (*MEIS1; MEIS1-AS2; MEIS1-AS3*)_T |  |  |  | | 0.12 | 0.41 | 0.78 | |
| rs7730898A>G (*RANBP17*)_G |  |  |  | | 0.38 | 0.51 | 0.46 | |
| rs3173615C>G (*TMEM106B*)_C |  |  |  | | 0.06 | 0.43 | 0.89 | |
| rs3822072G>A (*FAM13A*)_G |  |  |  | | 0.08 | 0.42 | 0.84 | |
| rs970548A>C (*MARCH8*)_A |  |  |  | | 0.00 | 0.58 | 1.00 | |
| rs7134594T>C (*MMAB*)_C |  |  |  | | 0.20 | 0.44 | 0.64 | |
| rs11246602T>C (*OR4C46*)_T |  |  |  | | 0.58 | 0.75 | 0.44 | |
| rs7134375C>A (*unknown*)_C |  |  |  | | 0.87 | 0.54 | 0.11 | |
| rs11869286C>G (*STARD3*)_G |  |  |  | | -0.21 | 0.44 | 0.63 | |
| rs4765127G>T (*CCDC92; FAM101A; RP11-214K3.18; RP11-214K3.25; ZNF664*)_G |  |  |  | | -0.26 | 0.49 | 0.59 | |

P-values in bold are significant. Abbreviation: HDL-C: High-density lipoprotein cholesterol; SE: standard error; SNP: single nucleotide polymorphism. Psychotropic drugs were categorized into three categories depending on their risk of inducing metabolic disturbances, as described in the method section.

**S19 Table: Logistic models for TG increase ≥5% in replication sample 2**

|  | **Model with clinical variables** | | | **Model with clinical and  genetic variables** | | |
| --- | --- | --- | --- | --- | --- | --- |
|  | **Estimate** | **SE** | **p-value** | **Estimate** | **SE** | **p-value** |
| (Intercept) | 0.83 | 0.44 | 0.06 | 2.15 | 2.18 | 0.33 |
| Male gender | 0.38 | 0.49 | 0.45 | 0.92 | 0.68 | 0.18 |
| Baseline TG levels (mmol/l) | -0.47 | 0.25 | 0.06 | -0.49 | 0.32 | 0.13 |
| SNP (*GENE*)_effect allele |  |  |  |  |  |  |
| rs7519429A>C (*DNM3; PIGC*)_A |  |  |  | 0.02 | 0.46 | 0.96 |
| rs4683438G>T (*RP11-372E1.4*)_G |  |  |  | 0.31 | 0.46 | 0.50 |
| rs3851294G>A (*DSTYK*)_A |  |  |  | -0.30 | 0.76 | 0.70 |
| rs1133400A>G (*INPP5A*)_A |  |  |  | 0.55 | 0.53 | 0.30 |
| rs6430090G>A (*AC092484.1*)_A |  |  |  | 1.15 | 0.49 | **0.02** |
| rs10199914A>G (*AC114788.1*)_A |  |  |  | 0.20 | 0.43 | 0.64 |
| rs382534C>T (*SLC33A1*)_C |  |  |  | -0.32 | 0.48 | 0.50 |
| rs12206516A>G (*unknown*)_A |  |  |  | -0.02 | 0.59 | 0.97 |
| rs13266634C>T (*SLC30A8*)_C |  |  |  | -0.79 | 0.50 | 0.11 |
| rs4738141A>G (*RP11-1102P16.1*)_A |  |  |  | 1.44 | 0.56 | **0.01** |
| rs3843935C>T (*PRSS3; TRBV29OR9-2; UBE2R2-AS1*)_C |  |  |  | 0.13 | 0.40 | 0.74 |
| rs2131925T>G (*DOCK7*)_G |  |  |  | -0.79 | 0.47 | 0.09 |
| rs17585887C>T (*unknown*)_T |  |  |  | -0.11 | 0.48 | 0.81 |
| rs1495741A>G (*unknown*)_G |  |  |  | -1.02 | 0.46 | **0.03** |
| rs731839A>G (*PEPD*)_G |  |  |  | -0.56 | 0.48 | 0.24 |
| rs72959041G>A (*RSPO3*)_G |  |  |  | -1.71 | 1.25 | 0.17 |

P-values in bold are significant. Abbreviation; SE: standard error; SNP: single nucleotide polymorphism; TG: triglycerides.

**S20 Table: Predictive power of models on new onset dyslipidemia including only clinical variables and including clinical plus genetic variables, in the combined psychiatric sample**

| **Dependent variable** | **Logistic  model** | **Sensitivity %  (95%CI)** | **Specificity %  (95%CI)** | **PPV %  (95%CI)** | **NPV %  (95%CI)** | **Accuracy %  (95%CI)** | **AUC  (95%CI)** | **P-value^3^** | **P-value Likelihood ratio test^4^** |
| --- | --- | --- | --- | --- | --- | --- | --- | --- | --- |
|  |  |  |  |  |  |  |  |  |  |
| **NODTC** | Clin^1^ | 0.72 (0.59-0.82) | 0.69 (0.6-0.81) | 0.56 (0.49-0.65) | 0.82 (0.77-0.88) | 0.7 (0.64-0.77) | 0.71 (0.63-0.79) | **0.04** | 0.24 |
|  | Clin + Gen ^2^ | 0.72 (0.61-0.82) | 0.77 (0.64-0.85) | 0.63 (0.53-0.73) | 0.83 (0.79-0.89) | 0.75 (0.68-0.81) | 0.77 (0.70-0.84) |  |  |
|  |  |  |  |  |  |  |  |  |  |
| **NODLDL** | Clin^1^ | 0.64 (0.53-0.83) | 0.73 (0.52-0.83) | 0.5 (0.41-0.61) | 0.83 (0.79-0.89) | 0.71 (0.6-0.77) | 0.71 (0.64-0.79) | **0.002** | **0.01** |
|  | Clin + Gen ^2^ | 0.8 (0.7-0.89) | 0.75 (0.66-0.82) | 0.57 (0.49-0.65) | 0.9 (0.85-0.94) | 0.76 (0.7-0.82) | 0.81 (0.75-0.87) |  |  |
|  |  |  |  |  |  |  |  |  |  |
| **NODHDL** | Clin^1^ | 0.75 (0.65-0.88) | 0.68 (0.57-0.78) | 0.35 (0.3-0.43) | 0.92 (0.9-0.96) | 0.69 (0.62-0.77) | 0.77 (0.71-0.83) | **0.01** | 0.49 |
|  | Clin + Gen ^2^ | 0.83 (0.72-0.91) | 0.73 (0.66-0.81) | 0.41 (0.36-0.49) | 0.95 (0.92-0.97) | 0.75 (0.69-0.81) | 0.82 (0.77-0.87) |  |  |
|  |  |  |  |  |  |  |  |  |  |
| **NODTG** | Clin^1^ | 0.75 (0.61-0.86) | 0.7 (0.57-0.84) | 0.31 (0.25-0.44) | 0.94 (0.91-0.97) | 0.71 (0.61-0.82) | 0.78 (0.72-0.86) | 0.07 | 0.07 |
|  | Clin + Gen ^2^ | 0.8 (0.66-0.91) | 0.75 (0.67-0.91) | 0.37 (0.3-0.58) | 0.95 (0.93-0.98) | 0.76 (0.69-0.88) | 0.84 (0.78-0.91) |  |  |

The combined psychiatric sample consisted of patients from the discovery and replication samples without baseline dyslipidemia for the corresponding lipid phenotype. NODTC: new onset total hypercholesterolemia (n=82/203); NODLDL: new onset LDL hypercholesterolemia (n=69/210); NODHDL: new onset HDL hypocholesterolemia (n=56/290); NODTG: new onset hypertriglyceridemia (n=36/225); PPV: positive predictive value; NPV: negative predictive value; AUC: area under the curve.

^1^ Logistic model including only clinical parameters selected after AIC selection using the discovery sample.

^2^ Logistic model including clinical parameters selected after AIC selection and SNPs selected after LASSO selection using the discovery sample.

^3^ P-values of difference between the AUC of the model containing clinical data and the model containing clinical and genetic data. 2000 bootstraps were used for the analysis.

^4^ P-values of likelihood ratio tests to compare the model including only the clinical variables and the model containing clinical and genetic variables. A significant p-value indicates an improved goodness of fit.

**S21 Table: Association between PsyMetab-derived genetic risk scores (psyGRS) and relative difference of corresponding lipid levels in subjects from the UK Biobank**

|  | **Participants receiving a psychotropic drug, referred to as participants with “psychotropic treatment”** | | | **Participants not taking any psychotropic drug, referred to as "control" subjects** | | |
| --- | --- | --- | --- | --- | --- | --- |
|  | **N** | **Estimate** | **p-value** | **N** | **Estimate** | **p-value** |
| **ΔTC** | 591 | 0.002 | 0.78 | 8287 | 0.001 | 0.67 |
| **ΔLDL** | 588 | -0.008 | 0.44 | 8245 | 0.000 | 0.95 |
| **ΔHDL** | 477 | 0.020 | 0.06 | 6690 | -0.004 | 0.15 |
| **ΔTG** | 589 | 0.017 | **0.04** | 8276 | -0.001 | 0.74 |

Δ indicates relative difference, calculated as the difference of second minus the first measure, divided by the first measure. Value in bold is significant.

**S1 Figure: Flowcharts of patient selection**

**a.**


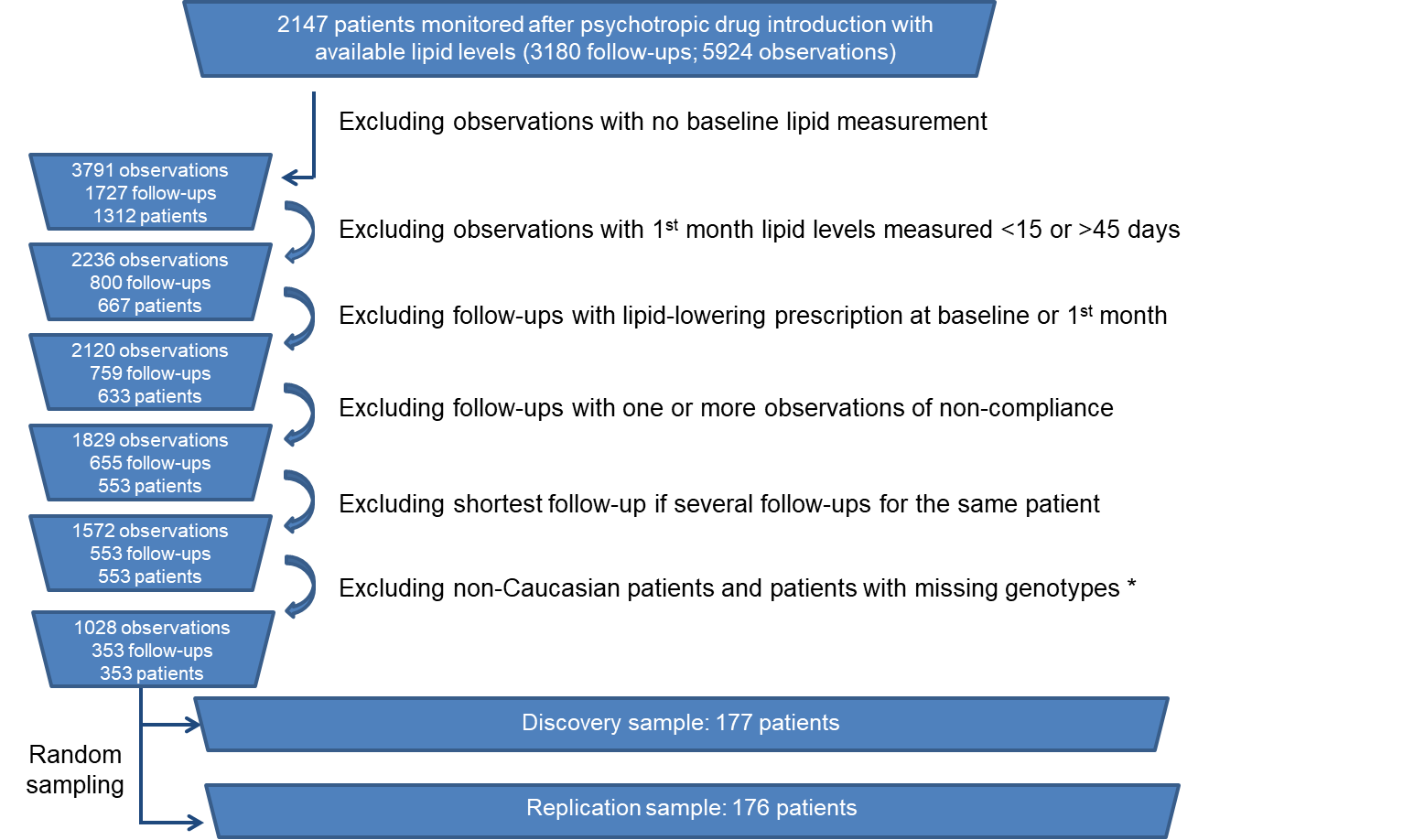


**b.**

**
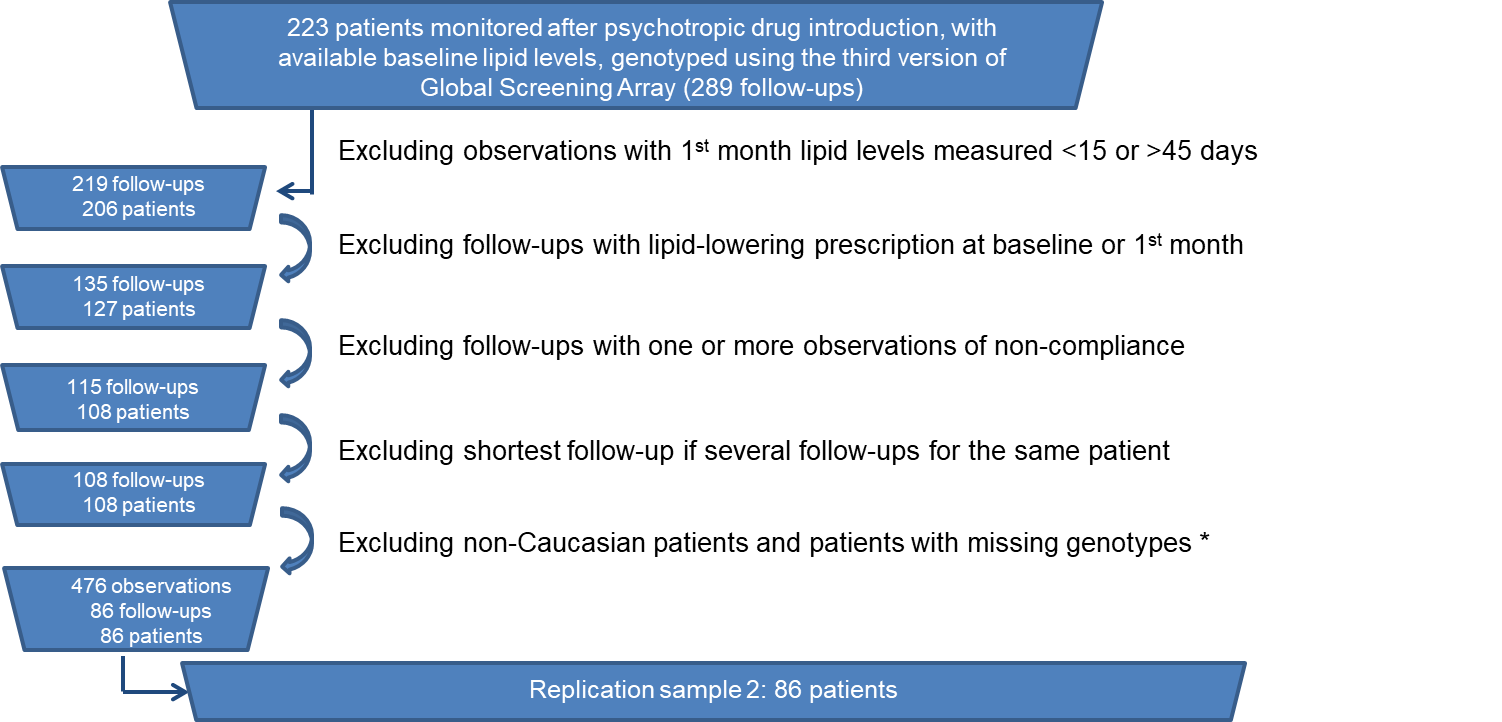
**

1. Selection of patients included in the discovery and in the replication sample 1.
2. Selection of patients included in the replication sample 2.

* Patients with any missing genotype for the SNPs included in the present study were excluded.


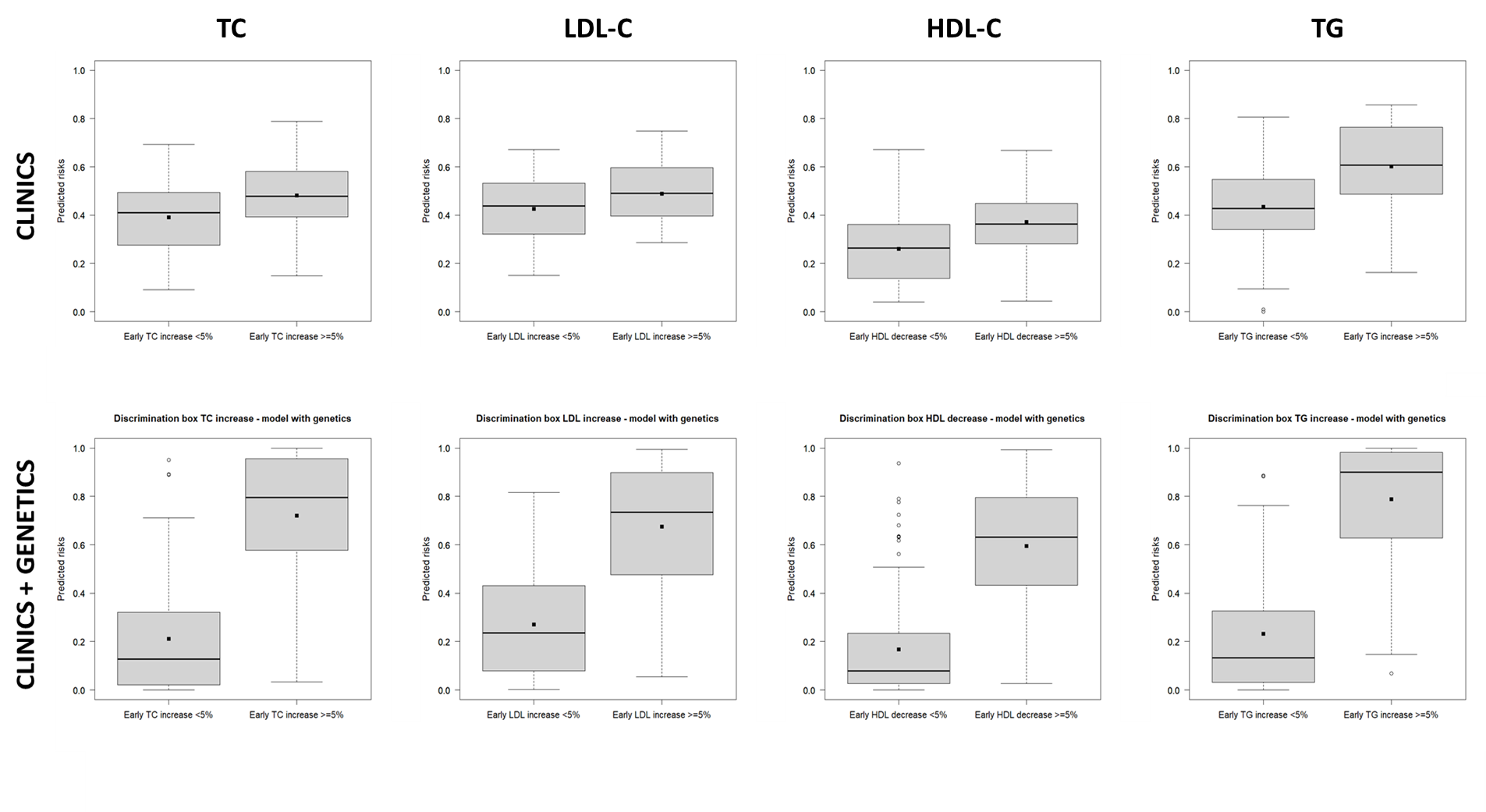


S2 Figure: Discrimination boxplots for early increases ≥5% of TC, LDL-C and TG and for early decrease ≥5% of HDL-C in the discovery sample. The upper part of the figure displays the distribution of predicted risks from models including only clinical factors, whereas its lower part shows the distribution of predicted risks from models including clinical plus genetic factors. Abbreviation: HDL-C: high-density lipoprotein cholesterol; LDL-C: low-density lipoprotein cholesterol; TC: total cholesterol; TG: triglyceride.


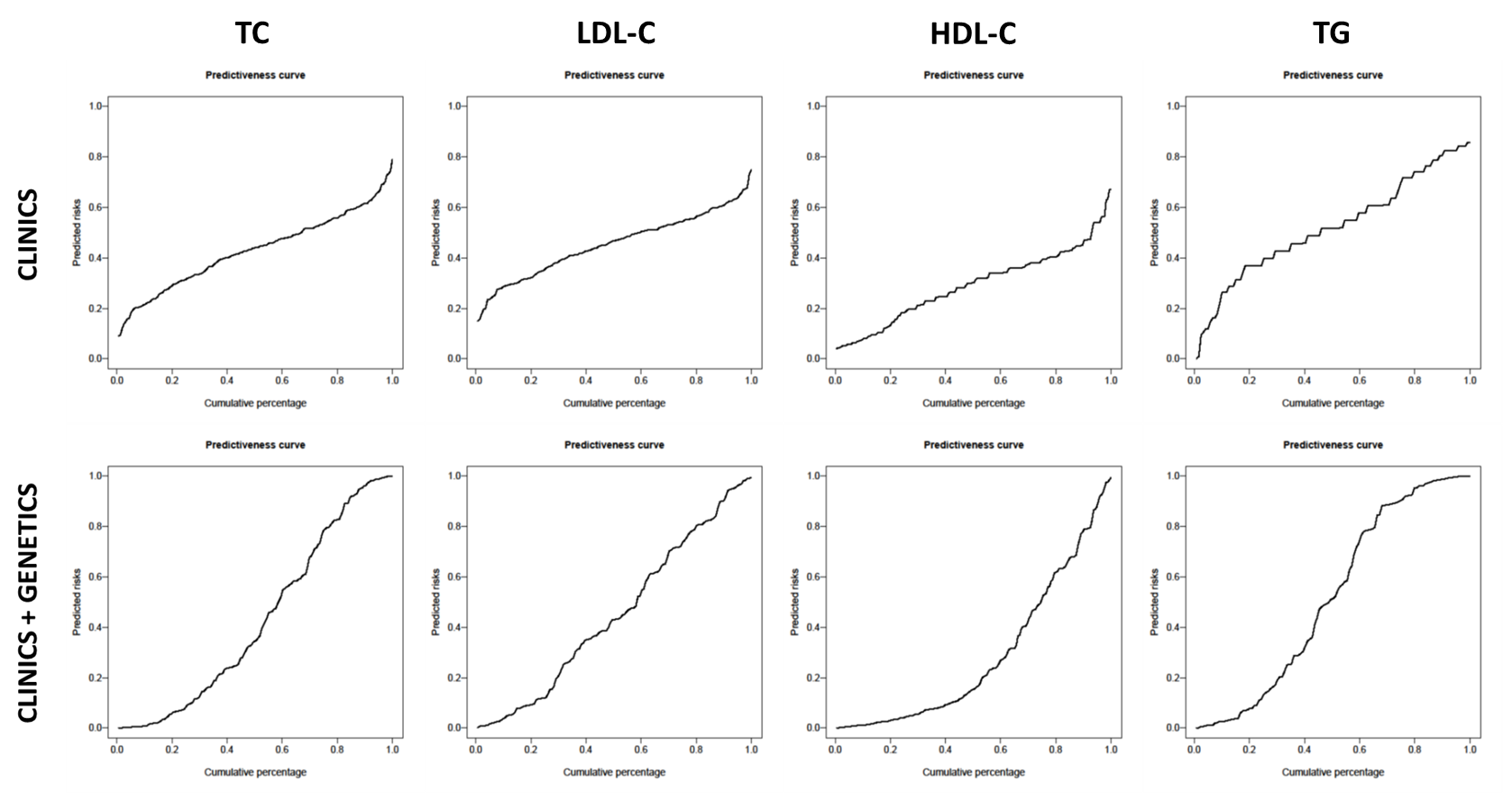


S3 Figure: Predictiveness curves for early increases ≥5% of TC, LDL-C and TG and for early decrease ≥5% of HDL-C in the discovery sample. These curves plot the cumulative percentage of patients to the predicted risks for early lipid levels worsening during the first month of psychotropic treatment. The upper part of the figure displays predictiveness curves from models including only clinical factors, whereas the lower part shows predictiveness curves from models including clinical plus genetic factors. Abbreviation: HDL-C: high-density lipoprotein cholesterol; LDL-C: low-density lipoprotein cholesterol; TC: total cholesterol; TG: triglyceride.


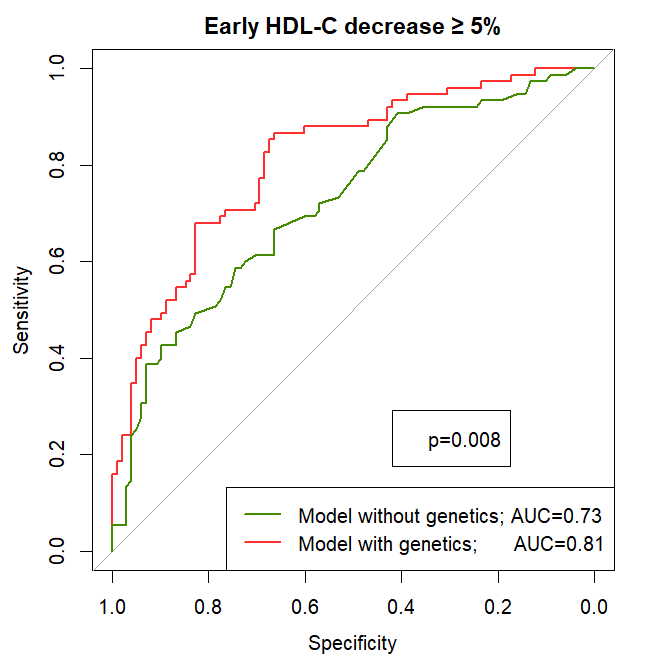

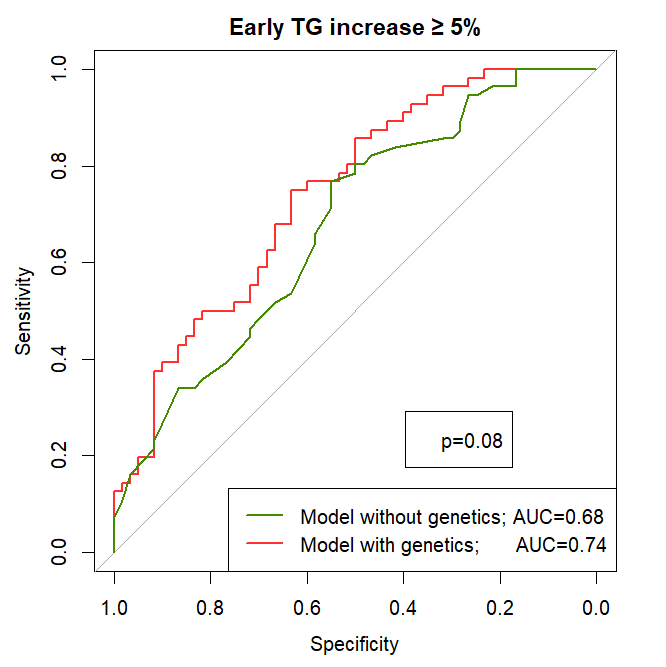

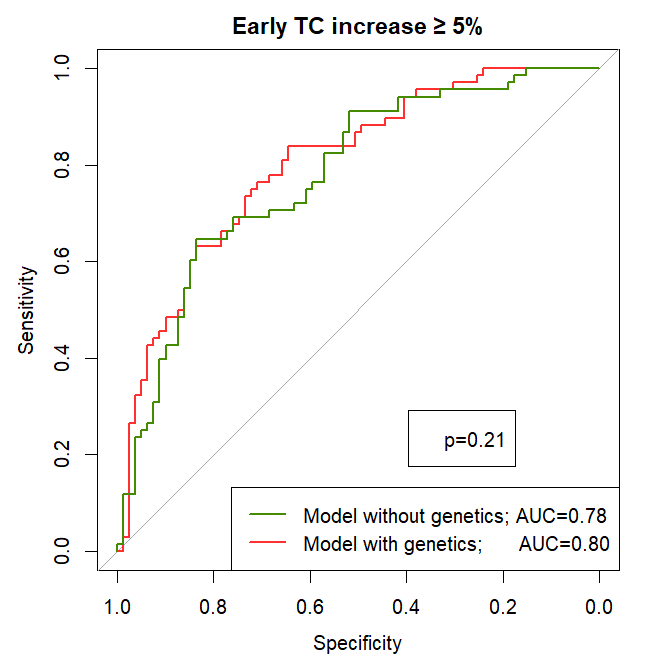

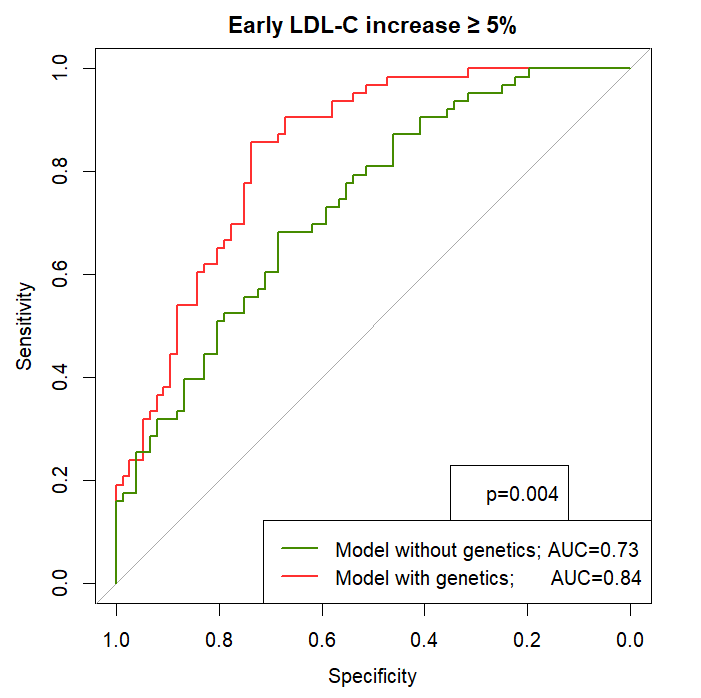


**S4 Figure Receiver operating characteristic curves for early worsening of lipid levels in the replication sample 1.**

Red curves correspond to models including clinical and genetics factors, whereas green curves include only clinical factors. Only fasting patients were included for TG analyses. Abbreviation: AUC, area under the curve; HDL, high-density lipoprotein cholesterol; LDL, low-density lipoprotein cholesterol; TC, total cholesterol; TG, triglyceride.


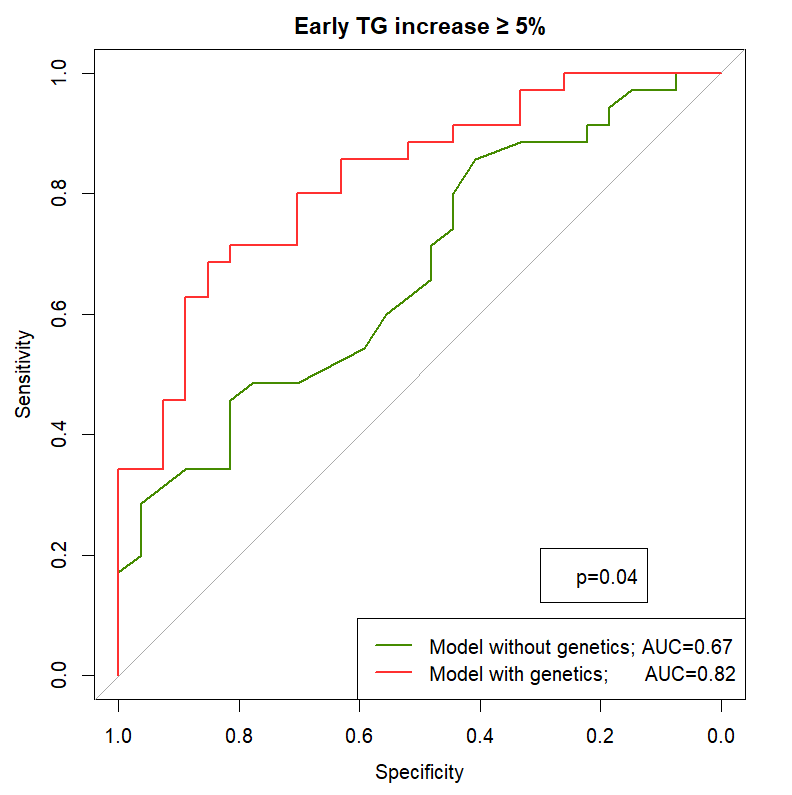

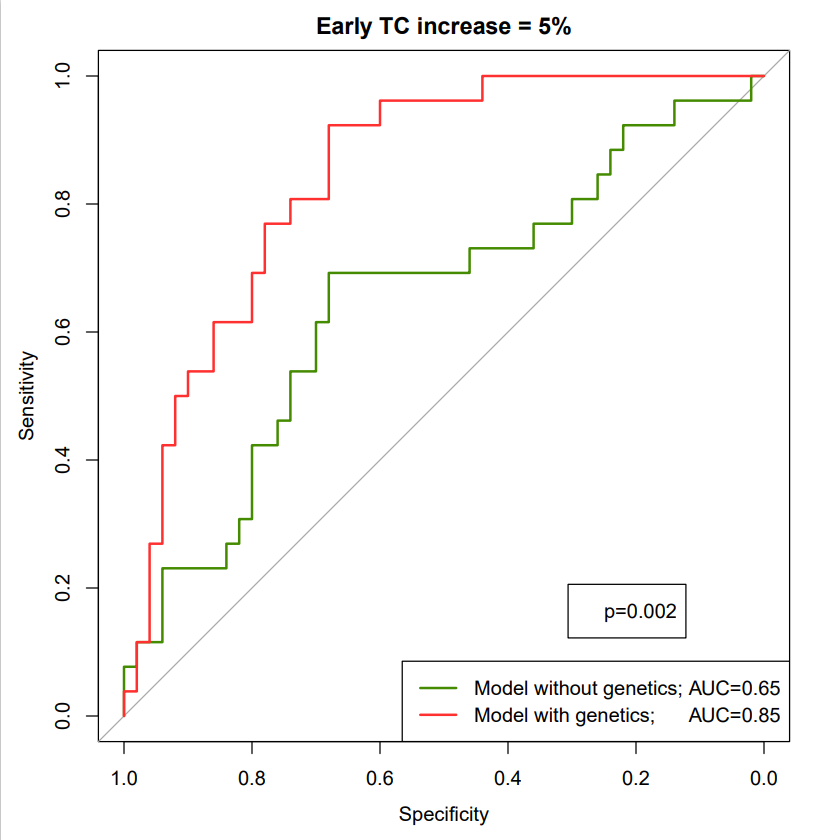

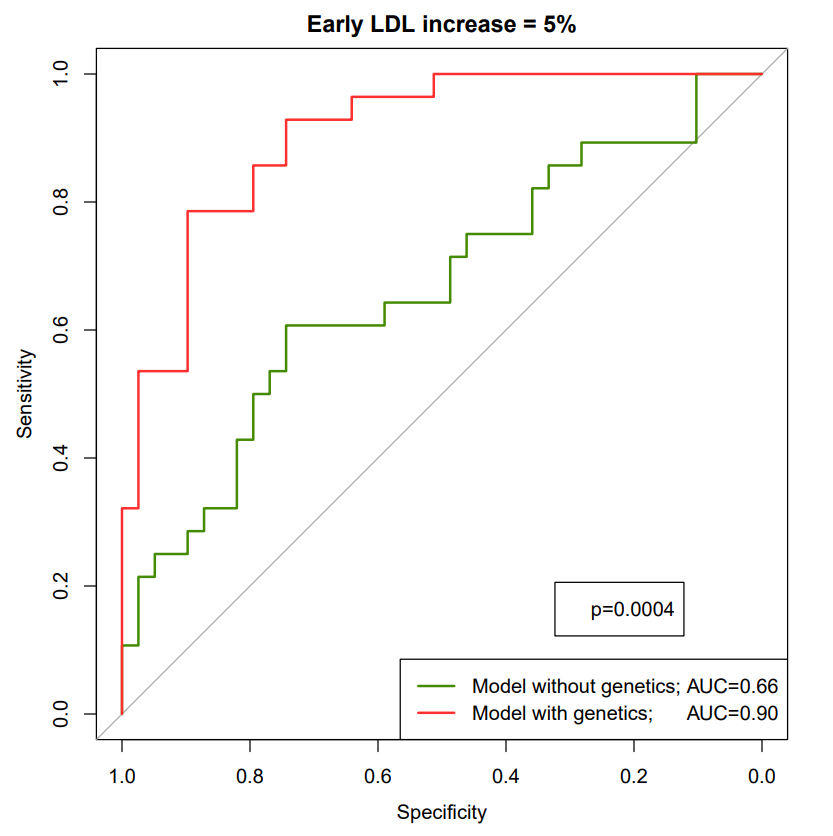

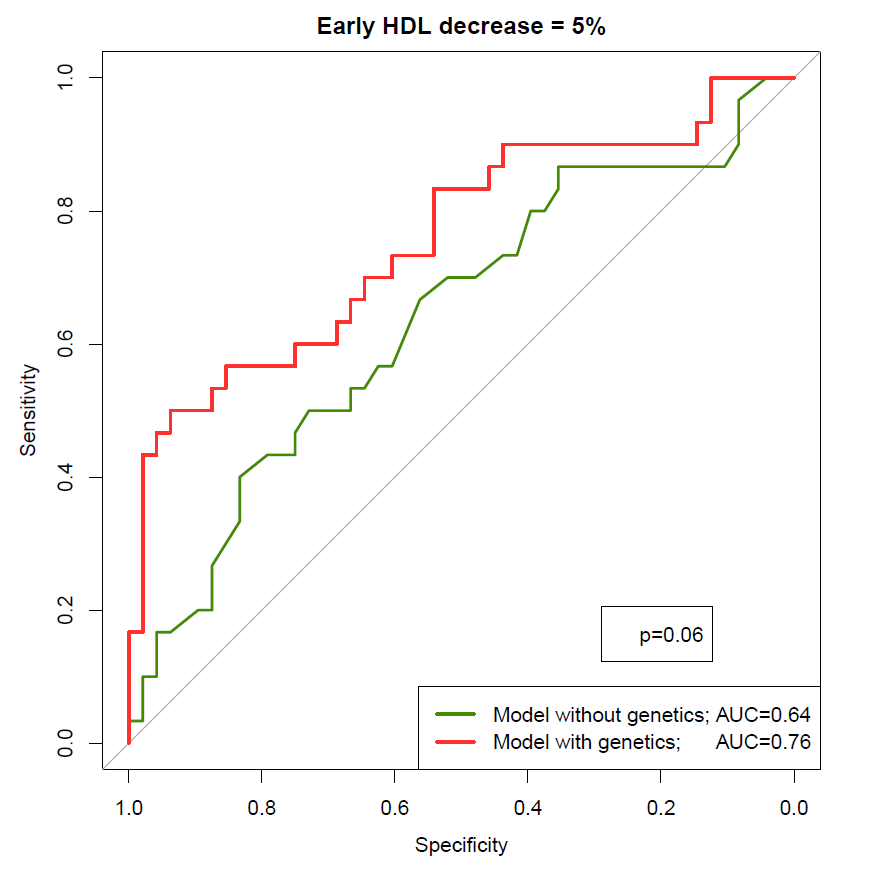

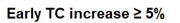

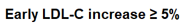

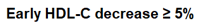

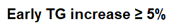


**S5 Figure Receiver operating characteristic curves for early deterioration of lipid levels in the replication sample 2.**

Red curves correspond to models including clinical and genetics factors, whereas green curves include only clinical factors. Only fasting patients were included for TG analyses. Abbreviation: AUC, area under the curve; HDL, high-density lipoprotein cholesterol; LDL, low-density lipoprotein cholesterol; TC, total cholesterol; TG, triglyceride.


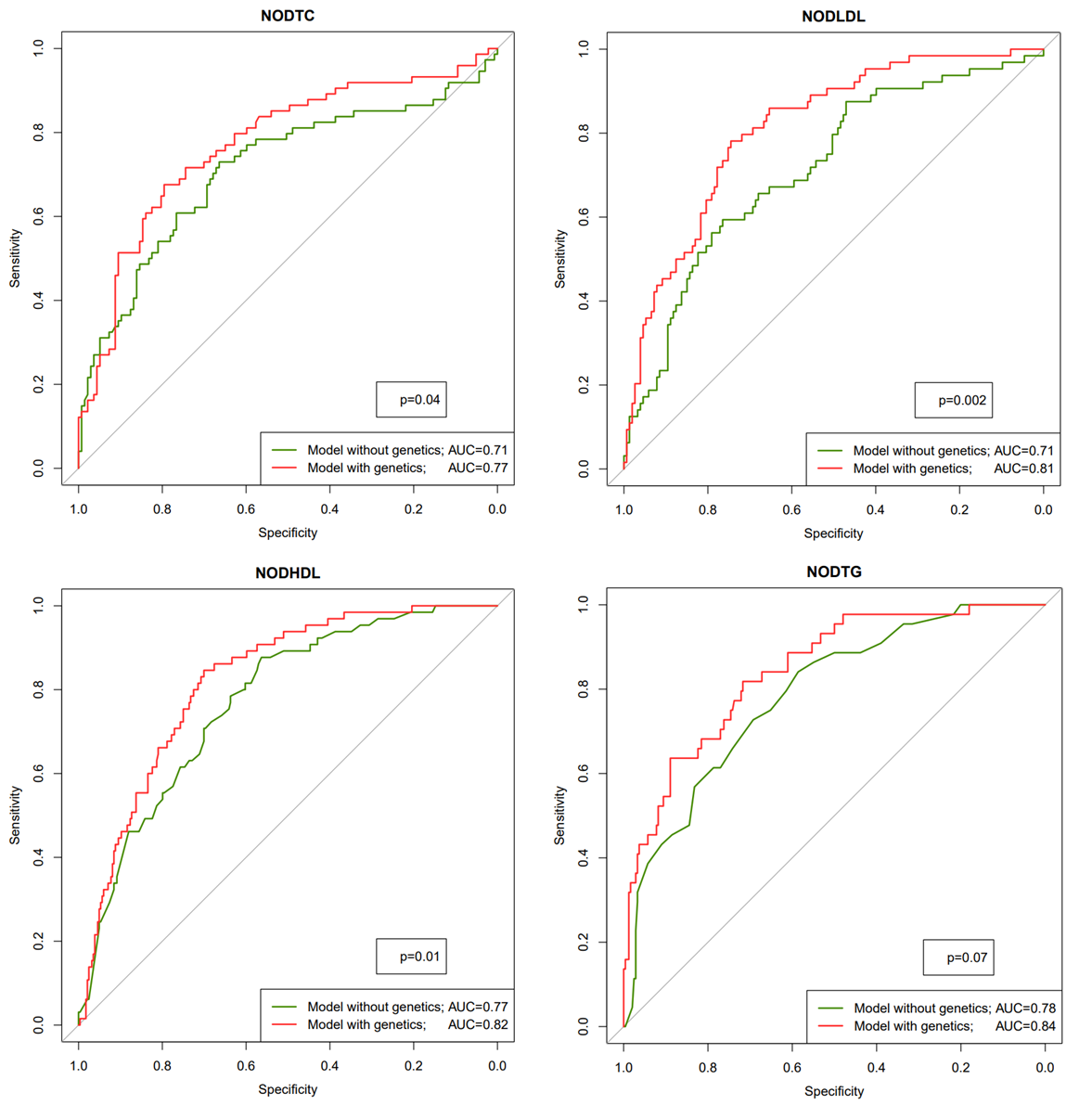


**S6 Figure Receiver operating characteristic curves for new onset dyslipidemia in the combined psychiatric sample**

The combined psychiatric sample consisted of patients from the discovery and replication samples without baseline dyslipidemia for the corresponding lipid phenotype. Red curves correspond to models including clinical and genetics components, whereas green curves include only clinical variables. Only fasting patients were included for TG analyses. AUC, area under the curve; NODTC: new onset total hypercholesterolemia (n=82/203); NODLDL: new onset LDL hypercholesterolemia (n=69/210); NODHDL: new onset HDL hypocholesterolemia (n=56/290); NODTG: new onset hypertriglyceridemia (n=36/225).


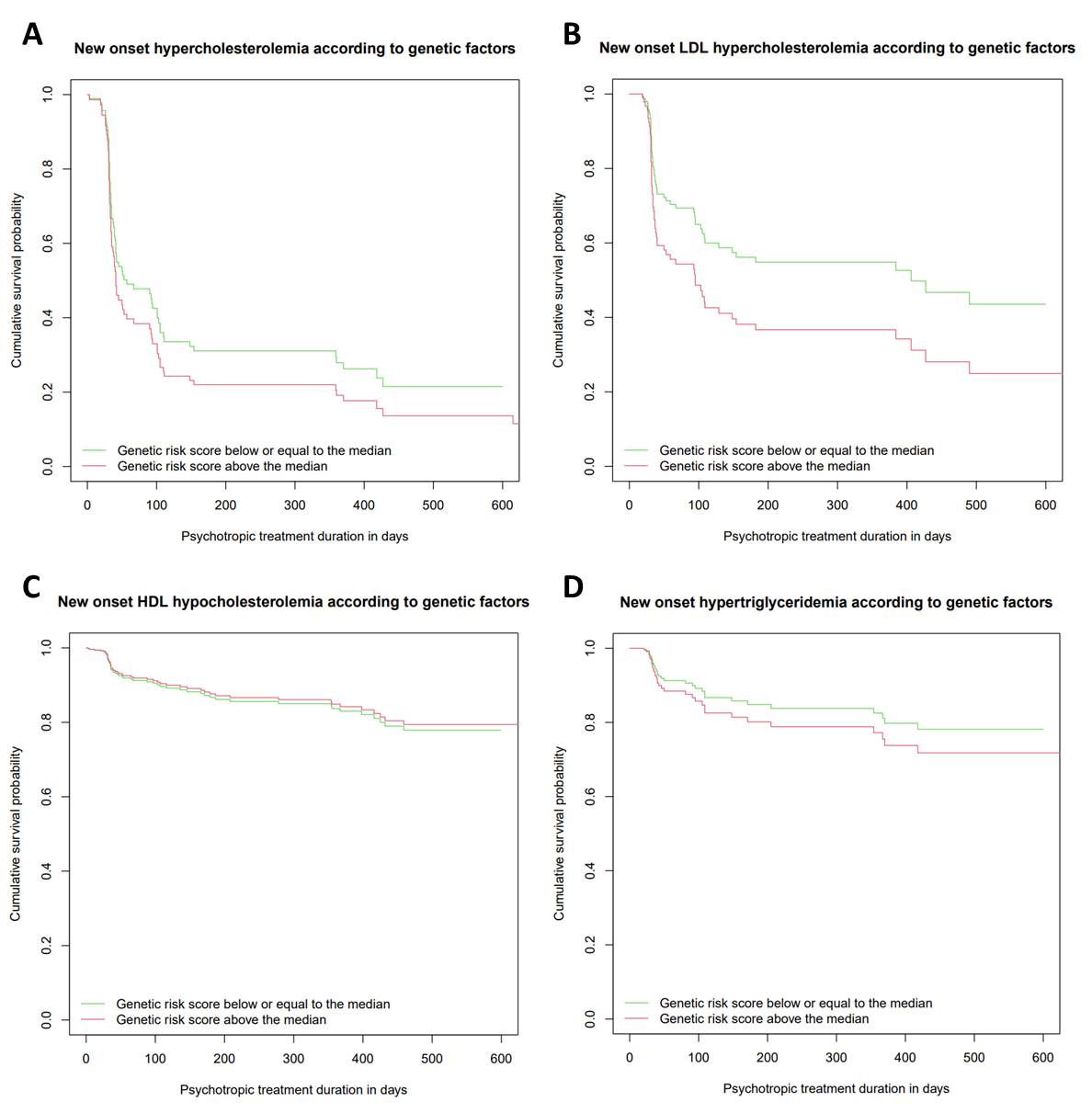


**S7 Figure Survival curves for new onset dyslipidemia according to genetic risk score group (psyGRS) in patients with fixed clinical parameters**

Survival curves were computed from Cox proportional hazard models.

A Survival curves for NODTC (new onset TC hypercholesterolemia) in non-smoking patients with mean BMI and mean baseline TC levels, according to TC psyGRS groups.

B Survival curves for NODLDL (new onset LDL hypercholesterolemia) in non-smoking patients with mean BMI and mean baseline LDL-C levels, according to LDL-C psyGRS groups.

C Survival curves for NODHDL (new onset HDL hypocholesterolemia) in women taking a medium risk drug, with mean baseline HDL-C levels, according to HDL-C psyGRS groups.

D Survival curves for NODTG (new onset hypertriglyceridemia) in women with mean baseline TG levels, according to TG psyGRS groups.

PsyGRSs consisted of the combination of SNPs retained in LASSO models for the corresponding lipid phenotypes (n=17, 18, 19 and 16 for TC, LDL-C, HDL-C and TG, respectively). For each lipid phenotype, green curve refers to patients whose genetic risk score was lower or equal to the median of the genetic risk score, while red curve refers to patients whose genetic risk score was higher than the median of the genetic risk score.


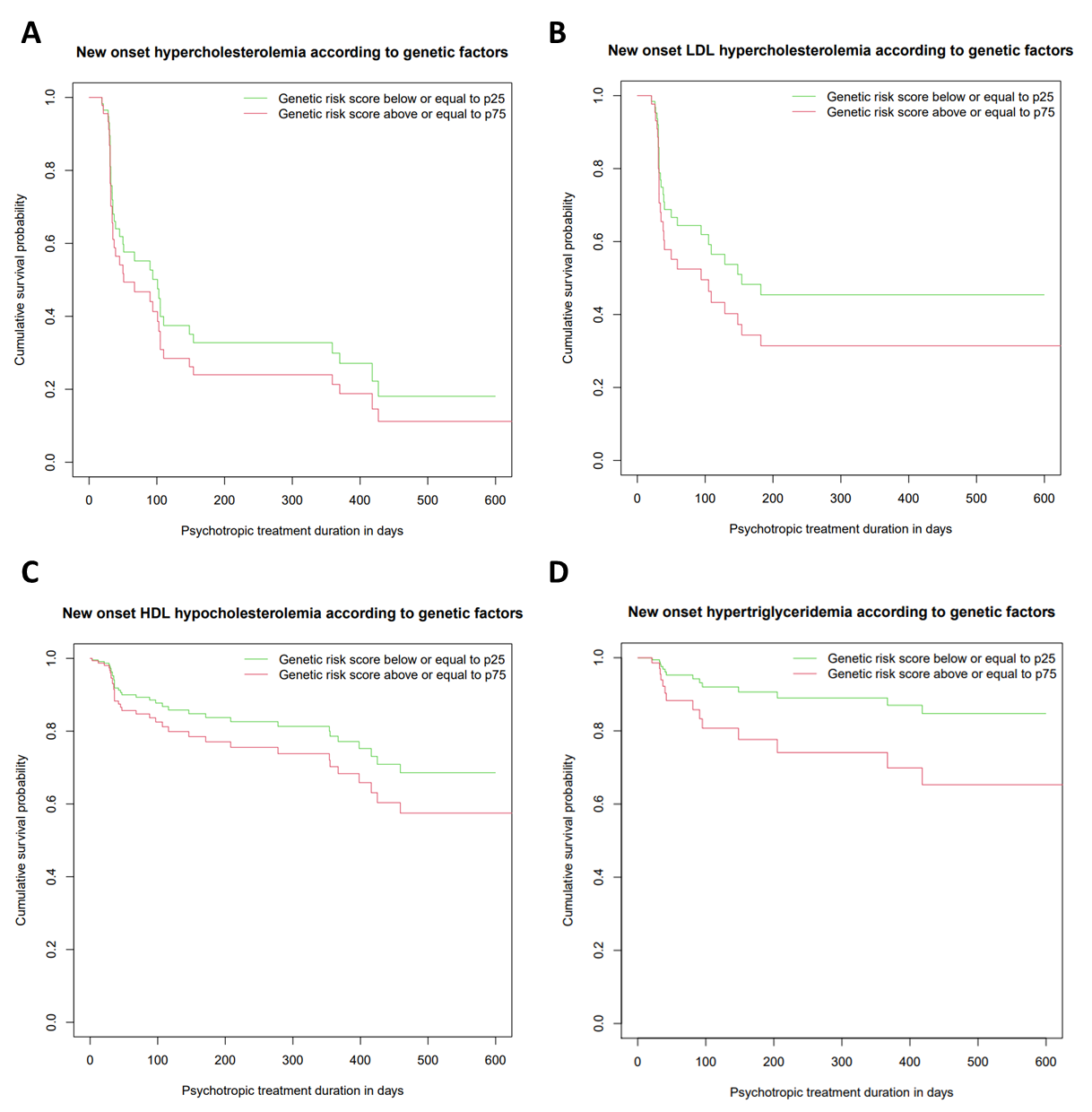


**S8 Figure Survival curves for new onset dyslipidemia according to genetic risk score group (psyGRS) in patients with fixed clinical parameters**

Survival curves were computed from Cox proportional hazard models.

A Survival curves for NODTC (new onset TC hypercholesterolemia) in non-smoking patients with mean BMI and mean baseline TC levels, according to TC psyGRS groups.

B Survival curves for NODLDL (new onset LDL hypercholesterolemia) in non-smoking patients with mean BMI and mean baseline LDL-C levels, according to LDL-C psyGRS groups.

C Survival curves for NODHDL (new onset HDL hypocholesterolemia) in women taking a medium risk drug, with mean baseline HDL-C levels, according to HDL-C psyGRS groups.

D Survival curves for NODTG (new onset hypertriglyceridemia) in women with mean baseline TG levels, according to TG psyGRS groups.

PsyGRSs consisted of the combination of SNPs retained in LASSO models for the corresponding lipid phenotypes (n=17, 18, 19 and 16 for TC, LDL-C, HDL-C and TG, respectively). For each lipid phenotype, green curve refers to patients whose genetic risk score is lower or equal to the 1^st^ quartile of the genetic risk score for the corresponding lipid phenotype, while red curve refers to patients whose genetic risk score is higher than the 3^rd^ quartile of the genetic risk score for the corresponding lipid phenotype.


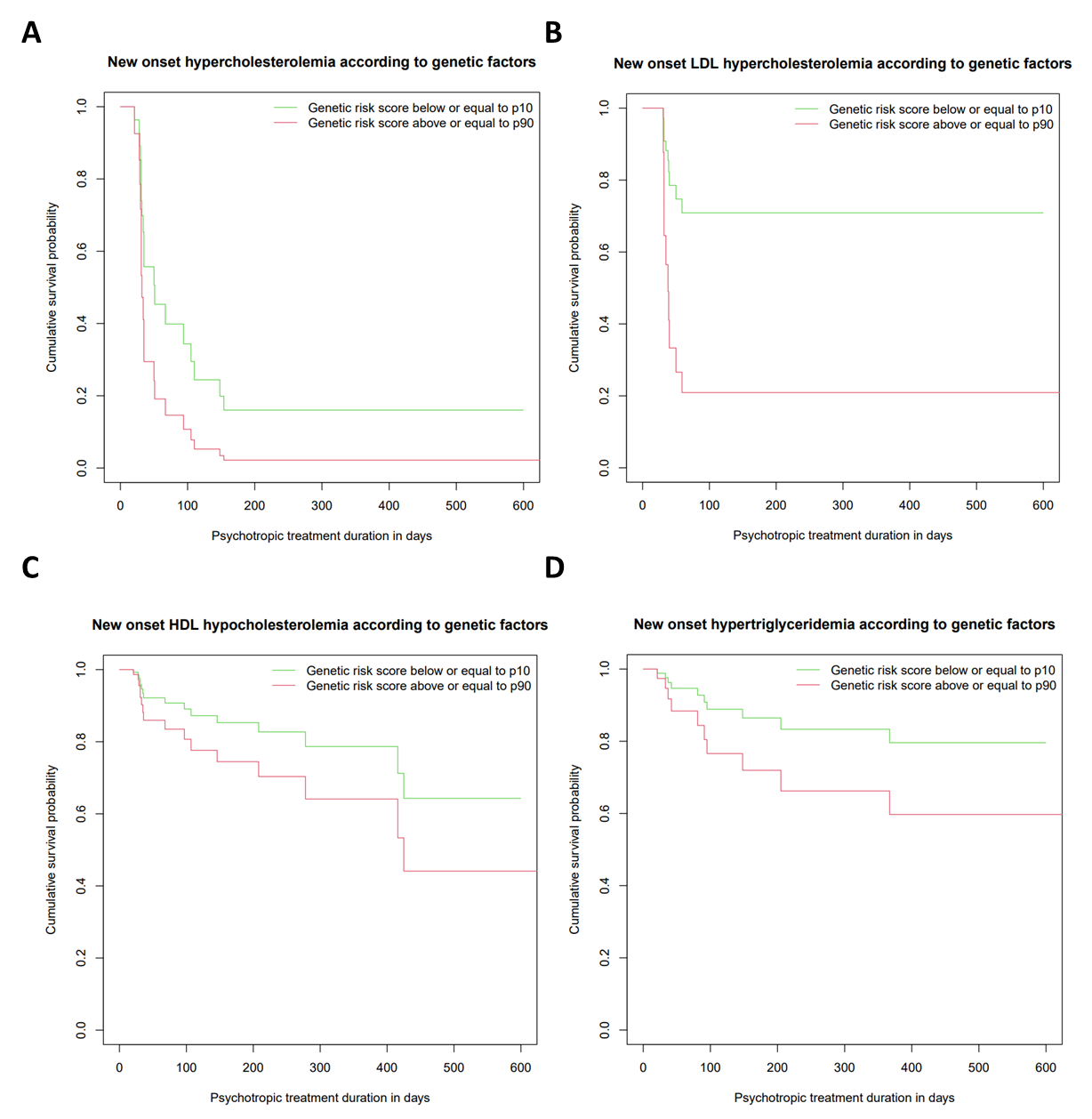


**S9 Figure Survival curves for new onset dyslipidemia according to genetic risk score group (psyGRS) in patients with fixed clinical parameters**

Survival curves were computed from Cox proportional hazard models.

A Survival curves for NODTC (new onset TC hypercholesterolemia) in non-smoking patients with mean BMI and mean baseline TC levels, according to TC psyGRS groups.

B Survival curves for NODLDL (new onset LDL hypercholesterolemia) in non-smoking patients with mean BMI and mean baseline LDL-C levels, according to LDL-C psyGRS groups.

C Survival curves for NODHDL (new onset HDL hypocholesterolemia) in women taking a medium risk drug, with mean baseline HDL-C levels, according to HDL-C psyGRS groups.

D Survival curves for NODTG (new onset hypertriglyceridemia) in women with mean baseline TG levels, according to TG psyGRS groups.

PsyGRSs consisted of the combination of SNPs retained in LASSO models for the corresponding lipid phenotypes (n=17, 18, 19 and 16 for TC, LDL-C, HDL-C and TG, respectively). For each lipid phenotype, green curve refers to patients whose genetic risk score is lower or equal to the 1^st^ decile of the genetic risk score for the corresponding lipid phenotype, while red curve refers to patients whose genetic risk score is higher than the 9^th^ decile of the genetic risk score for the corresponding lipid phenotype.

**Completed TRIPOD+AI checklist**

**
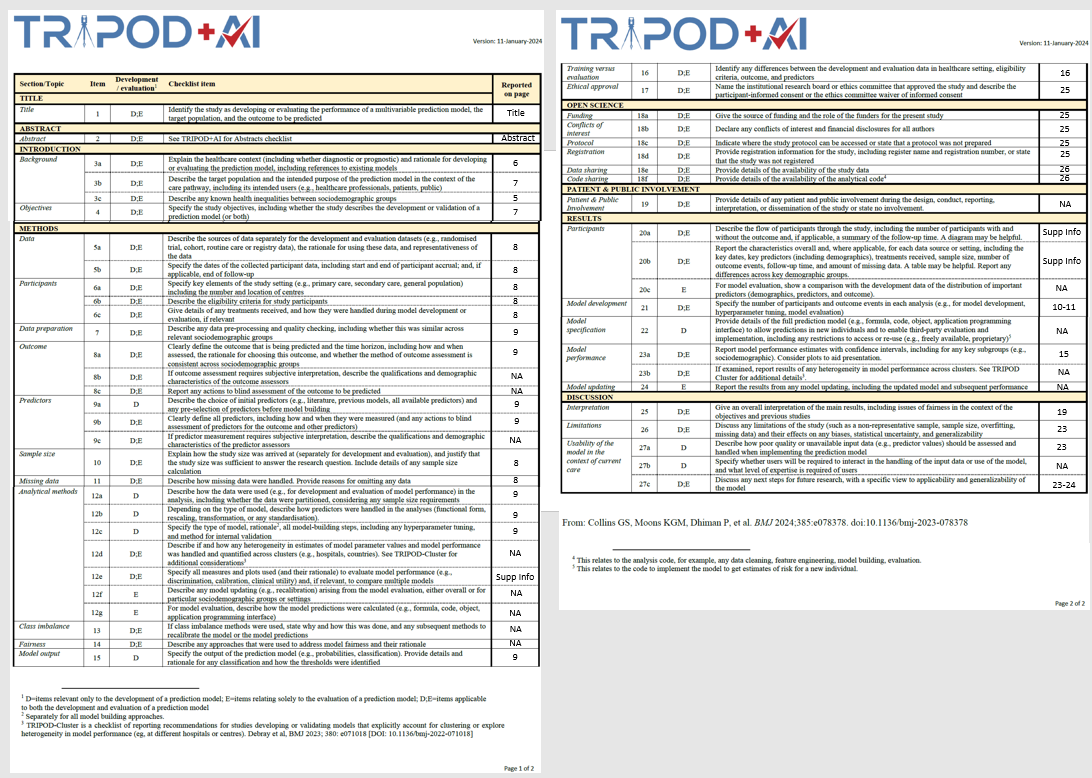
**

REFERENCES

1. Hiemke C, Baumann P, Bergemann N, Conca A, Dietmaier O, Egberts K, et al. AGNP Consensus Guidelines for Therapeutic Drug Monitoring in Psychiatry: Update 2011. Pharmacopsychiatry. 2011;44(6):195-235.

2. Leucht S, Cipriani A, Spineli L, Mavridis D, Orey D, Richter F, et al. Comparative efficacy and tolerability of 15 antipsychotic drugs in schizophrenia: a multiple-treatments meta-analysis. Lancet. 2013;382(9896):951-62.

3. Correll CU, Detraux J, De Lepeleire J, De Hert M. Effects of antipsychotics, antidepressants and mood stabilizers on risk for physical diseases in people with schizophrenia, depression and bipolar disorder. World psychiatry : official journal of the World Psychiatric Association. 2015;14(2):119-36.

4. Choong E, Quteineh L, Cardinaux JR, Gholam-Rezaee M, Vandenberghe F, Dobrinas M, et al. Influence of CRTC1 polymorphisms on body mass index and fat mass in psychiatric patients and the general adult population. JAMA psychiatry. 2013;70(10):1011-9.

5. Chen CY, Pollack S, Hunter DJ, Hirschhorn JN, Kraft P, Price AL. Improved ancestry inference using weights from external reference panels. Bioinformatics. 2013;29(11):1399-406.

6. Zhao S, Jing W, Samuels DC, Sheng Q, Shyr Y, Guo Y. Strategies for processing and quality control of Illumina genotyping arrays. Briefings in bioinformatics. 2018;19(5):765-75.

7. Chang CC, Chow CC, Tellier LC, Vattikuti S, Purcell SM, Lee JJ. Second-generation PLINK: rising to the challenge of larger and richer datasets. GigaScience. 2015;4:7.

8. Turner S, Armstrong LL, Bradford Y, Carlson CS, Crawford DC, Crenshaw AT, et al. Quality control procedures for genome-wide association studies. Current protocols in human genetics. 2011;Chapter 1:Unit1.19.

9. Laurie CC, Doheny KF, Mirel DB, Pugh EW, Bierut LJ, Bhangale T, et al. Quality control and quality assurance in genotypic data for genome-wide association studies. Genetic epidemiology. 2010;34(6):591-602.

10. McCarthy S, Das S, Kretzschmar W, Delaneau O, Wood AR, Teumer A, et al. A reference panel of 64,976 haplotypes for genotype imputation. Nature genetics. 2016;48(10):1279-83.

11. Das S, Forer L, Schönherr S, Sidore C, Locke AE, Kwong A, et al. Next-generation genotype imputation service and methods. Nature genetics. 2016;48(10):1284-7.

12. Manichaikul A, Mychaleckyj JC, Rich SS, Daly K, Sale M, Chen WM. Robust relationship inference in genome-wide association studies. Bioinformatics. 2010;26(22):2867-73.

13. Hajian-Tilaki K. Receiver Operating Characteristic (ROC) Curve Analysis for Medical Diagnostic Test Evaluation. Caspian journal of internal medicine. 2013;4(2):627-35.

14. Janssens AC, Moonesinghe R, Yang Q, Steyerberg EW, van Duijn CM, Khoury MJ. The impact of genotype frequencies on the clinical validity of genomic profiling for predicting common chronic diseases. Genetics in medicine : official journal of the American College of Medical Genetics. 2007;9(8):528-35.

15. Hanley JA, McNeil BJ. A method of comparing the areas under receiver operating characteristic curves derived from the same cases. Radiology. 1983;148(3):839-43.

16. de Graaff LC, van Schaik RH, van Gelder T. A clinical approach to pharmacogenetics. The Netherlands journal of medicine. 2013;71(3):145-52.
